# Efficacy of pembrolizumab and vorinostat combination in patients with recurrent and/or metastatic squamous cell carcinomas: a phase 2 basket trial

**DOI:** 10.1038/s43018-025-01004-2

**Published:** 2025-06-30

**Authors:** Edith Borcoman, Bastien Cabarrou, Miguel Francisco, Frédéric Bigot, François Ghiringhelli, Damien Vansteene, François Legrand, Maral Halladjian, Célia Dupain, Olivia Le Saux, Clelia Coutzac, Christian Borel, Raphael Chaltiel, Benoit You, Carlos Gomez-Roca, Sophie Cousin, Elodie Coquan, Aurélien Lambert, Esma Saada-Bouzid, Xavier Durando, Mathilde Saint-Ghislain, Gianmaria Frige, Elena Guerini-Rocco, Maria Manuela Tonini, Ivan Bièche, Zahra Castel-Ajgal, Grégoire Marret, Marie-Paule Sablin, Emmanuelle Jeannot, Fabrice Andre, Thomas Filleron, Marta Jimenez, Luca Mazzarella, Nicolas Servant, Maud Kamal, Christophe Le Tourneau

**Affiliations:** 1 Department of Drug Development and Innovation (D3i), Institut Curie, Paris, France; 2 INSERM U932, Immunity and Cancer, Institut Curie, Paris, France; 3 Translational Immunotherapy Team, Translational Research Department, Institut Curie, Paris, France; 4 Université Paris Sciences Lettres (PSL), Paris, France; 5 Biostatistics & Health Data Science Unit, Oncopole Claudius Regaud, Toulouse, France; 6 INSERM U900, Institut Curie, Mines Paris Tech, Paris, France; 7 Paris-Saclay University, Paris, France; 8 Institut de Cancérologie de l’Ouest, Angers, France; 9 Centre Georges-François Leclerc, Dijon, France; 10 Institut de Cancérologie de l’Ouest, Saint-Herblain, France; 11 Unicancer, Paris, France; 12 Medical Oncology, Centre Léon Berard, Lyon, France; 13 Centre Paul Strauss, Strasbourg, France; 14 Institut Godinot, Reims, France; 15 Medical Oncology, CITOHL, EPSILYON, IC-HCL, Lyon University Hospital (HCL), University Lyon 1, Lyon, France; 16 Institut Claudius Regaud, IUCT-Oncopole, Toulouse, France; 17 Institut Bergonié, Bordeaux, France; 18 Centre François Baclesse, Caen, France; 19 Institut de Cancerologie de Lorraine, Vandœuvre-Lès-Nancy, France; 20 Medical Oncology Department, Centre Antoine Lacassagne, Nice, France; 21 Laboratory of Translational Research in Oncology, Cote D’Azur University, Nice, France; 22 Department of Medical Oncology, Centre Jean Perrin, Clermont-Ferrand, France; 23 Experimental Oncology, Instituto Europeo di Oncologia, Milan, Italy; 24 Division of Pathology, European Institute of Oncology IRCCS, Milan, Italy; 25 Department of Oncology and Hemato-Oncology, European University of Milan, Milan, Italy; 26 Translational Medicine Operations Hub, Luxembourg Institute of Health, Strassen, Luxembourg; 27 Department of Genetics, Institut Curie, Paris, France; 28 Department of Pathology, Institut Curie, Paris, France; 29 Gustave Roussy, Villejuif, France; 30 Laboratory of Translational Oncology, European Institute of Oncology IRCCS, Milano, Italy; 31 Division of Gatrointestinal Oncology, European Institute of Oncology IRCCS, Milano, Italy; 32 Division of Advanced Molecular Diagnostics (DIMA), European Institute of Oncology IRCCS, Milano, Italy

**Keywords:** Cancer, Phase II trials, Molecular medicine, Cancer therapy

## Abstract

Immune checkpoint inhibitors improve the treatment of many solid tumors and have shown encouraging results in advanced squamous cell carcinoma (SCC), yet only a minority of patients respond to immune checkpoint inhibitor monotherapy. We conducted the PEVOsq trial, an open-label, nonrandomized, multicenter, basket phase 2 trial to evaluate the combination of pembrolizumab and vorinostat in recurrent/metastatic SCC of various origins. The primary endpoint was the objective response rate (ORR) in each tumor cohort during treatment as per the investigators’ assessment. Secondary endpoints included safety and antitumor activity evaluation in terms of centrally confirmed ORR, progression-free survival, overall survival and duration of response. In the efficacy population (*n* = 107), the ORR was met in cervical (39%), anal (31%) and vulvar/vaginal (19%) cancer cohorts, but not in head and neck SCC (19%) or penile (18%) cancer cohorts (overall ORR = 26%). Median progression-free survival was 4.0 months (95% confidence interval: 2.6–4.3), and median overall survival was 11.1 months (95% confidence interval: 9.2–17.4). In the safety population, 101 (91%) of 111 patients developed at least one treatment-related adverse event, with 39% and 5.4% of patients experiencing at least one grade 3 and grade 4 treatment-related adverse event, respectively. Vorinostat-related toxicity prompted a dose reduction/interruption in 66% of patients. Whole-exome sequencing analyses revealed several potential predictive biomarkers of response to treatment. Further studies in a larger number of patients are required to validate these findings. ClinicalTrials.gov identifier: NCT04357873.

## Abstract (web summary)

Borcoman and colleagues present the efficacy and safety results of the phase 2 PEVOsq basket trial investigating the combination of pembrolizumab with the epidrug vorinostat in patients with recurrent and/or metastatic squamous cell carcinoma.

## Main

Squamous cell carcinomas (SCCs) are among the most frequent solid tumors in humans^1^. SCCs primarily affect the lungs, cervix and head and neck (HNSCC). Less often, SCCs can also originate from other locations, including the penile, vulvar/vaginal and anal regions. Human papillomavirus (HPV) infection remains an important risk factor as HPV16 and/or HPV18 DNA is detected in about 90% of anal, 80% of vaginal, 70% of cervical and 50% of penile cancers and 30% of oropharyngeal SCCs^2,3^. Most other SCC cases are linked to environmental factors such as UV exposure, smoking and alcohol consumption, pointing toward common determinants in the etiology of all SCCs^1^.

Despite the advent of immunotherapies, recurrent and/or metastatic SCCs show poor prognosis with limited therapeutic options^4–6^. Immune checkpoint inhibitors (ICIs) such as pembrolizumab and nivolumab that block the programmed cell death protein-1 (PD-1) receptor have become standard first- and/or second-line treatments for patients with advanced SCC of the lung or cervix and HNSCC^7–12^. In addition, available results from phase 1 and 2 trials also show antitumoral activity for anal^13–16^, vulvar/vaginal^17,18^ and penile SCCs^19–21^. Nonetheless, only a minority of patients treated with ICI monotherapy achieve a durable response, with overall response rates (ORRs) ranging from 15% to 24%^7–13^, underscoring the need for novel therapeutic strategies to improve the efficacy of ICI agents.

One of the challenges associated with the development of new therapeutic regimens resides with the identification of reliable biomarkers of ICI efficacy to improve the patient selection process. In clinical routine, expression of programmed cell death-ligand 1 (PD-L1) is used as a biomarker. However, its transferability is limited as it is not used for all approved ICI agents. For instance, nivolumab prescription is not related to PD-L1 status^7,9^, whereas pembrolizumab administration is often conditioned by PD-L1 positivity^8,11,12^.

DNA mismatch repair (dMMR) deficiency leads to microsatellite instability (MSI) due to the accumulation of errors and increases the number of somatic mutations, including the expression of neoantigens, thereby rendering MSI-high (MSI-H) tumors potentially more responsive to immunotherapy^22^. Clinical trials have evaluated pembrolizumab effectiveness through an agnostic approach, specifically in patients with advanced progressive solid tumors exhibiting MSI-H/dMMR^23,24^. However, the occurrence of MSI-H/MMR deficiency is mostly restricted to colorectal or endometrial cancers.

Tumor mutational burden (TMB) was recently approved by the Food and Drug Administration as a pantumor biomarker for pembrolizumab response in advanced solid cancer^25^. This approval was based on the phase 2 KEYNOTE-158 study results showing that patients with advanced solid tumors and high TMB had better ORRs to pembrolizumab than those with low TMB^26^.

SCCs can be segregated from other cancers based on their shared molecular features^27^. SCCs harbor common genomic alterations, such as somatic mutations in *TP53*, regardless of the initial location of the primary tumor^28–31^. Importantly, SCCs also present common epigenetic patterns^32,33^, which is of crucial importance considering that epigenetic modulation plays a major role in tumor escape from immunosurveillance and confers resistance to ICIs. Hence, priming the antitumoral immune response by means of modulation of the epigenome constitutes an innovative and promising approach in SCC cancer research to counteract ICI resistance^34–36^. Vorinostat is an epidrug that inhibits a large spectrum of histone deacetylases (HDACs)^37^. Various preclinical studies have reported the synergistic effect of vorinostat and ICIs in overcoming tumor immune resistance^38,39^. In 2006, vorinostat became the first HDAC inhibitor approved for the treatment of refractory cutaneous T cell lymphoma^40^.

Here, we present the efficacy and safety results of the phase 2 PEVOsq basket trial investigating the combination of the ICI inhibitor pembrolizumab with the epidrug vorinostat in patients with recurrent and/or metastatic SCC of various primary tumor locations. Our results also highlight new genomic biomarkers associated with the response to treatment.

## Results

### Participants

**Fig. 1:**
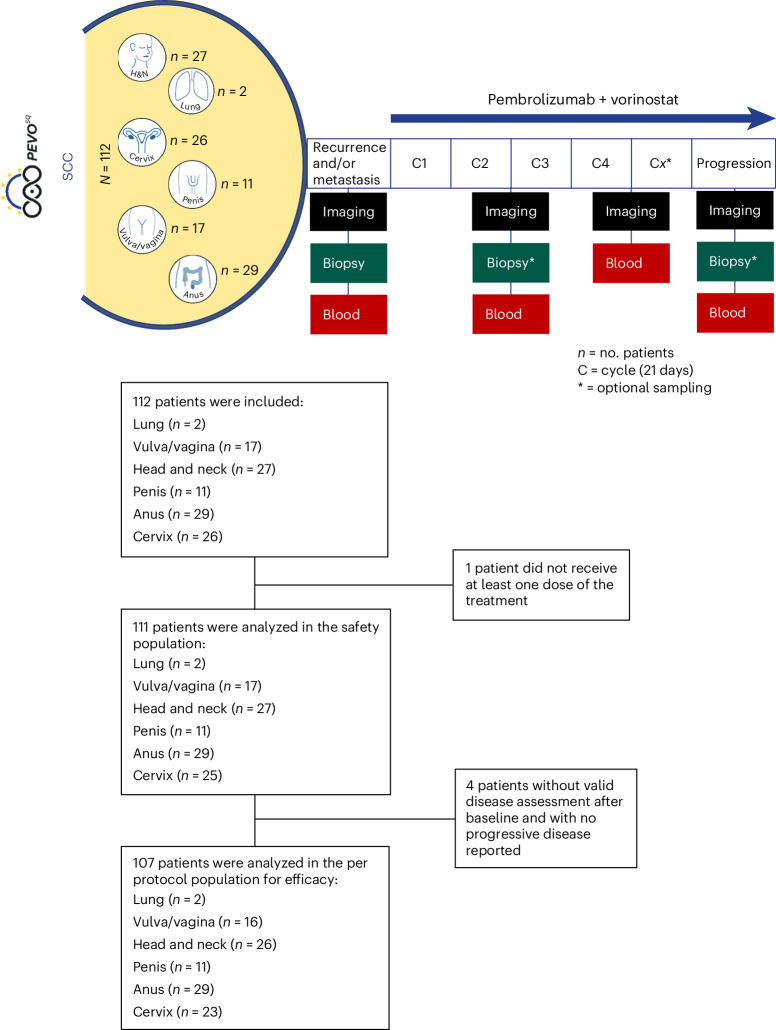
Trial design and CONSORT diagram. Images source: Unicancer library; H&N, head and neck.

Between 30 October 2020 and 10 May 2022, 112 ICI-naive patients with recurrent and/or metastatic SCC from various locations were recruited, including 29 participants with anal cancer, 27 participants with HNSCC, 26 participants with cervical cancer, 17 participants with vulvar/vaginal cancer, 11 participants with penile cancer and 2 participants with lung cancer (Fig. 1 and Supplementary Table 1). As of 14 November 2022 (cutoff date), 111 patients received at least one dose of treatment, and 107 treated patients had at least one valid disease assessment after baseline or progressed before a Response Evaluation Criteria in Solid Tumors (RECIST) disease assessment. Antitumoral activity was evaluated in 107 treated patients (4 patients did not have a valid disease assessment after baseline with no progression reported).

Baseline characteristics of the whole patient population are summarized in Table 1. Most (63%) participants were female, with a median age of 61 (range: 18–85 years), and 55% had an Eastern Cooperative Oncology Group (ECOG) performance status score of 1. The median number of prior systemic therapy lines was 1 (range: 0–4). For 53% of individuals with vulvar/vaginal SCC, the investigated regimen was the first line of treatment, whereas only 17% of participants with anal cancer did not receive any treatment in the recurrent/metastatic setting. Eighty-six (77.5%) patients had metastatic disease, whereas 25 (22.5%) patients had loco-regional recurrence. Sixty-three (57%) patients had an HPV-positive tumor with an HPV16 subtype for most of them (54 (49%) of 111 patients). The Combined Positive Score (CPS) for PD-L1 status was assessed in 102 (92%) patients. Most patients (84 (82%)) had a CPS of ≥1, and 29 (28%) had a CPS of ≥20. Fifty-nine patients (58%) presented with a tumor with a Tumor Proportion Score (TPS) of ≥1. TMB was evaluated in 80 (72%) patients, and 12 (15%) presented with high TMB. Three (4%) of the 80 patients evaluated had an MSI-H tumor (Supplementary Table 1).

**Table 1:** Patient baseline characteristics

	Lung (*n* = 2)	HNSCC (*n* = 27)	Anus (*n* = 29)	Vulva/vagina (*n* = 17)	Penis (*n* = 11)	Cervix (*n* = 25)	Total (*n* = 111)
Age, years							
Median	69	59	63	63	71	52	61
Range	(68–70)	(18–77)	(49–84)	(40–85)	(39–82)	(31–75)	(18–85)
Sex, *n* (%)							
Male	2 (100%)	22 (82%)	6 (21%)	0	11 (100%)	0	41 (37%)
Female	0	5 (18%)	23 (79%)	17 (100%)	0	25 (100%)	70 (63%)
ECOG, *n* (%)							
0	1 (50%)	12 (44%)	14 (48%)	5 (29%)	4 (36%)	14 (56%)	50 (45%)
1	1 (50%)	15 (56%)	15 (52%)	12 (71%)	7 (64%)	11 (44%)	61 (55%)
p16 status, *n* (%)							
Negative	0	17 (90%)	0	3 (25%)	4 (50%)	0	24 (30%)
Positive	0	2 (10%)	22 (100%)	9 (75%)	4 (50%)	19 (100%)	56 (70%)
Missing	2	8	7	5	3	6	31
HPV PCR typing, *n* (%)							
HPV^–^	2 (100 %)	25 (93%)	1 (3%)	8 (47%)	7 (64%)	4 (17%)	47 (43%)
HPV16	0	2 (7%)	27 (93%)	7 (41%)	4 (36%)	14 (58%)	54 (49%)
HPV18	0	0	0	0	0	2 (8%)	2 (2%)
HPV31	0	0	1 (3%)	0	0	0	1 (1%)
HPV33	0	0	0	1 (6%)	0	0	1 (1%)
HPV35	0	0	0	0	0	1 (4%)	1 (1%)
HPV59	0	0	0	0	0	2 (8%)	2 (2%)
HPV73	0	0	0	1 (6%)	0	1 (4%)	2 (2%)
Missing	0	0	0	0	0	1	1
TMB, *n* (%)							
High	1 (50%)	2 (12%)	3 (12%)	3 (23%)	1 (17%)	2 (11%)	12 (15%)
Low	1 (50%)	14 (88%)	22 (88%)	10 (77%)	5 (83%)	16 (89%)	68 (85%)
Missing	0	11	4	4	5	7	31
MSI status, *n* (%)							
MSI	0	1 (6%)	1 (4%)	1 (8%)	0	0	3 (4%)
MSS	2 (100%)	15 (94%)	24 (96%)	12 (92%)	6 (100%)	18 (100%)	77 (96%)
Missing	0	11	4	4	5	7	31
PD-L1 CPS, *n* (%)							
0	1 (50%)	5 (18%)	5 (19%)	3 (19%)	2 (22%)	2 (9%)	18 (18%)
1–19	1 (50%)	14 (52%)	13 (50%)	9 (56%)	2 (22%)	16 (73%)	55 (54%)
≥20	0	8 (30%)	8 (31%)	4 (25%)	5 (56%)	4 (18%)	29 (28%)
Missing	0	0	3	1	2	3	9
PD-L1 TPS, *n* (%)							
0%	2 (100%)	9 (35%)	11 (42%)	6 (38%)	2 (22%)	12 (54%)	42 (42%)
1–49%	0	13 (50%)	13 (50%)	8 (50%)	3 (33%)	9 (41%)	46 (46%)
≥50%	0	4 (15%)	2 (8%)	2 (12%)	4 (44%)	1 (4%)	13 (13%)
Missing	0	1	3	1	2	3	10
No. prior lines of systemic therapy							
Median	1	0	2	0	1	1	1
Range	(1–1)	(0–1)	(0–4)	(0–1)	(0–2)	(0–3)	(0–4)

Abbreviation: MSS, microsatellite stable.

### Antitumoral activity

**Fig. 2:**
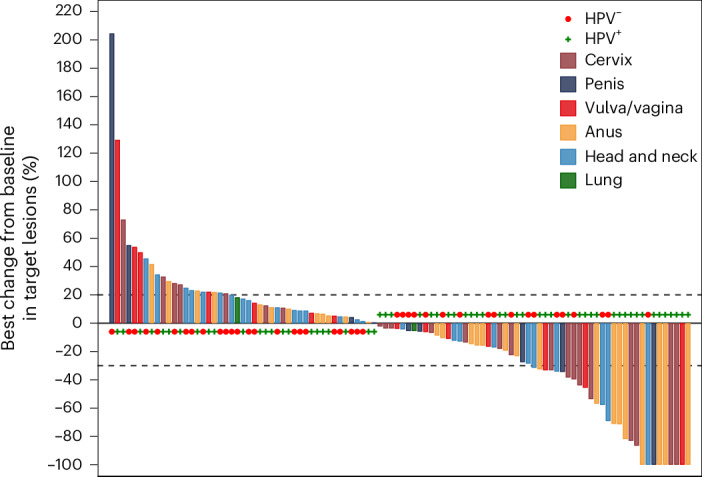
Waterfall plot based on the best target lesion reduction in the per-protocol population. *N* = 102 patients, 5 patients were excluded from the *N* = 107 per-protocol population due to no measurable lesions to calculate the best change in target lesion. Source data

The median follow-up of the per-protocol population (107 patients) was 16.6 months (95% confidence interval (CI): 15.4–19.8). In this population, the ORR was 26% (95% CI: 18–36), including 7 (6.5%) complete responses (CRs), 21 (20%) partial responses (PRs) and 44 (41%) patients with stable disease (Table 2 and Fig. 2). The study primary endpoint was met in three cohorts, including the cervical cancer cohort (ORR = 39%; 95% CI: 20–62), anal cancer cohort (ORR = 31%; 95% CI: 15–51) and vulvar/vaginal cancer cohort (ORR = 19%; 95% CI: 4.0–46; Table 2). By contrast, the primary objective was not reached in the HNSCC and penile cancer cohorts, with ORRs of 19% (95% CI: 6.6–39) and 18% (95% CI: 2.3–52), respectively, according to the prespecified statistical hypotheses and decision rules (Supplementary Table 2).

**Table 2:** ORR in the efficacy population (*N* = 107) and according to primary tumor location

	Lung (*n* = 2)	HNSCC (*n* = 26)	Anus (*n* = 29)	Vulva/vagina (*n* = 16)	Penis (*n* = 11)	Cervix (*n* = 23)	Total (*n* = 107)
RECIST responses, *n* (%)							
CR	0	0	4 (14%)	1 (6.5%)	0	2 (9.0%)	7 (6.5%)
PR	0	5 (19%)	5 (17%)	2 (12%)	2 (18%)	7 (30%)	21 (20%)
SD	1 (50%)	13 (50%)	14 (48%)	4 (25%)	4 (36%)	8 (35%)	44 (41%)
PD	1 (50%)	8 (31%)	5 (17%)	8 (50%)	3 (27%)	6 (26%)	31 (29%)
Nonevaluable^a^	0	0	1 (4.0%)	1 (6.5%)	2 (18%)	0	4 (3.5%)
ORR	0%	19%	31%	19%	18%	39%	26%
95% CI (two-sided)		(6.6–39)	(15–51)	(4.0–46)	(2.3–52)	(20–62)	(18–36)
95% CI (one-sided)		(7.9–100)	(17–100)	(5.3–100)	(4.9–100)^b^	(22–100)	
DOR							
Median DOR, months	NA	3.2	NR	3.0	1.1	15.2	9.7
95% CI		(1.3–NR)	(5.6–NR)	(2.3–NR)	(1.1–NR)	(1.4–NR)	(3.1–15)

Abbreviations: NA, not available; NR, not reached; PD, progressive disease; SD, stable disease.

^a^ Four patients without valid disease assessment after baseline and with progressive disease before postbaseline disease assessment.

^b^ 90% CI (one-sided).

In univariable analysis, age was the only clinical parameter associated with ORR, with older patients (≥60 years) showing higher response rates than younger patients (36% versus 15%, *P* = 0.01; Supplementary Table 3).

**Fig. 3:**
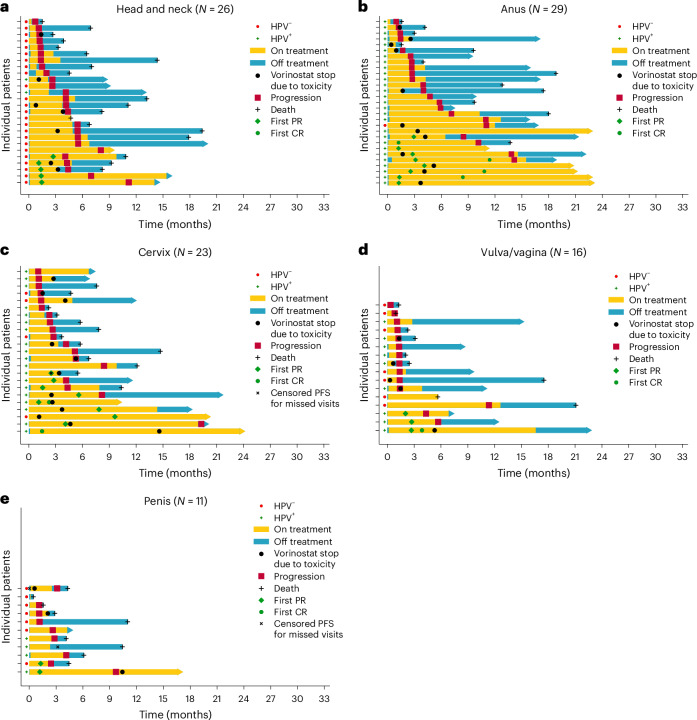
Swimmer plots showing the antitumor activity of pembrolizumab plus vorinostat by cancer type. **a**, Head and neck cancer (*N* = 26 patients). **b**, Anal cancer (*N* = 29 patients). **c**, Cervical cancer (*N* = 23 patients). **d**, Vulvar/vaginal cancer (*N* = 16 patients). **e**, Penile cancer (*N* = 11 patients). No statistical test was used. Source data

In the per-protocol population, the overall median duration of response (DOR) was 9.7 months (95% CI: 3.1–15.2), with medians ranging from 1.1 months in the penile cancer cohort to 15.2 months in the cervical cancer cohort, and a median DOR was not reached in the anal cancer cohort (Table 2). Figure 3a–e depicts swimmer plots of each SCC cohort evaluated.

**Extended Data Fig. 1:**
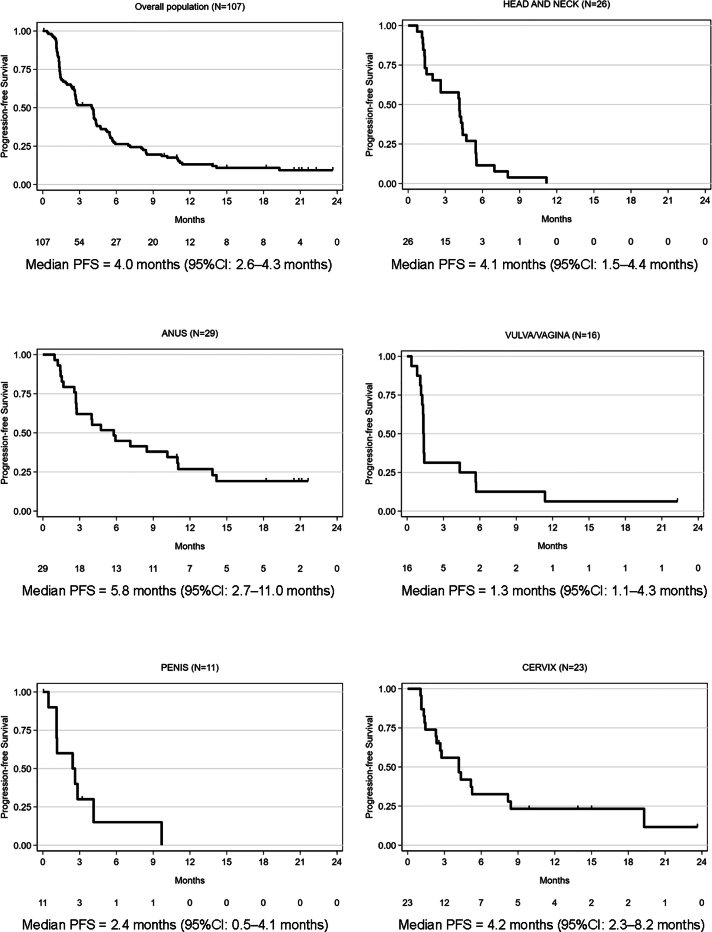
Progression-free survival (PFS) in the per-protocol population (n = 107) and according to tumor type. Median PFS are provided below each graph. N = 107 patients comprising N = 26 Head and neck cancers, N = 29 anus, N = 16 vulva/vagina, N = 11 penis, N = 23 cervix. No statistical test was performed. Source data

**Extended Data Fig. 2:**
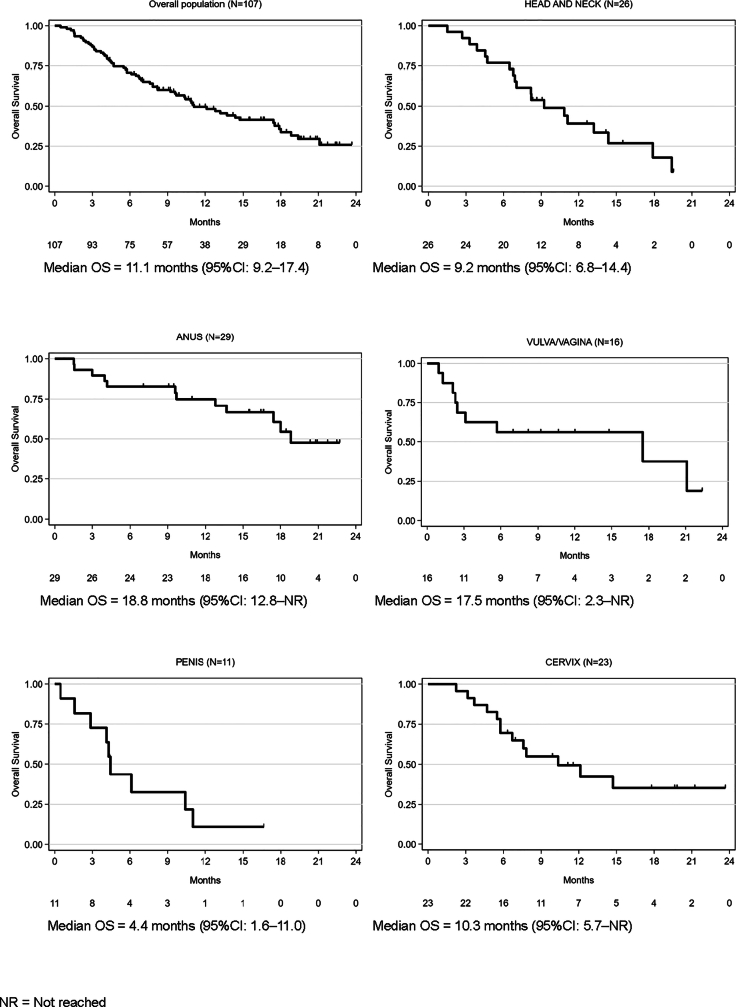
Overall survival in the per-protocol population (n = 107) and according to tumor type. Median OS are provided below each graph. N = 107 patients comprising N = 26 Head and neck cancers, N = 29 anus, N = 16 vulva/vagina, N = 11 penis, N = 23 cervix. No statistical test was performed. Source data

The overall median progression-free survival (PFS) was 4.0 months (95% CI: 2.6–4.3), with medians varying from 1.3 months in the vulvar/vaginal cancer cohort to 5.8 months in the anal cancer cohort (Extended Data Fig. 1). The median overall survival (OS) was 11.1 months (95% CI: 9.2–17.4), with medians ranging from 4.4 months in the penile cancer cohort to 18.8 months in the anal cancer cohort (Extended Data Fig. 2).

### Safety

In the safety population, 101 (91%) of 111 patients developed at least one treatment-related adverse event (TRAE). The most frequent TRAEs occurring in more than 10% of the patients were asthenia (61%), nausea (51%), diarrhea (37%), decreased appetite (37%), vomiting (26%) and investigation disorders, including hematotoxicity (anemia in 41% and thrombocytopenia in 36% of cases) and creatine increase (38%; Table 3 and Supplementary Table 4). These TRAEs were mainly related to vorinostat (Supplementary Table 5), with pembrolizumab-related TRAEs being less frequent (<10%), including hypothyroidism (9%), dry skin and pruritus (4.5% each), followed by diarrhea, arthralgia and asthenia (3.6% each; Supplementary Table 6). Most frequent grade 3 or 4 TRAEs were grade 3 asthenia (8%), diarrhea (6%), decreased appetite (5%), nausea (3%) and investigation disorders, including anemia (9%), thrombocytopenia (5%) and creatinine increase (5%), with grade 4 anemia, thrombocytopenia and creatinine increase (2% each). A summary of grade 3 and 4 TRAEs and details of the TRAE outcomes is available in Supplementary Table 7. Forty-eight (43%) patients experienced at least one serious adverse event (SAE), and 22 (20%) patients experienced at least one treatment-related SAE (Supplementary Table 8). The most frequent treatment-related SAEs were acute kidney injury in five (5%) patients and anemia in three patients (3%), followed by diarrhea, thrombocytopenia and adrenal insufficiency in two patients each (1.8%).

**Table 3:** Summary of treatment-related SAEs occurring in >10% of patients

TRAEs, *n* (%)	Grade 1	Grade 2	Grade 3	Grade 4	All (*N* = 111)
**Gastrointestinal disorders**					
Diarrhea	19 (17%)	15 (14%)	7 (6%)	0	41 (37%)
Nausea	19 (17%)	34 (31%)	3 (3%)	0	56 (51%)
Vomiting	16 (14%)	12 (11%)	1 (1%)	0	29 (26%)
**General disorders**					
Asthenia	21 (19%)	38 (34%)	9 (8%)	0	68 (61%)
**Blood and lymphatic system disorders**					
Anemia	15 (14%)	18 (16%)	10 (9%)	2 (2%)	45 (41%)
Lymphopenia	3 (3%)	8 (7%)	2 (2%)	0	13 (12%)
Thrombocytopenia	17 (15%)	14 (13%)	6 (5%)	3 (3%)	40 (36%)
**Metabolism and nutrition disorders**					
Weight decrease	6 (5%)	7 (6%)	0	0	13 (12%)
Decreased appetite	14 (13%)	22 (20%)	5 (5%)	0	41 (37%)
**Renal and urinary disorders**					
Creatinine increase	16 (14%)	31 (28%)	6 (5%)	2 (2%)	55 (50%)
**Skin and subcutaneous tissue disorders**					
Alopecia	8 (7%)	4 (4%)	0	0	12 (11%)
Dry skin	10 (9%)	2 (2%)	0	0	12 (11%)
**Musculoskeletal and connective tissue disorders**					
Muscle spasms	8 (7%)	3 (3%)	0	0	11 (10%)
**Nervous system disorders**					
Dysgeusia	5 (5%)	6 (5%)	1 (1%)	0	12 (11%)

Overall, 83 (75%) patients developed a TRAE leading to treatment modifications (that is, dose reduction or delay/interruption). Most patients had to interrupt and/or modify their treatment at least once due to vorinostat-related toxicities (73 patients (66%)). Nine (9%) patients permanently discontinued pembrolizumab due to an adverse event; most were grade 2, and two were grade 4. Twenty-four (22%) patients had delayed pembrolizumab prescription due to toxicity (Supplementary Table 9a). Forty-two (39%) patients permanently discontinued vorinostat, mainly due to grade 2 or 3 adverse events (Supplementary Table 9b). No toxic death was established during the study. There were six grade 5 SAEs reported during the study. Specifically, two deaths were related to an underlying disease (diarrhea, hemodynamic shock), one death was related to an infection and underlying disease (acute renal failure), and one death was related to stent thrombosis leading to a lower left limb ischemia. For the two remaining patients, the cause was unknown (the patients were found dead at home, and causality could not be established).

### Molecular characteristics and clinical outcome correlation

In univariable analysis, the ORR was associated with HPV status, with patients with HPV-positive SCC showing higher ORRs than patients with HPV-negative SCC (34% versus 16%, *P* = 0.03; Supplementary Table 10).

Evaluation of PD-L1 status revealed a positive association between CPS and ORR, with a better ORR linked to higher CPS scores (ORR of 5.6% for CPS = 0 versus 30% for CPS ≥ 1, *P* = 0.04) and reaching up to 45% in patients with a CPS of ≥20. High TMB was also positively associated with ORR in univariable analysis (58% for high TMB versus 20% for low TMB, *P* = 0.01). By contrast, no significant association was observed between ORR and TPS.

**Extended Data Fig. 3:**
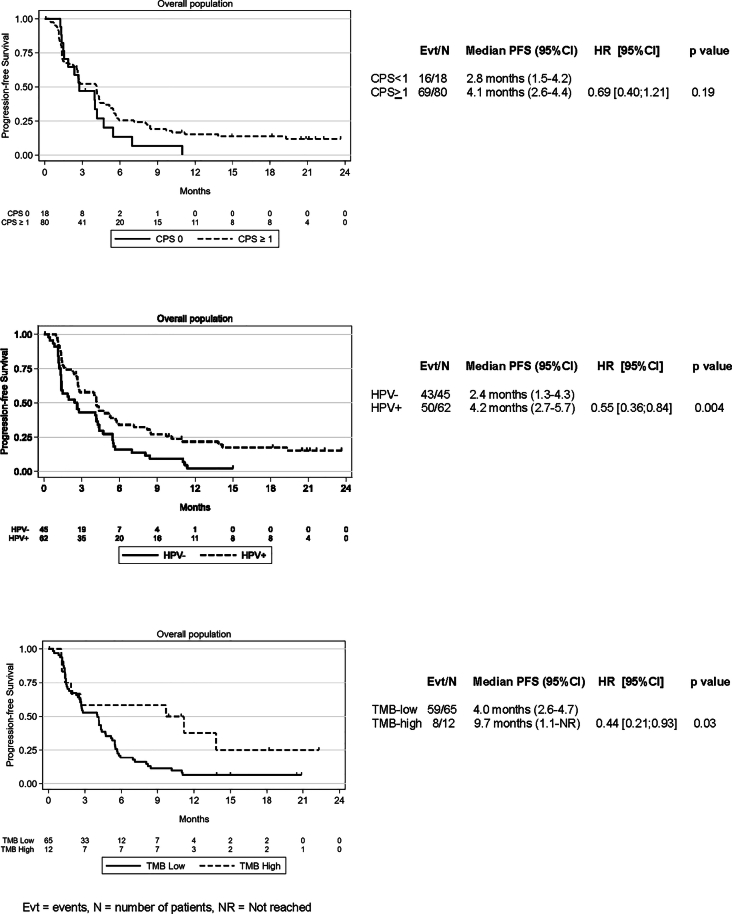
Progression-free survival (PFS) according to biomarkers in the per-protocol population. N = 107 patients are included among which N = 98 with available CPS evaluation, N = 107 with HPV status and N = 77 patients with available TMB status. P-values from Log-rank test are reported. All statistical tests were two-sided. No adjustments were made for multiple comparisons. Median PFS for each population according to biomarkers are reported on the right of each graph. Source data

**Extended Data Fig. 4:**
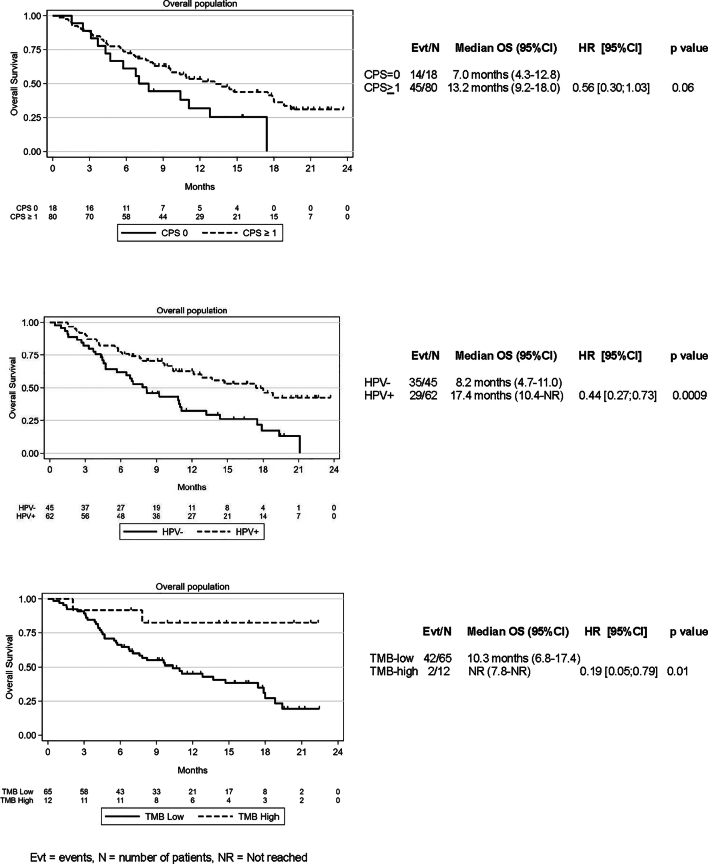
Overall survival according to biomarkers in the per-protocol population. N = 107 patients are included among which N = 98 with available CPS evaluation, N = 107 with HPV status and N = 77 patients with available TMB status. P-values from Log-rank test are reported. All statistical tests were two-sided. No adjustments were made for multiple comparisons. Median PFS for each population according to biomarkers are reported on the right of each graph. Source data

In line with our observation for ORR, high TMB was associated with lower risk of death (OS), progression or death (PFS; OS hazard ratio (HR): 0.19, 95% CI: 0.05–0.79, *P* = 0.01; PFS HR: 0.44, 95% CI: 0.21–0.93, *P* = 0.03) and HPV positivity (OS HR: 0.44, 95% CI: 0.27–0.73, *P* < 0.001; PFS HR: 0.55, 95% CI: 0.36–0.84, *P* < 0.05). However, PFS and OS were prolonged in the CPS ≥ 1 subgroup, although statistical significance was not reached (*P* = 0.19 and *P* = 0.06, respectively; Extended Data Figs. 3 and 4).

### Genomic biomarkers of response to treatment

To determine the genomic alterations and mutations driving SCCs, we processed 77 samples for whole-exome sequencing (WES) analyses. We identified somatic single-nucleotide variants (SNVs) and copy number variants (CNVs), which were further filtered to determine the main driver alterations. In line with previous studies^29^, genomic analyses revealed somatic mutations and alterations, with *PIK3CA* (31%) and *CCND1* (14%) as the most frequently altered oncogenes across all samples. *TP53*, *KMT2D* and *KMT2C* were among the most frequently mutated tumor suppressor genes.

**Fig. 4:**
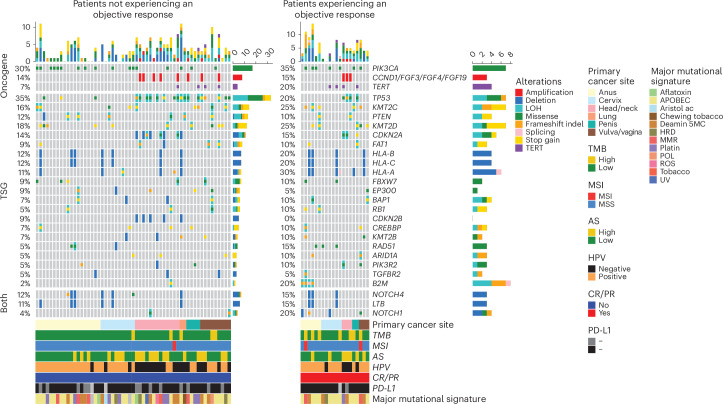
Oncoprint of druggable alterations in 77 patients with available WES data experiencing an objective response versus others. Frequently altered genes that were altered in at least 5% of the samples were grouped according to CR/PR status. Clinical features, TMB, MSI, aneuploidy score (AS), major mutational signatures and somatic alterations are indicated in the legend. The bar chart on the top indicates the composition of alterations per sample, while the bar chart on the right indicates the composition of alterations per gene of interest. To compare alteration rates between patients experiencing an objective response and patients not experiencing an objective response, a Fisher’s exact test was performed. The resulting *P* values were adjusted using the Benjamini–Hochberg procedure. Results of the statistical tests are provided in Supplementary Tables 11 and 12; indel, insertion/deletion; LOH, loss of heterozygosity; MSS, microsatellite stable; ROS, reactive oxygen species. Aristol ac, aristolochic acid exposure; Deamin 5MC, deamination of 5-methylcytosine; HRD, homologous recombination deficiency; Platin, prior chemotherapy treatment with platinum drugs; POL, polymerase epsilon exonuclease domain mutations; TSG, tumor suppressor genes. Source data

Although not statistically significant, we did observe a tendency of higher frequency of alteration in *B2M* in patients with an objective response (20%) compared to patients not experiencing an objective response (2%; *P* < 0.05, adjusted *P* = 0.39; Fig. 4). Interestingly, we found that patients with alterations in *RAD51*, *NOTCH1* or *B2M* showed longer PFS (*P* < 0.05; Supplementary Table 11). Patients with alterations in *PIK3CA* showed longer OS (*P* < 0.05), whereas alterations in *TP53* were associated with worse OS (*P* < 0.05; Supplementary Table 12).

**Fig. 5:**
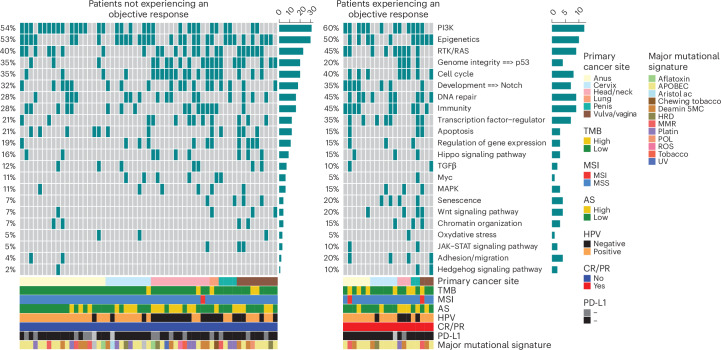
Main altered pathways in 77 patients with available WES data experiencing an objective response versus others. Frequently altered genes are grouped according to curated signaling pathways detailed in Supplementary Table 2. Clinical features, TMB, MSI, aneuploidy score and major mutational signatures are indicated in the legend. The bar chart on the top indicates the composition of alterations per sample, and the bar chart on the right indicates the composition of alterations per gene of interest. To compare mutational pathways between patients experiencing an objective response and patients not experiencing an objective response, a Fisher’s exact test was performed. The resulting *P* values were adjusted using the Benjamini–Hochberg procedure. Results of the statistical tests are provided in Supplementary Tables 13 and 14. Source data

Mutational pathway-based analyses and mutational signatures analyses based on COSMIC signatures did not show any statistically significant association with ORR (Fig. 5). However, patients with no alterations in the regulation of gene expression pathways showed longer PFS (*P* < 0.05; Supplementary Table 13). Furthermore, we found that patients with no alterations in the Myc and genome integrity (*TP53*) pathways showed prolonged OS (*P* < 0.05; Supplementary Table 14).

## Discussion

PEVOsq is a trial evaluating an innovative treatment strategy across SCCs of various origins in Europe. In our study, the combination of pembrolizumab and vorinostat demonstrated significant antitumor activity in patients with recurrent and/or metastatic SCC, and the toxicity elicited by this regimen was manageable. Our comprehensive genomic analysis allowed us to identify potential biomarkers that might be predictive of the efficacy of the combination.

The combination of pembrolizumab and vorinostat was most effective in the anal and cervical cancer cohorts, in which the primary endpoint was met. Published data from phase 1 or 2 studies assessing either nivolumab^13^ or pembrolizumab^14–16^ in previously treated advanced anal SCC reported ORRs ranging from 11% to 24%. Notably, the KEYNOTE-028 phase 1b study, which exclusively enrolled patients with PD-L1-positive (≥1% of tumor and/or immune cells) advanced anal SCC, reported an ORR of 17%^14^. In our trial, individuals with recurrent and/or metastatic anal SCC who had received a median of two prior lines of treatment without any selection based on PD-L1 expression showed an ORR of 31% (95% CI: 15–51) and median PFS and OS values of 5.8 and 18.8 months, respectively, in response to the combination of pembrolizumab and vorinostat. This represents a substantial improvement compared to the advanced anal cancer cohort investigated in the phase 2 KEYNOTE-158 trial, in which pembrolizumab monotherapy led to an ORR of 11% along with median PFS and OS values of 2.0 and 11.9 months, respectively^16^. Regarding our cervical cancer cohort, patients mainly received the pembrolizumab plus vorinostat combination as second-line treatment. In this cohort, an ORR of 39% was obtained, along with median PFS and OS values of 4.2 and 10.3 months, respectively. These results compared favorably with a prior phase 2 trial assessing the efficacy of pembrolizumab alone in individuals with cervical cancer that were previously treated, in which all responses were seen in patients with PD-L1-positive tumors (CPS ≥ 1)^41^. In these patients, an ORR of 12%, a median PFS of 2.1 months and a median OS of 9.4 months were recorded. Notably, pembrolizumab plus platinum-based chemotherapy and bevacizumab have recently emerged as new standards of care for the first-line treatment of patients with persistent, recurrent or metastatic cervical cancer presenting with PD-L1 (CPS ≥ 1) expression^12^.

Vulvar and vaginal SCC are considered rare as they represent just 4% of all gynecologic cancers. No standard of care exists for these patients in a recurrent and/or metastatic setting. The combination of pembrolizumab with vorinostat resulted in encouraging antitumor efficacy in this rare population, with an ORR of 19%, a median PFS of 1.3 months and a median OS of 17.5 months. However, putting these results in perspective is challenging as few data were reported using anti-PD-1 therapy as a single agent in this population^17,18^. Nonetheless, the CheckMate 358 trial assessing nivolumab monotherapy, conducted on just five individuals with vulvar or vaginal SCC, showed a PR in one patient (ORR of 20%). Unfortunately, due to the small number of patients, PFS and OS median values were not available^18^. Of note, in our study, 53% of vulvar/vaginal cancers were HPV related, which might not reflect the real-world population (25% of vulvar cancers and 78% of vaginal cancers are attributable to HPV)^3^.

In the penile cancer cohort, the efficacy of the investigated combination appeared to be limited, with an ORR of 18% in our trial. A retrospective study from the Global Society of Rare Genitourinary Tumors reported an ORR of 13% with anti-PD-1 therapy as a single agent administered mainly in the second-line setting for advanced penile SCC, regardless of PD-L1 status^21^.

Finally, in our HNSCC cohort, the observed antitumor activity was limited and did not meet its primary endpoint, with an ORR of 19%. This efficacy is in line with the ORR of 17% reported across a similar overall population of unselected patients for PD-L1 status included in the KEYNOTE-048 trial investigating pembrolizumab monotherapy as first-line treatment for recurrent/metastatic HNSCC^11^. On the other hand, a phase 2 trial assessing the efficacy of vorinostat and pembrolizumab in advanced head and neck cancers reported an ORR of 32%^29^. However, a notable difference resides in the inclusion of patients with nasopharyngeal carcinomas (16%) and skin cutaneous primary SCCs (8%), who might exhibit higher response rates to ICIs than individuals with HNSCC. Moreover, 52% of oropharyngeal tumors were p16^+^ compared to just 10% in our study. Collectively, these disparities may account for the discrepancies in ORRs between these two trials.

In subgroup analyses, the only clinical parameter that was associated with response rate was age, with older patients (≥60 years) showing higher response rates than younger patients. Published data related to age and response to ICIs are conflicting. In a large meta-analysis, Elias et al. found that pembrolizumab, nivolumab and atezolizumab had comparable efficacy in younger and older patients^42^. Another study showed that older patients with advanced non-small cell lung cancer (NSCLC) responded less well to nivolumab, with a significantly lower OS in patients aged >75 years than in younger patients^43^. Our group conducted a retrospective study in patients with recurrent and or metastatic HNSCC and found that an age of >70 years was associated with longer PFS but not OS, while maintaining comparable rates of adverse events^44^. We must acknowledge that findings in the PEVOsq study regarding a potential correlation between age and efficacy come from a subgroup analysis that should be further validated.

Regarding safety, the toxicity elicited by the combination of pembrolizumab plus vorinostat was manageable but still substantial, with grade ≥3 toxicities experienced by 44.4% of patients. This is slightly above the 36% of grade ≥3 TRAEs reported in a previous study combining vorinostat and pembrolizumab in HNSCC^45^, which is higher than the reported TRAEs of high-grade with administration of pembrolizumab alone in this population (13% in KEYNOTE-040 and 17% in KEYNOTE-048)^10,11^. In a randomized phase 2 trial comparing first-line pembrolizumab plus vorinostat versus pembrolizumab alone in patients with metastatic NSCLC, the most common TRAEs in the combination arm included nausea (44%), fatigue (41%), diarrhea (35%) and increased creatinine (33%), and 49% of patients required a dose reduction of vorinostat, most commonly due to grade 2/grade 3 fatigue or nausea^46^. Vorinostat dosage had to be adjusted in a considerable proportion of patients in our study, with 66% of them subjected to at least one treatment interruption and/or dose reduction due to toxicity, including hematotoxicity, gastrointestinal toxicity, asthenia and creatinine increase. These TRAEs were more frequent in our study likely due to a greater heterogeneity in our study population, who may also be frailer than treatment-naive patients with metastatic NSCLC.

PD-L1 status was the first validated biomarker of efficacy to identify patients most likely to derive a benefit from pembrolizumab either as a stand-alone agent^16^ or in combination with chemotherapy, for example, in advanced HNSCC and cervical cancer^11,12^. Accordingly, we observed that PD-L1 positivity was associated with a better ORR. However, no significant association was found between ORR and TPS. Such a result was expected as TPS is thought to be less sensitive than CPS at a low cutoff (that is, CPS ≥ 1) for defining PD-L1 expression status in HNSCC^47^.

HPV status is inconsistently reported to impact the efficacy of ICIs^48^. In the overall population, patients with HPV-related tumors derived greater benefits from pembrolizumab combined with vorinostat than patients with HPV-negative tumors. Previous in vitro data using a model of primary human keratinocytes suggested that vorinostat might have a direct effect on HPV transmission and might trigger apoptosis in HPV-infected cells, whereas uninfected tissues were spared, suggesting that vorinostat might have activity on HPV-related cancer^49^. Of note, a confounding factor could be that patients with HPV-positive HNSCC are known to have a better outcome than their HPV-negative counterparts, and further randomized data will be needed^50^.

Our data also indicate that high TMB was associated with better ORR than low TMB, in line with the phase 2 KEYNOTE-158 trial findings, demonstrating a positive association between high TMB and improved ORR in patients with advanced solid tumors treated with pembrolizumab^28^.

The comprehensive genomic analyses conducted here provide invaluable insights into the genetic landscape of SCCs across various primary cancer sites. Our analyses first confirm alterations in SCCs that drive pathogenesis, in line with previous studies^29^.

The observed improvement in PFS among patients with mutations in *RAD51*, *NOTCH1* or *B2M* indicates that these genetic alterations may play a critical role in delaying disease progression, even though we might lack power to find a statistically significant impact on OS.

*B2M* encodes a critical component of the major histocompatibility complex class I molecule, which is required for the presentation of tumor antigens to CD8^+^ T cells, and alterations in *B2M* have been associated with decreased *B2M* expression and acquired resistance to ICIs^51^. Surprisingly, our results suggest that *B2M*-inactived tumors could respond to ICIs. Previous data showed that, in this case, the antitumoral immune response is mediated by CD4^+^ T cells and natural killer cells, which do not involve major histocompatibility complex class I recognition^51^.

Dysregulation of DNA damage repair mechanisms, such as alterations in *RAD51*, is known to contribute to increased genomic instability. The role of *RAD51* in cancer progression is supported by its involvement in homologous recombination repair. Impairment in homologous recombination repair can result in accumulated mutations that drive tumor heterogeneity. This genomic instability may also render tumor cells more susceptible to ICIs^52^.

*NOTCH1* mutations, recognized as prognostic biomarkers in various cancers, including HNSCC, are known to influence cell differentiation, proliferation and apoptosis^53^. Our analyses showed that combination therapy of pembrolizumab and vorinostat significantly improved PFS in patients harboring *NOTCH1* mutations. This outcome is consistent with findings that *NOTCH1* alterations may sensitize tumors to epigenetic modulation and immune-based therapies, making combination strategies particularly effective^54^.

Improved OS in patients with alterations in *PIK3CA* is consistent with previous studies that have identified this gene as a biomarker associated with favorable responses to pembrolizumab. Although *PIK3CA* mutations can drive oncogenesis, inhibition of the PI3K pathway is associated with increased sensitivity to immunotherapies^55^.

Patients with *TP53* mutations showed worse OS (*P* < 0.05), which is typical for most cancers, including SCCs^56^. As a tumor suppressor gene involved in multiple regulatory processes, *TP53* mutations lead to genomic instability, faster disease progression and poor prognosis.

In addition, patients showing no alterations in the Myc pathway and genomic integrity pathway showed longer OS (*P* < 0.05). Dysregulation of the Myc pathway is a common feature in many cancers, leading to increased tumor aggressiveness, resistance to therapy and poor clinical outcomes^57^. The absence of alterations in this pathway may indicate a more favorable tumor biology, allowing for more effective therapeutic responses.

Finally, the integrity of genome maintenance mechanisms is critical for preventing the accumulation of mutations that can lead to genomic instability, a hallmark of cancer progression. Genome integrity (p53) pathways are often disrupted in cancer and may lead to cancer evolution and resistance to treatments^58^.

Limitations of the PEVOsq trial are linked to its basket design relying on several small cohorts of different tumor types, thereby limiting the power of the conclusions drawn and warranting caution in overinterpreting these results. Especially for the vulvar/vaginal and penile cancer cohorts, the number of patients included was small. Given the rarity of these cancers, their inclusion in clinical trials remains a challenge. Nonetheless, as all the included patients presented with SCC, the tumors shared molecular similarities and immunologic features that separate them from other cancers, independent of their organ of origin^27^. In addition, the effect of an epidrug is by essence not restricted to a single tumor type and therefore is expected to affect all the cohorts studied^59^. Another limitation rests with the absence of designated control arms, a decision made to streamline the trial execution. Finally, due to the low number of patients evaluated per cohort, the predictive value of the biomarkers identified is limited by the univariate nature of the analyses.

The multicenter PEVOsq basket trial presents the advantage of including rare cancers such as vulvar/vaginal and penile cancers, for which specific trials are problematic to set up due to recruitment difficulties. In this study, we could identify which kinds of patients with recurrent and/or metastatic SCC were most likely to respond to the combination of pembrolizumab and vorinostat, especially participants with anal cancer, cervical cancer or vulvar/vaginal cancer, and we could highlight potential biomarkers for response to the combination treatment. For these patients, epidrug with immunotherapy combinations should be considered for further exploration in larger clinical trials.

In conclusion, the combination of pembrolizumab and vorinostat showed promising antitumor activity in patients with recurrent and/or metastatic SCC and more specifically in patients with anal, cervical or vulvar/vaginal cancer, along with an overall manageable safety profile. Nonetheless, dosage of vorinostat had to be reduced in a substantial proportion of patients. The predictive biomarkers identified consist of PD-L1 positivity, HPV positivity and high TMB. Our comprehensive genomic analyses identified potential genomic biomarkers that may be used to better select patients who will derive a benefit from this therapeutic combination. Further studies using a larger number of patients are required to validate these findings.

## Methods

### Study design and procedures

PEVOsq is an open-label, nonrandomized, multicenter, basket phase 2 trial evaluating the antitumoral activity of pembrolizumab in combination with vorinostat in adult patients with recurrent and/or metastatic SCC of the lung, head and neck, cervix, anus, vulva/vagina and penis. As anti-PD-1 immunotherapy in combination with chemotherapy was approved as first-line treatment for NSCLCs, patient accrual to the PEVOsq lung cohort was subsequently terminated.

Pembrolizumab was administered intravenously at a dose of 200 mg every 3 weeks in combination with vorinostat given orally at 400 mg once daily with food, according to the recommended phase 2 dose determined in a previous phase 1/2 study^60^. The duration of each treatment cycle was 3 weeks. Patients were treated until disease progression (or for up to 35 cycles for pembrolizumab), unacceptable toxicity or patient decision. Pembrolizumab rechallenge was allowed at disease progression under certain conditions and after validation by the sponsor.

A fresh tumor biopsy was mandatory before treatment (baseline). Biopsies within the week before the first disease assessment and at disease progression were optional. Radiological evaluations were performed every 6 weeks. Blood samplings for research were performed at baseline, at day 1 of cycles 3 and 5 and at disease progression.

The study was approved by the Ethics Committee of the National Institute of Pharmacy and Nutrition and were performed in accordance with the Declaration of Helsinki, the Good Clinical Practice guidelines of the International Conference on Harmonization and relevant French and European laws and directives. All patients provided written informed consent before enrollment. This study was registered at EudraCT (2019-003839-33) and ClinicalTrials.org (NCT04357873). Sex was reported in the patient baseline characteristics, and no other sex/gender analysis was performed. This decision aligns with common practices in France, where such distinctions (sex, race and ethnicity) are generally not made unless there is a specific scientific question that necessitates it. French law tends to protect against making distinctions based on these categories except in cases where it is explicitly justified by the research objectives, which was not applicable in our case.

### Participants

Adult patients with histologically confirmed recurrent and/or metastatic SCC of the head and neck, cervix, vulva/vagina, penis or lung were eligible. Patients were required to have radiologically confirmed progressive recurrent and/or metastatic disease with measurable disease according to RECIST version 1.1 (RECIST1.1) and at least one lesion amenable to biopsy for study purposes (excluding bone lesions)^61^. Prior treatment for recurrent and/or metastatic disease was allowed. Additional key eligibility criteria included an ECOG performance status score of 0–1 and adequate organ function. Patients were excluded in cases of previous exposure to anti-PD-1, anti-PD-L1 or anti-PD-L2 agents or to any other stimulatory or co-inhibitory T cell receptor (for example, CTLA-4, OX40, CD137 and so on) and HDAC inhibitors. Patients were also excluded if they had central nervous system involvement that had not been controlled for at least 3 months, had ongoing or recent autoimmune disease requiring systemic immunosuppressive therapy, had undergone solid organ transplantation or had a known history of human immunodeficiency, hepatitis B or C virus infection or an active infection requiring systemic therapy.

### Objectives and endpoints

The primary objective was to evaluate the antitumor activity of pembrolizumab in combination with vorinostat in patients with recurrent and/or metastatic SCC of the head and neck, cervix, lung, anus, vulva/vagina and penis using ORR by investigator assessment as the primary endpoint. ORR was defined in each cohort as the percentage of evaluable patients for ORR, designated as the proportion of patients with a CR or a PR as best response according to RECIST1.1 (ref. ^61^). Key secondary endpoints included DOR, PFS, OS and incidence of adverse events. CONSORT guidelines were followed^62^.

DOR was evaluated in patients with either a CR or PR and defined as the time from the first CR or PR assessment to the date of the first occurrence of progressive disease or death from any cause (if death occurred within a predefined period), whichever came first.

PFS was defined according to RECIST1.1 as the time from inclusion to the date of disease progression or death from any cause, whichever came first. At the time of analysis, a patient alive and without disease progression was censored at the date of the last tumor assessment. Patients alive without disease progression who started a new anticancer therapy were censored at the date of the last tumor assessment before the start of a new anticancer therapy.

OS was defined as the time from inclusion to the time of death from any cause. Patients who were alive at last follow-up news were censored at this date.

To assess safety, adverse events were evaluated and reported according to the National Cancer Institute’s Common Terminology Criteria for Adverse Events version 5.0 in each cohort and in the overall study population.

Translational endpoints aimed to assess the link between selected biomarkers and their impact on response to treatment. These biomarkers included, but were not limited to, tumor tissue PD-L1 expression (evaluated by immunohistochemistry (IHC)), p16 and HPV status and tumor mutational load as assessed by WES and molecular signatures (such as homologous recombination deficiency and MSI).

### IHC

IHC staining was performed on 4-μm-thick sections using a PD-L1 IHC 22C3 PharmDx assay on a Dako Autostainer Link 48 (22C3-Autostainer) according to the manufacturer’s instructions^63^. The CPS was defined as the number of positive tumor cells, lymphocytes and macrophages divided by the total number of viable tumor cells, multiplied by 100 and capped at 100. The TPS was defined as the percentage of positive tumor cells.

### HPV typing

Real-time PCR using SYBR Green and specific primers for HPV16, HPV18 and HPV33 was performed on a QuantStudio 7 Flex Real-Time PCR System (Thermo Fisher Scientific). Multiplexed amplification was performed in a 26-µl volume using SYBR Green PCR Master Mix at a final concentration 1×, HPV16 primers at 0.7 µM each, HPV18 and HPV33 primers at 1 µM each (HPV16 forward: 5'-GTGGACCGGTCGATGTATGT-3' and reverse: 3'-CATGCAATGTAGGTGTATCT-5'; HPV18 forward: 5'-GCAGCACAGAAAACAGTCCA-3' and reverse: 3'-CGCCATTTGTAGTTACCTGA-5'; HPV33 forward: 5'-AGTCAAAATGGCGACACAAA-3' and reverse: 3'-ACTAATTTCCTGCAACGTAA-5'), DNA template (20 ng) and nuclease-free water. Additional PCR using GP5+/GP6+ primers (GP5 + : 5'-TTTGTTACTGTGGTAGATACTAC-3'; GP6 + : 5'-GAAAAATAAACTGTAAATCATATTC-3'), which can detect a large spectrum of HPV types, was performed on samples found negative for HPV16/HPV18/HPV33, according to previously described conditions^64^. Sanger sequencing using the GP5+ primer was performed on the PCR products to identify HPV type by comparing the obtained sequence to reference sequences.

### WES methods and analysis

Target capture and library construction were performed using a Twist Comprehensive Exome kit (Twist Biosciences) as per the manufacturer’s protocol. Briefly, 50 ng of genomic DNA was enzymatically fragmented, adaptors were added, and DNA was amplified by PCR, followed by purification. Target regions were captured with Twist Comprehensive Exome Panel probes, amplified and purified. Enriched libraries were quantified using a Qubit dsDNA High Sensitivity Assay (Thermo Fisher Scientific) and analyzed for size distribution using a Bioanalyzer 2100 (Agilent Technologies). Sequencing was performed on an Illumina NovaSeq 6000 platform using S4 Reagent Kit 300 cycles (2 × 150 paired-end reads; Illumina).

WES was performed using a DRAGEN Bio-IT Platform v4.0 (Illumina) and the human hg38 genome with matched tumor–normal samples^65^. The pipeline included highly optimized algorithms for mapping, duplicate marking and variant calling. For unmatched samples, the DRAGEN tumor-only pipeline was used, with a panel of normals (23 samples) filtering systematic noise. Resulting VCFs were annotated using SnpEff/SnpSift and databases including OncoKB, ICGC and CancerHotspots^66–68^.

Variants were filtered for oncogenes, tumor suppressor genes and dual-role genes per OncoKB. Selected SNVs included pathogenic missense mutations, frameshift insertions/deletions, stop–gain, splicing and TERT noncoding mutations. Genes were categorized into signaling pathways (Supplementary Table 15).

TMB and MSI were extracted from DRAGEN v4.0. TMB was computed with a minimum variant allele frequency of 0.05 and coverage of ≥50, classified as high (≥10) or low (<10). MSI was determined with coverage of ≥60, with scores of ≥15 classified as MSI and scores of <15 classified as microsatellite stable. Mutational signatures were identified using SigProfilerExtractor, selecting 18 major signatures per patient and considering only those contributing ≥20% of mutations^69^.

CNVs were called using Facets. Oncogene CNVs included focal amplifications (<10 megabases, copy number of ≥5). Tumor suppressor gene CNVs included deletions (copy number = 0) and loss of heterozygosity with concurrent SNV events.

### Statistics and reproducibility

The experiments were not randomized, and the investigators were not blinded to allocation during experiments and outcome assessment.

ORRs reported in the literature with pembrolizumab or nivolumab in individuals with SCC ranged from 6% to 24% depending on the primary tumor location^7,9,13,17,70^. The required number of evaluable patients for each cohort was determined using an A’Hern design based on different hypotheses^71^. To compensate for potential drop out, an additional 10% of patients in each cohort was added; therefore, a total of 112 patients was required for this study. Number of required patients, design parameters and decision rules for each cohort are summarized in Supplementary Table 2. No statistical tests based on the assumption of normality of the data distribution or equal variances were performed.

Demographic, clinical and biological characteristics were presented in the overall population and per cohort using usual statistics. Quantitative data were summarized as median, range and number of missing data. Qualitative variables were described as number, percentage and number of missing data. Between 30 October 2020 and 10 May 2022, 112 patients with recurrent and/or metastatic SCC from various locations were included, 111 patients received at least one dose of the treatment and 107 treated patients had at least one valid disease assessment after baseline or progressed before a RECIST disease assessment. Antitumoral activity was evaluated in 107 treated patients (4 patients did not have a valid disease assessment after baseline or presented with progressive disease).

Primary and secondary efficacy endpoints were assessed in the per-protocol population (*n* = 107), corresponding to all eligible patients with at least one valid postbaseline disease assessment (or with progressive disease before RECIST disease assessment) and who had received at least one dose of the study treatments. These efficacy endpoints were reported for the overall population and per cohort. ORR was presented as number, percentage and corresponding 95% CI (binomial exact distribution, two sided). One-sided CIs were also reported according to the confidence level defined for each cohort (that is, *α* level in Supplementary Table 2). Associations between baseline characteristics (clinical and biological) and ORR were assessed using *χ*^2^ or Fisher’s exact test.

Survival rates (PFS and OS) and DOR were estimated at different time points using the Kaplan–Meier method with corresponding 95% CIs. Median survival times were estimated and reported with corresponding 95% CIs. Associations between baseline characteristics (clinical and biological) and PFS and OS were assessed using a log rank test and the Cox proportional hazards model. HRs were estimated with 95% CIs.

All allocated patients who initiated treatment (at least one dose of the study treatments) were included in the safety population. Incidence of adverse events and serious adverse events were presented using frequencies and percentages by system organ class and MedDRA preferred term.

Regarding molecular analyses, 80 patients had tumor WES data, but 3 samples were removed due to low quality or not being evaluable according to the main criterion, resulting in *n* = 77 paired tumor and constitutional WES samples analyzed. Comparisons between groups for altered genes, pathways and COSMIC mutational signatures were performed using Fisher’s exact tests with a Benjamini–Hochberg correction.

### Reporting summary

Further information on research design is available in the Nature Portfolio Reporting Summary linked to this article.

## Supplementary information

Supplementary Information Study protocol NCT04357873 and CONSORT checklist.

Reporting Summary

Supplementary Table 1 Supplementary Tables 1–15.

## Source data

Source Data Figs. 2–5 and Extended Data Figs. 1–4 Best change from baseline in target lesions (%) per patient. Delays for OS, treatment, response, progressions and last news per patient. Molecular alterations detected from WES for each patient, per gene. Altered molecular pathways detected from WES for each patient. PFS delays per patient and cancer types. OS delays per patient and cancer types. PFS delays per patient and cancer types, according to biomarkers status (PD-L1 CPS, HPV and TMB). OS delays per patient and cancer types, according to biomarkers status (PD-L1 CPS, HPV and TMB).

## Data Availability

The original files and raw next-generation sequencing data generated in this study have been deposited in the EGA database under accession code EGAS50000000731. Data on EGA are under controlled access. Sequencing data will be made available upon request through EGA, and additional clinical information can be made available upon institutional approval. Requests should be addressed to N. Servant (nicolas.servant@curie.fr). The estimated timeframe for access to be granted is 2 months, and the duration will be determined according to the request needs. All relevant clinical trial data used in this study are accessible in the Source Data files and are deidentified. Source data are provided with this paper.

## Code availability

Electronic case report forms (eCRFs) were used by the clinical trials staff to collect data from patients according to the protocol. In accordance with ICH E6GCP, the sponsor monitoring team verified the eCRF entries against source data. The eCRF was developed on ENNOV CLINICAL software v8.2 solution by the Institut du Cancer de Montpellier data management team, subcontractor of the sponsor. The database is hosted by the AZ network. Data collection and analyses relied on publicly available standard bioinformatics software packages that are widely used by the community. All details about the tool versions or the different parameters applied are described in the Methods. Statistical analyses were performed using STATA v16 (StataCorp) software.

The code used for molecular analyses is available at https://github.com/bioinfo-pf-curie/PEVO. The package versions used included snpeff v5.1, snpsift v5.1, FACETS v0.6.1, SigProfiler: Extractor v1.1.21, Plotting v1.3.14, MatrixGenerator v1.2.17 and Assignment v0.031.
