# Supplementary material for: Efficacy of pembrolizumab and vorinostat combination in patients with recurrent and/or metastatic squamous cell carcinomas: a phase 2 basket trial
Source: Nat Cancer. 2025 Jun 30;6(8):1370–83. doi: 10.1038/s43018-025-01004-2 (PMC12380617; doi:10.1038/s43018-025-01004-2)
Supplement: Supplementary file 1 — Study protocol NCT04357873 and CONSORT checklist. [file 43018_2025_1004_MOESM1_ESM.pdf]

nature cancer

Supplementary information https://doi.org/10.1038/s43018-025-01004-2

Efficacy of pembrolizumab and vorinostat combination in patients with recurrent and/ or metastatic squamous cell carcinomas: a phase 2 basket trial

In the format provided by the authors and unedited

Personalized Medicine Group
unicancer

UNICANCER Group Tumor:

Personalized Medicine

Protocol n°: UC-GMP-1908

EudraCT n°: 2019-003839-33

Phase II basket trial evaluating the efficacy of a combination of pembrolizumab and vorinostat in patients with recurrent and/or metastatic squamous cell carcinoma

Abbreviated title: PEVOsq

PEVO sq

Version n°3.2 – 21st May 2021

PROTOCOL	VERSION N° - DATE	CPP approval	ANSM authorisation
**INITIAL**	1.1 - 14 April 2020	18-05-2020	15-05-2020
**AMENDMENT 1**	2.0 – 9 December 2021	15-03-2021	08-02-2021

**COORDINATING INVESTIGATOR**	Prof. Christophe LE TOURNEAU<br>Institut Curie<br>Department of Drug Development and Innovation (D3i)<br>26 rue d'Ulm -75005 PARIS<br>Phone: +33 (0)1 44 32 46 75<br>Email: christophe.letourneau@curie.fr
**SPONSOR**	**UNICANCER**<br>101, rue de Tolbiac<br>75654 Paris Cedex 13 (France)<br>Phone: + 33 (0)1 44 23 04 04 – Fax: + 33 (0)1 44 23 55 69

CONTACT DETAILS
PROTOCOL N°: UC-GMP-1908

Trial Title:

Phase II basket trial evaluating the efficacy of a combination of pembrolizumab and vorinostat in patients with recurrent and/or metastatic squamous cell carcinoma

**WRITING COMMITTEE**	Christophe Le Tourneau, Maud Kamal, Lilian Amrein, Marta Jimenez, Thomas Filleron

**CONTACT DETAILS AND USEFUL INFORMATION**	
**Prof. Christophe LE TOURNEAU**<br>**Coordinating Investigator**	Institut Curie<br>Department of Drug Development and Innovation (D3i)<br>26 rue d'Ulm -75005 PARIS<br>Phone: +33 (0)1 44 32 46 75<br>Email: christophe.letourneau@curie.fr
**Mr. Thomas FILLERON**<br>**Statistician**	Institut Claudius Regaud, IUCT-O<br>Department of biostatistics<br>1 avenue Irène Joliot-Curie<br>31059 TOULOUSE Cedex 9<br>Phone: +33 (0)5 31 15 58 65<br>Email: filleron.thomas@iuct-oncopole.fr
**Ms. Maud KAMAL**<br>**Ancillary/translational coordinator**	Institut Curie<br>Department of Drug Development and Innovation (D3i)<br>26 rue d'Ulm -75005 PARIS<br>Phone: +33 (0)1 44 32 44 20<br>Email: maud.kamal@curie.fr
**Mr. François LEGRAND**<br>**Project Manager**	R&D UNICANCER<br>101 rue de Tolbiac – 75654 PARIS Cedex 13<br>Phone: + 33 (0)1 73 79 73 02<br>Fax: + 33 (0)1 44 23 55 69<br>Email: f-legrand@unicancer.fr
**Pharmacovigilance Notification**	R&D UNICANCER Pharmacovigilance Unit<br>101 rue de Tolbiac – 75654 PARIS Cedex 13<br>Phone: + 33 (0)1 44 23 04 16<br>Fax: + 33 (0)1 44 23 55 70<br>Email: fax-pv@unicancer.fr

APPROVAL AND SIGNATURE
FOR PROTOCOL N°: UC-GMP-1908

Trial Title:

Basket phase II trial evaluating the efficacy of a combination of pembrolizumab and vorinostat in patients with recurrent and/or metastatic squamous cell carcinoma

Title	Name	Address	Date (dd-mm-yyyy)	Signature
**Director of Clinical Operations**	**Beata Juzyna**	R&D UNICANCER<br>101 rue de Tolbiac<br>75654 Paris cedex 13, France<br>Tel: +33 (0)1 71 93 61 60<br>Fax: +33 (0)1 44 23 55 69<br>Email: b-juzyna@unicancer.fr	Fait le 04/06/2021<br>Beata JUZYNA<br>Signé par Beata JUZYNA<br>Signé et certifié par yousign	
**Project Manager**	**François Legrand**	R&D UNICANCER<br>101 rue de Tolbiac<br>75654 Paris cedex 13, France<br>Phone: + 33 (0)1 73 79 73 02<br>Fax: + 33 (0)1 44 23 55 69<br>Email: f-legrand@unicancer.fr	Fait le 04/06/2021<br>François LEGRAND<br>Signé par François LEGRAND<br>Signé et certifié par yousign	
**Coordinating Investigator**	**Christophe Le Tourneau**	Institut Curie<br>26 rue d'Ulm - 75005 PARIS<br>Phone: +33 (0)1 44 32 46 75<br>Email: christophe.letourneau@curie.fr	Fait le 04/06/2021<br>Christophe LE TOURNEAU<br>Signé par Christophe LE TOURNEAU<br>Signé et certifié par yousign	
**Biostatistician**	**Thomas Filleron**	Institut Claudius Regaud, IUCT-O<br>TOULOUSE<br>Phone: +33 (0)5 31 15 58 65<br>Email: filleron.Thomas@iuct-oncopole.fr	Fait le 04/06/2021<br>Thomas FILLERON<br>Signé par Thomas FILLERON<br>Signé et certifié par yousign	

TABLE OF CONTENT

LIST OF ABBREVIATIONS 8

STATEMENT OF COMPLIANCE 10

PROTOCOL SUMMARY 11

Synopsis 11

Schedule of visits and activities (SOA) 21

1. INTRODUCTION 24

1.1. Background information 24

1.1.1. Disease epidemiology 24

1.1.2. Prognosis 24

1.1.3. Investigational medicinal products (IMP) 25

1.2. Trial rationale 25

1.2.1. Immunotherapy for treating SCC 26

1.2.2. Immune checkpoint inhibitors combined with HDAC inhibitors for treating SCC 27

1.2.3. Pembrolizumab combined with vorinostat for treating SCC 28

1.3. Justification for the therapeutic regimens and treatment durations 28

1.4. Potential risks and benefits 29

1.4.1. Known potential risks 29

1.4.2. Known potential benefits 30

1.5. Trial population 30

2. TRIAL OBJECTIVES 32

2.1. Primary objective 32

2.2. Secondary objectives 32

2.3. Translational objectives 32

3. TRIAL DESIGN AND ENDPOINTS 33

3.1. Description of the trial Design 33

3.2. Trial Endpoints 33

3.2.1. Primary endpoint 33

3.2.2. Secondary endpoint(s) 33

3.3. Translational endpoints 34

3.4. Progression of the trial 34

3.5. Inclusion procedure 35

3.6. Premature Trial Terminations and Suspension 35

3.7. Patient's trial withdrawal and discontinuation 35

4. PATIENT SELECTION 36

4.1. Diagnosis and inclusion criteria 36
4.2. Non-inclusion criteria 37

5. TRIAL TREATMENTS/INTERVENTIONS 39

5.1. Description of trial treatments/interventions 39
5.1.1. Pembrolizumab 39
5.1.2. Vorinostat 39
5.2. Acquisition, reception, and storage 39
5.3. Trial treatments accountability, return and destruction 40
5.4. Formulation, appearance, packaging, and labelling 40
5.4.1. Pembrolizumab 40
5.4.2. Vorinostat 40
5.5. Preparation 41
5.5.1. Pembrolizumab 41
5.5.2. Vorinostat 41
5.6. Dose adaptation 41
5.6.1. Pembrolizumab 41
5.6.2. Vorinostat 41
5.7. Discontinuation of treatments: 42
5.8. Concomitant medications and therapies 43
5.8.1. Authorized concomitant treatments 43
5.8.2. Prohibited concomitant treatments 43
5.8.3. Rescue medications and therapies/Treatment at disease progression 44
5.8.4. Contraception during the trial 44

6. EVALUATION OF TREATMENT ACTIVITY AND SAFETY 47

6.1. Activity evaluation 47
6.1.1. Tumor imaging during the study 47
6.1.2. End of treatment and follow-up tumor imaging 48
6.1.3. RECIST v1.1 assessment of disease 48
6.1.4. iRECIST assessment of disease 49
6.1.5. Blinded independent central review committee (BICR): 49
6.2. Safety evaluation 49
6.2.1. Physical examination 50
6.2.2. Vital signs 50
6.2.3. Clinical laboratory tests 50

7. DESCRIPTION OF VISITS AND INVESTIGATIONS 51

7.1. Baseline visit 51

7.2. Visits and assessment during treatment period 52
7.3. End-of-treatment visit 53
7.4. Follow-up 54
7.5. Provisions in case of treatment or trial interruption 55

8. REPORTING OF ADVERSE EVENTS 56

8.1. Adverse event: general definition 56
8.2. Serious adverse event: general definition 56
8.3. Measures to be taken in case of a serious adverse event 57
8.4. Events of Clinical Interest (ECIs) 59

9. TRANSLATIONAL STUDIES 60

10. DESCRIPTION OF STATISTICAL METHODS 61

10.1. Statistical hypothesis and sample size determination 61
10.2. Trial populations to be analyzed 61
10.3. Planned statistical analysis 62
10.3.1. Modifications of SAP and initial statistical analysis strategy 62

11. OVERSIGHT COMMITTEES 63

11.1. Independent data monitoring committee 63
11.2. Steering Executive Committee 63

12. QUALITY ASSURANCE 65

12.1. Data collection 65
12.2. Access to data 66
12.3. Trial monitoring 66
12.4. Audits and inspections 67

13. ETHICAL AND REGULATORY CONSIDERATIONS 68

13.1. General requirements 68
13.2. Patient identification 68
13.3. Patient information and consent 68
13.4. Insurance compensation 69
13.5. Investigator responsibilities 69
13.6. Federation of the Patient Committees for Clinical Research in Cancerology 70
13.7. Human biological samples collection 70
13.7.1. Storage and use of disease assessment samples (blood, biopsy, tumor specimen, etc.) 70
13.7.2. Collecting additional biological samples for research purpose 71

14. DATA PROCESSING AND CONSERVATION OF DOCUMENTS AND DATA OF THE RESEARCH 72

14.1. Data processing.....72

14.1.1. Under the responsibility of the sponsor 72

14.1.2. In the investigational center, when computerized medical records are used 72

14.2. Retention of documents by investigator sites.....72

15. DATA OWNERSHIP AND CONFIDENTIALITY 74

16. PUBLICATION RULES 74

17. REFERENCES 76

18. APPENDICES79

Appendix 1. Performance status evaluation – WHO scale.....80

Appendix 2. World Medical Association - Declaration of Helsinki.....81

Appendix 3. ICH Harmonised Tripartite Guideline for Good Clinical Practice (ICH-GCP).....82

Appendix 4. List of medications with (a) defined risk, (b) possible, and (c) conditional risk of Torsade de Pointes 83

Appendix 5. Corticosteroid Dose Equivalents.....84

Appendix 6. A brief summary of tumor classification RECIST v1.1.....85

Appendix 7. Description of the iRECIST Process for Assessment of Disease Progression.....88

Appendix 8. Toxicity criteria (NCI-CTCAE).....93

Appendix 9. Dose modification and toxicity management guidelines for immune-related AEs associated with pembrolizumab.....94

Appendix 10. Dose modification and toxicity management of infusion-reactions related to pembrolizumab 97

Appendix 11. Dose modification and toxicity management of drug reactions related to vorinostat.....99

Appendix 12. Translational studies.....101

LIST OF ABBREVIATIONS

AE	Adverse Event
ALT	Alanine Aminotransferase
ANC	Absolute Neutrophil Count
ANSM	Agence Nationale De Sécurité Du Médicament Et Des Produits De Santé
aPTT	Activated Partial Thromboplastin Time
AST	Aspartate Aminotransferase
βHCG	Beta Human Chorionic Gonadotropin
BCG	Bacillus Calmette–Guérin
BP	Blood Pressure
CI	Confidence Interval
CNS	Central Nervous System
CPP	Comité De Protection Des Personnes
CR	Complete Response
CRA	Clinical Research Associate
CRO	Contract Research Organization
CTCAE	Common Terminology Criteria For Adverse Events
CT-Scan	Computed Tomography Scanner
DCF	Data Correction Form
DMR	Differentially Methylated Regions
DOR	Duration Of Response
DSMB	Data And Safety Monitoring Board
ECG	Electrocardiogram
ECOG	Eastern Cooperative Oncology Group
eCRF	Electronic Case Report Form
FDA	Food And Drug Administration
FFPE	Formalin-Fixed, Paraffin-Embedded
g	Gram
GCP	Good Clinical Practice
GGT	Gamma-Glutamyl Transferase
HBsAg	Hepatitis B Surface Antigen
HBV	Hepatitis B Virus
HCV	Hepatitis C Virus
HDAC	Histone Deacetylase
HIV	Human Immunodeficiency Virus
HLA	Human Leukocyte Antigen
H&N	Head and Neck
HNSCC	Head and neck squamous cell carcinoma
HPV	Human Papilloma Virus
IB	Investigator Brochure
IBBL	Integrated BioBank of Luxembourg
IC	Insitut Curie
ICF	Inform Consent Form
ICH	International Conference On Harmonization
IDMC	Independent Data Monitoring Committee
IEO	Istituto Europeo di Oncologia
IHC	Immuno Histo Chemestry
IMF	Investigator Master File

IMF-P	Investigator Master File-Pharmacy
INN	International Non-Proprietary Name
INR	International Normalized Ratio
iORR	Immune Objective Response Rate
iPFS	Immune Progression Free Survival
IP	Investigational Products
irAEs	Immune-Related Adverse Events
iRECIST	Immune-Specific Response Evaluation Criteria In Solid Tumors
IV	Intravenous
L	Liter
mg	Milligram(S)
min	Minute(S)
mL	Milliliter(S)
MSI-H	Microsatellite instability -high
NCI	National Cancer Institute
NSCLC	Non-small cell lung cancer
ORR	Objective Response Rate
OS	Overall Survival
PBMC	Peripheral Blood Mononuclear Cells
PD	Progressive Disease
PD-1	Programmed Cell Death-1
PD-L1	Programmed Cell Death-Ligand 1
PR	Partial Response
PFS	Progression-Free Survival
PT	Prothrombin Time
PUVA	Psoralen Plus Ultraviolet A Radiation
Q3W	Every 3 Weeks
RBC	Red Blood Cell
RECIST	Response Evaluation Criteria In Solid Tumors
RNA	Ribonucleic Acid
SAE	Serious Adverse Event
SAP	Statistical Analysis Plan
SCCa	Squamous Cell Carcinoma Antigen
SCC	Squamous cell carcinoma
SD	Stable Disease
SmPC	Summary Of Products Characteristics
SOA	Schedule Of Visits And Activities
SUSAR	Suspected Unexpected Serious Adverse Reaction
TME	Tumor Microenvironment
TMF	Trial Master File
TSH	Thyroid Stimulating Hormone
ULN	Upper Limit Normal
ULN	Upper Limit Of Normal
VKA	Vitamin K Antagonist
WBC	White Blood Cells

STATEMENT OF COMPLIANCE

UNICANCER, the trial sponsor, certifies that the trial PEVOsq will be conducted in compliance with the protocol described in this document, and in accordance with the French national regulatory requirements:

- *Declaration of Helsinki, as modified in 2008.*
- *Loi n°2012-300 du 5 mars 2012 relative aux recherches impliquant la personne humaine, as modified in 2016.*
- Regulation (EU) 2016/679 on the protection of natural persons with regard to the processing of personal data and on the free movement of such data (General Data Protection Regulation).
- Amended *Loi Informatique et Libertés n° 78-17 du 6 janvier 1978, relative à la protection des personnes physiques à l'égard des traitements de données à caractère personnel*.
- Amended *Loi n° 2004-800 du 6 août 2004, relative à la bioéthique*.
- *Décision du 24 novembre 2006 fixant les règles de bonnes pratiques cliniques pour les recherches biomédicales portant sur des médicaments à usage humain.*
- *Arrêté du 24 mai 2006 relatif au contenu et aux modalités de présentation d'un protocole de recherche biomédicale portant sur un médicament à usage humain.*
- Good Manufacturing Practices, in particular, Annex 13 on investigational medicinal products.

PROTOCOL SUMMARY

SYNOPSIS

A) TRIAL IDENTIFICATION	
**SPONSOR – PROTOCOL CODE NUMBER:** UC-GMP-1908	
**VERSION (NUMBER & DATE):** v3.2, 21st May 2021	
**TRIAL TITLE:** Phase II basket trial evaluating the efficacy of a combination of pembrolizumab and vorinostat in patients with recurrent and/or metastatic squamous cell carcinoma<br>**PHASE:** II	
**TRIAL TITLE FOR LAY PEOPLE:** Efficacy of immunotherapy in combination with a drug in patients with advanced mucosal cancer of different locations that has progress.	
**ABBREVIATED TITLE:** PEVOsq: PEmbrolizumab and VOrinostat in patients with recurrent and/or metastatic SQuamous cell carcinoma	
**COORDINATING INVESTIGATOR:** Christophe Le Tourneau, Institut Curie, Paris, France	
**STATISTICIAN :** Thomas Filleron, Institut Claudius Regaud, IUCT-O, Toulouse, France	
**NUMBER OF CENTERS:** around 15	**NUMBER OF PATIENTS:** 111

B) SPONSOR IDENTIFICATION	
**NAME:**	**UNICANCER**<br>101, rue de Tolbiac<br>75654 Paris Cedex 13<br>France
**CONTACT PERSON:**	**MR FRANCOIS LEGRAND**<br>**PROJECT MANAGER**<br>**R&D UNICANCER**<br>Tel: +33 (0) 1 73 79 73 02<br>email: f-legrand@unicancer.fr

C) TRIAL GENERAL INFORMATION
**INDICATION:**<br>Patients with recurrent and/or metastatic squamous cell carcinoma of the head and neck, cervix, lung, anus, vulva/vagina, and penis.

**TRIAL DESCRIPTION/DESIGN:**<br><br>Open-label, non-randomized, multi-center, basket phase II trial, evaluating the efficacy pembrolizumab in combination with vorinostat in adult patients with recurrent and/or metastatic squamous cell carcinoma of different locations.
**PRIMARY OBJECTIVE:**<br><br>To evaluate the antitumor activity of pembrolizumab in combination with vorinostat in patients with recurrent and/or metastatic squamous cell carcinoma of the head and neck, cervix, lung, anus, vulva/vagina, and penis, using the ORR during treatment (investigator assessment).
**SECONDARY OBJECTIVES:**<br><br>• To determine, in each cohort, the anti-tumor activity in term of:<br>➢ Centrally confirmed objective response rate (ORR), as per RECIST v1.1.<br>➢ Centrally confirmed immune objective response rate (iORR), as per iRECIST.<br>➢ Duration of response (DOR).<br>➢ Progression-free survival (PFS), as per RECIST.<br>➢ Immune-progression-free survival (iPFS), as per iRECIST.<br>➢ Overall survival (OS).<br>• To evaluate the safety and tolerability of pembrolizumab in combination with vorinostat according to NCI CTCAE v5.0:<br>➢ In each cohort.<br>➢ In the overall study population.
**TRANSLATIONAL OBJECTIVES:**<br><br>The translational studies associated with this clinical study aim to assess the following:<br>• Link between the tumor molecular profile and immune parameters.<br>• Link between immune-related biomarkers and drug combination efficacy.<br>• Post-treatment modification of immune-related and molecular epigenetic biomarkers.<br>• Predictive value of tumor microenvironment and epigenetic parameters.<br>• Predictive value of circulating biomarkers.
**DIAGNOSIS AND INCLUSION CRITERIA:**<br><br>1. Aged ≥18 years old.<br>2. Patients with Eastern Cooperative Oncology Group (ECOG) performance status ≤1.<br>3. Patients must have histologically confirmed recurrent and/or metastatic squamous cell carcinoma of the head and neck, cervix, lung, anus, vulva/vagina, or penis.<br>4. Patients must have radiologically confirmed progressive recurrent and/or metastatic disease.<br>5. Patients naive or previously treated for recurrent and/or metastatic disease.<br>6. Disease amenable to biopsy for study purpose.<br>7. Measurable disease according to RECIST v1.1.

8. Adequate renal function: serum creatinine ≤1.5 x upper limit of normal (ULN) (OR creatinine clearance [Cockcroft and Gault] ≥30 mL/min for participant with creatinine levels >1.5 × ULN) within 14 days prior inclusion.
9. Adequate liver function: aspartate aminotransferase (AST) and alanine aminotransferase (ALT) levels ≤2.5 × ULN (≤5 ULN when documented liver metastases) and total bilirubin level ≤1.5 × ULN, within 14 days prior inclusion.
10. Adequate bone marrow function: absolute neutrophil count (ANC) ≥1,500/mm³, platelet count ≥100,000/mm³, and hemoglobin ≥9 g/dL, within 14 days prior inclusion.
11. Adequate coagulation: prothrombin time (PT)/international normalized ratio (INR) ≤1.5 × ULN within 14 days prior inclusion If participant is receiving anticoagulant therapy then the PT or activated partial thromboplastin time (aPTT) should be within the therapeutic range of intended use of anticoagulant.
12. Female of child-bearing potential must have a negative serum pregnancy test within 72 h before starting study treatment.
13. Female of childbearing potential, must use "highly effective" methods of contraception for the study duration and for 4 months following the last dose of pembrolizumab and 6 months following the last dose of vorinostat.
14. Male participants must agree to use an effective contraceptive for the duration of the trial and for at least 4 months after the last the last dose of pembrolizumab and 6 months following the last dose of vorinostat (to allow for effective elimination of the study drugs). Also, they should refrain from donating sperm during this period.
15. Patients must be willing and able to comply with the protocol for the duration of the study including scheduled visits, treatment plan, and laboratory tests.
16. Patients must be willing and able to comply with other study procedures, including a baseline tumor biopsy and a series of blood samples throughout the study.
17. Patients able to swallow oral medications.
18. Patients must be affiliated to a Social Security System (or equivalent).
19. Patients must have signed a written informed consent prior to any trial-specific procedures. When the patient is physically unable to give their written consent, a trusted person of their choice, independent from the investigator or the sponsor, can confirm in writing the patient's consent.

NON-INCLUSION CRITERIA:

1. Prior treatment with anti-PD-1/PD-L1 agents or histone deacetylases (HDAC) inhibitors.
2. Patients with central nervous system involvement that has not been controlled for >3 months.
3. Patients with no other site for biopsy than bone lesions.
4. Patients with other concurrent severe and/or uncontrolled medical disease which could compromise participation in the study, including uncontrolled diabetes, cardiac disease, uncontrolled hypertension, congestive cardiac failure, ventricular arrhythmias, active ischemic heart disease, myocardial infection within one year, chronic liver or renal disease, active gastrointestinal tract ulceration, severely impaired lung function.

5. Known history of human immunodeficiency virus (HIV), Hepatitis B virus (HBV; defined as Hepatitis B surface antigen [HBsAg] reactive) or known active Hepatitis C virus (HCV; defined as HCV RNA detected) virus infection.
6. History of autoimmune disease with the exception of:
   - (1) Patients with a history of autoimmune hypothyroidism on a stable dose of thyroid replacement hormone,
   - (2) Patients with controlled Type 1 diabetes mellitus on a stable insulin regimen,
   - (3) Patients with eczema, psoriasis, lichen simplex chronicus, or vitiligo with dermatologic manifestations only (e.g., patients with psoriatic arthritis would be excluded) provided that they meet the following conditions: (i) Rash must cover less than 10% of body surface area; (ii) Disease is well controlled at baseline and only requiring low potency topical steroids; (iii) No acute exacerbations of underlying condition within the previous 12 months (not requiring psoralen plus ultraviolet A radiation [PUVA], methotrexate, retinoid, biologic agents, oral calcineurin inhibitors, high-potency or oral steroids).
7. History of allogeneic organ or bone marrow transplantation.
8. History of non-infectious pneumonitis that required steroids or has current pneumonitis.
9. Has an active infection requiring systemic therapy.
10. Has received a live vaccine within 30 days prior to the first dose of study drug. Examples of live vaccines include, but are not limited to, the following: measles, mumps, rubella, varicella/zoster (chicken pox), yellow fever, rabies, Bacillus Calmette–Guérin (BCG), and typhoid vaccine. Seasonal influenza vaccines for injection are generally killed virus vaccines and are allowed; however, intranasal influenza vaccines (eg, FluMist®) are live attenuated vaccines and are not allowed.
11. Known prior severe hypersensitivity to investigational products or its excipients,
12. Has received prior systemic anti-cancer therapy including investigational agents within 4 weeks [could consider shorter interval for kinase inhibitors or other short half-life drugs] prior to first dose of study treatments.

    Note: Participants must have recovered from all AEs due to previous therapies to ≤Grade 1 or baseline. Participants with ≤Grade 2 neuropathy may be eligible.
13. Has received prior radiotherapy within 2 weeks of start of study treatment. Participants must have recovered from all radiation-related toxicities, not require corticosteroids, and not have had radiation pneumonitis. A 1-week washout is permitted for palliative radiation (≤2 weeks of radiotherapy) to non-CNS disease.
14. Major surgery within 28 days prior to the first dose of study treatments.

    Note: Local surgery of isolated lesions for palliative intent is acceptable.
15. Current or prior use of immunosuppressive medication within 7 days before the first dose of pembrolizumab. The following are exceptions to this criterion:
    - Intranasal, inhaled, topical steroids, or local steroid injections (e.g., intra articular injection),

- Systemic corticosteroids at physiologic doses ≤10 mg/day of prednisone or its equivalent,
- Steroids as premedication for hypersensitivity reactions (e.g., CT scan premedication).

16. Patients using drugs that could have pharmacokinetics interaction with investigational drugs. This includes, but is not limited to, valproic acid, coumarin-derivative anticoagulants, drugs that disrupt electrolyte levels, drugs that may prolong QT.
17. Pregnant women or women who are breast-feeding.
18. Patients enrolled in another therapeutic study within 30 days prior to inclusion and during the treatment period. Patients can participate in an independent approved non-interventional studies.
19. Patients unwilling or unable to comply with the medical follow-up required by the trial because of geographic, familial, social, or psychological reasons.
20. Persons deprived of their liberty or under protective custody or guardianship.

PRIMARY ENDPOINT:

Investigators will assess the ORR. The ORR is defined in each cohort as the percentage of evaluable patients for ORR, designate as the proportion of patients with best response of complete response (CR) or a partial response (PR) during treatment according to RECIST v1.1.

SECONDARY ENDPOINTS:

- Anti-tumor activity endpoints will be evaluated in each cohort:
  - ORR defined as the proportion of patients with best response of CR or PR during treatment, as assessed by a central radiological panel according to RECIST v1.1.
  - iORR defined as the proportion of patients with best response of CR or PR during treatment, as assessed by a central radiological panel according to immune-specific response criteria (iRECIST).
  - DOR will be evaluated in patients with either a complete response (CR) or partial response (PR). DOR is defined as the time from the first assessment of a CR or PR until the date of the first occurrence of progressive disease (PD) or death from any cause (if death occurred within predefined period), whichever occurs first.
  - PFS is defined per RECIST1.1 as the time from inclusion until disease progression (per RECIST v1.1) or death from any cause, whichever occurs first. At the time of analysis, a patient alive and without disease progression will be censored at the date of the last tumor assessment. Patients alive without disease progression who started a new anticancer therapy will be censored at the date of the last tumor assessment prior to the start of the new anticancer therapy.
  - iPFS is defined per iRECIST as the time from inclusion until confirmed disease progression (per iRECIST), or death from any cause, whichever occurs first. At the time of analysis, a patient alive and without progression will be censored at the date of the last tumor assessment. Patients alive without disease progression who started a new anti-cancer therapy will be censored at the date of the last tumor assessment prior to the start of the new anticancer therapy.

- OS is defined as the time from inclusion until death from any cause. Patients who are alive at last follow-up news will be censored at this date.

- The safety will be evaluated according to the incidence of adverse events (AEs) graded by NCI-CTCAE v5.0:
  - In each cohort.
  - In the overall study population.

TRANSLATIONAL ENDPOINTS:

- To assess the link between the tumor molecular profile including epigenetics features and immune parameters of the tumor microenvironment (TME) and in blood samples.
- To assess the link between immune-related biomarkers in the TME and in blood samples (including but not limited to tumor tissue PD-L1 expression by IHC, RNA gene expression profiling, and DNA mutation analysis), and measures of efficacy.
- To explore the modification of immune-related and molecular epigenetic biomarkers following treatment.
- To analyze the impact of TME and epigenetics parameters on sensitivity/response to treatment.
- To assess the predictive value of circulating biomarkers on sensitivity or resistance to treatment.

D) INVESTIGATIONAL MEDICINAL PRODUCTS

PRODUCT NAMES AND ADMINISTRATION:

Drug name (INN)	Registered name	Pharmaceutical form	Administration route	Posology
Pembrolizumab	Keytruda	100 mg/vial (liquid)	Intra-venous (IV)	200 mg every 3 weeks
Vorinostat	Zolinza®	100 mg/capsule	Per os	400 mg once daily

THERAPEUTIC REGIMENS:

Patients will take vorinostat orally at 400 mg once daily with food.

Pembrolizumab will be administered intravenously at 200 mg every 3 weeks up to 35 administrations (approximately 2 years).

Treatment will be administered until disease progression, unacceptable toxicity, or patient's decision, and up to 35 administrations for pembrolizumab.

Continuation of pembrolizumab beyond 35 cycles may be allowed under certain conditions and must be agreed with the sponsor before continuing the treatment.

TRIAL FLOWCHART:

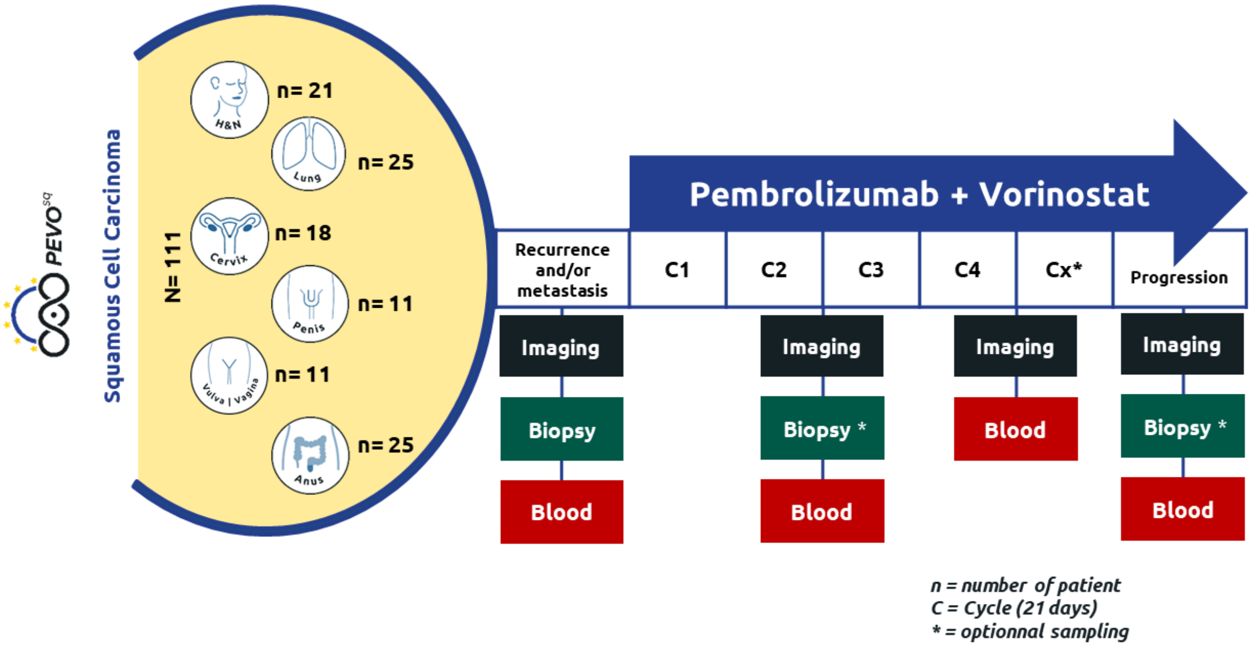

Study Design: A fresh tumor biopsy is mandatory prior to treatment (baseline). Optional biopsies might be collected within the week before the first disease assessment and at disease progression. Imaging will be performed every 6 weeks. Blood sampling for research will be performed at baseline before pembrolizumab plus vorinostat treatment, at Day 1 of cycle 3 and cycle 5, and at disease progression.

C1-Cx: The duration of each treatment cycles is 3 weeks.

TREATMENT DURATION:

Patients will be treated until disease progression (or for up to 35 cycles for pembrolizumab) or unacceptable toxicity or patient decision.

Continuation of pembrolizumab beyond 35 cycles may be allowed under certain conditions and must be agreed with the sponsor before continuing the treatment.

E) STATISTICAL ANALYSIS PLAN

REQUIRED NUMBER OF PATIENTS TO BE SCREENED/INCLUDED: 111

The primary aim of the study is to assess the antitumor activity of the combination of pembrolizumab and vorinostat in patients with selected recurrent and/or metastatic solid tumors.

ORR reported in the literature with pembrolizumab or nivolumab in phase III clinical trials ranged from 6% to 24% depending on the tumor type.

The primary endpoint is the rate of patient presenting an objective response (defined as the proportion of patients with best response of a complete response (CR) or a partial response (PR) according to RECIST v1.1) while on study protocol.

The required number of evaluable patients for each cohort was determined using an A'Hern design (A'Hern 2001) based on different hypotheses. Design parameters and decision rules for each cohort are summarized in the following table:

Cohort	p0	p1	Alpha	Power	Nb of evaluable / included patients	Sufficiently active
Head and Neck	10%	35%	5%	85%	19 / 21	≥5 CR/PR
Cervix	5%	30%	5%	90%	16 / 18	≥3 CR/PR
Lung	15%	40%	5%	85%	23 / 25	≥7 CR/PR
Anus	15%	40%	5%	85%	23 / 25	≥7 CR/PR
Penis	5%	30%	10%	85%	10 /11	≥2 CR/PR
Vulva/vagina	5%	30%	10%	85%	10 / 11	≥2 CR/PR

- p0: maximal unacceptable rate of patient presenting an objective response for whom the experimental treatment will be considered as insufficiently active.
- p1: minimal acceptable rate of patient presenting an objective response for whom the experimental treatment will be considered as sufficiently active.

To compensate for drop out, we plan to include an additional 10% of patients in each cohort; therefore a total of 111 patients are required for this study.

Cohort	Nb of evaluable patients	Insufficiently active	Sufficiently active
Penis, vulva/vagina	10	<2 CR/PR	≥2 CR/PR
Cervix	16	<3 CR/PR	≥3 CR/PR
Head & Neck	19	<5 CR/PR	≥5 CR/PR
Lung, Anus	23	<7 CR/PR	≥7 CR/PR

STATISTICAL ANALYSIS:

Demographic and clinical data

Demographic and clinical characteristics (including HPV status) will be presented in the overall population and per cohort using usual statistics. Quantitative data will be summarized as median, min, max, and number of missing data. Qualitative variables will be described as number, percentage, and number of missing data.

Population definition

The following populations will be considered for analyses:

- Per-protocol: all eligible patients with at least one valid post-baseline disease assessment (or with disease progression) and have received at least one dose of the study treatments (pembrolizumab or vorinostat).

- Safety population: all patients who initiated allocated treatment (at least one dose of the study treatments).

Primary endpoint

The primary endpoint will be assessed in the per protocol population and will be reported per cohort. The primary endpoint is the rate of evaluable patients for response presenting an objective response assessed by the investigators according to RECISTv1.1. It will be presented as number, percentage, and 95% confidence interval (CI): by the binomial exact distribution.

Secondary endpoints

The secondary efficacy endpoints will be assessed in the per protocol population and will be reported per cohort.

ORR, iORR, and DOR will be presented using frequency, percentage frequencies, percentages, and the 95% CI (Binomial exact distribution).

A radiological panel will centrally assess the ORR, iORR, and iPFS.

Survival rates (PFS, iPFS OS) will be estimated at different time points using the Kaplan-Meier method. Median survival times will be estimated and reported with the corresponding 95% CI.

Number of pembrolizumab injections and dose intensity of vorinostat will also be recorded.

Incidence rates of adverse events and serious adverse events will be presented using frequencies and percentages by system organ class and MedDRA preferred term. Pharmacovigilance will be handled by UNICANCER.

F) SAMPLES COLLECTED FOR TRANSLATIONAL RESEARCH

SAMPLE TYPES:	SAMPLE QUANTITIES:	TIMEPOINTS:
**Blood samples**		
Mandatory	2 x 10 mL<br>PACgene CCF tubes	Baseline,<br>C3D1, C5D1, Progression
Mandatory	3 x 8. mL CPT tubes	Baseline,<br>C3D1, C5D1, Progression
**Tumor Samples**		
Mandatory	Up 4 cores:<br>2 FFPE, 2 frozen	Baseline
Optional	Up 4 cores:<br>2 FFPE, 2 frozen	C3D1, Progression

G) TRIAL DURATIONS
**INCLUSION PERIOD:** 18 months
**TRIAL TREATMENT PERIOD:** Until disease progression (or up to 35 cycles for pembrolizumab) or unacceptable toxicity or patient's decision. Approximately 6 months
**POST-TREATMENT FOLLOW-UP:** 1 year
**DURATION UNTIL PRIMARY ENDPOINT EVALUATION:** 18 + 24 = 42 months
**OVERALL TRIAL DURATION (INCLUDING FOLLOW-UP):** 18+24+12 = 54 months approximately

SCHEDULE OF VISITS AND ACTIVITIES (SOA)

A general +/-3 days window is allowed for assessments or visits performed, unless otherwise indicated.

**VISITS**	**Baseline**	**Treatment phase**					**End of study treatment visit [10]**	**Follow-up [11]**
**Visit Dates**	**Day -28 to 0**	**Day 1**	**Day 22**	**Day 43**	**Day 64**	**Every 21 days**		
**CYCLES (every 3 weeks)**		**C1**	**C2**	**C3**	**C4**	**C5 and after**		
**Patient history**								
Inclusion/non-inclusion criteria	D-28							
Signed informed consent form	D-28							
Demography	D-28							
Medical history and prior cancer treatment	D-28							
History of HIV, Hepatitis B and C	D-28							
**Physical examination**								
Clinical examination [1]	D-14		D1(+/-3d)		D1(+/-3d)	D1(+/-3d)		
Weight, ECOG	D-14	D1(+/-3d)	D1(+/-3d)	D1(+/-3d)	D1(+/-3d)	D1(+/-3d)	X	
Adverse events	D-28	D1(+/-3d)	D1(+/-3d)	D1(+/-3d)	D1(+/-3d)	D1(+/-3d)	X	X
Post study anticancer treatment								X [12]
**Paraclinical examination**								
Tumor measurements as per RECIST1.1 and iRECIST (CT-scan or MRI) [2, 22]	D-14			D1(-7d)		D1(-7d) [2]	X	X [3]
12 lead electrocardiogram	D-14							
**Biological tests**								
Hematology [4]	D-14	D1(+/-3d) D8[15],D15	D1(+/-3d) D8[15],D15[14]	D1(+/-3d) D8[15],D15[15]	D1(+/-3d) D8[15],D15[15]	D1(+/-3d)	X	
Biochemistry [5]	D-14	D1(+/-3d) D8[15],D15[14]	D1(+/-3d) D8[15],D15[15]	D1(+/-3d) D8[15],D15[15]	D1(+/-3d) D8[15],D15[14]	D1(+/-3d)	X	
Thyroid function test [6]	D-14	D1(+/-3d)		D1(+/-3d)		D1(+/-3d) [6]		
Squamous cell carcinoma antigen (SCCa) [6]	D-14			D1(+/-3d)		D1(+/-3d) [6]	X	
Serum pregnancy test [7]	D-3 (72 h)		D1(+/-3d)	D1(+/-3d)	D1(+/-3d)	D1(+/-3d)	X	

VISITS	Baseline	Treatment phase					End of study treatment visit [10]	Follow-up [11]
**Visit Dates**	**Day -28 to 0**	**Day 1**	**Day 22**	**Day 43**	**Day 64**	**Every 21 days**		
**CYCLES (every 3 weeks)**		**C1**	**C2**	**C3**	**C4**	**C5 and after**		
**Treatments**								
Pembrolizumab [8]		D1	D1	D1	D1	D1		
Vorinostat [9]		Every day						
**Translational research (mandatory)**								
Blood samples (30 to 40 mL each)	D-14			D1(+/-7d)		D1(+/-7d) [13]	At progression	
Mandatory tumor biopsy	D-14 [14]							
**Translational research (optional)**								
Optional tumor biopsy				D1(+/-7d) [14]			At progression [14]	

Notes:

1. Includes pulse rate, systolic and diastolic blood pressure, and body temperature.
2. CT scan or MRI of the chest, abdomen, and pelvis should be performed for tumor assessment according to RECIST/ iRECIST. Imaging disease assessments should be obtained every 6 weeks (within 7 days prior to CxD1), or sooner if clinically indicated, during the first 12 months of treatment phase, and every 12 weeks (within 7 days prior to treatment) thereafter. They should be repeated 4 weeks after assessment of a PR or CR as per RECIST and after assessment of iUPD as per iRECIST guidelines. Tumor assessments must be performed regardless of changes to the study treatment administration schedule (e.g., dose delay).
3. Tumor evaluations are to be continued during the post-treatment follow-up period if withdrawal was not related to disease progression. Evaluations should be continued and documented every 6 weeks (or every 12 weeks after the first 12 months of treatment phase) until disease progression or initiation of an antineoplastic treatment.
4. Hemoglobin, red blood cell count, white blood cell count (WBC), absolute neutrophil count (ANC), lymphocyte count, and platelet count.
5. Includes blood ionogram (Na, K, Ca, Mg, P, Cl, CO_2, total protein, albumin), renal function (blood urea, creatinine), hepatic function (direct bilirubin, alkaline phosphatase, ALT, AST, GGT), coagulation profile (PT/INR, aPTT) – Baseline only, fasting plasma glucose, creatinine phosphokinase.
6. Thyroid function tests include TSH, free T3, free T4, and SCCa should be performed every 2 cycles starting with C3D1.
7. Serum pregnancy test (pre-menopausal patients only) within 72 h prior to starting study treatment, and at each 21-day cycle.
8. Pembrolizumab will be administered IV, 200 mg, at day 1 of a 3-week cycle, and up to 35 administrations (approximately 2 years).
9. Vorinostat will be taken orally, 400 mg, once daily (starting on day 1), until disease progression or unacceptable toxicity. Dose adaptation are described in section 5.6
10. Patients who discontinue study drug will return to the clinic for a treatment discontinuation visit 1 week after the last dose of all study drugs.
11. Patients should be followed up every 3 months (maximum of 1 year) until death, withdrawal of consent, or lost to follow-up.
12. Information on the first systemic anti-cancer therapy initiated after the first disease progression will be collected at the same time as survival assessments.
13. To be collected at C5D1 only
14. When possible, up to 4 cores (2 frozen, 2 FFPE) will be collected at each time points. Biopsies must be done on the same site as the baseline sample. If the sample volume is not sufficient to obtain four biopsy cores, priority will be given according to the following algorithm:

Priority order for biopsy cores collection depending on the tumor location/volume	Number of cores collected	Cores' preparation
1 (favorable situation)	4	2 Frozen, 2 FFPE

2	3	2 Frozen, 1 FFPE
3	2	1 Frozen, 1 FFPE
4 (unfavorable situation)	1	1 Frozen

15. Additional blood test to evaluate platelet count creatinine level, glucose, electrolytes, magnesium and calcium will be performed at C1D8, C1D15, C2D8, C2D15, C3D1, C3D15. Further evaluation from Cycle 4 at CxD8 CxD15 will be performed in case of grade ≥ 2 abnormalities observed on creatinine level or platelets count during the first 3 cycles.

1. INTRODUCTION

1.1. Background information

1.1.1. *Disease epidemiology*

Squamous cell carcinomas (SCC) can originate from various location within the body. In humans, they are among the most frequently occurring solid cancers (Dotto and Rustgi 2016). Indeed, worldwide in 2018, there were 2.1 million new cases of lung cancers (Bray, Ferlay et al. 2018). Of these, 85% are NSCLCs of which 30% are SCC (Heist, Mino-Kenudson et al. 2012, Derman, Mileham et al. 2015). H&N SCC are also fairly common, with 600,000 new cases of H&N SCC declared globally each year (Mangano, Mangano et al. 2015, Marur and Forastiere 2016). In contrast to lung SCC and H&N SCC, other SCCs are quite rare. Penile SCC is extremely rare with an incidence between 0.1-0.9 cases per 100,000 men, in Europe (Novara, Galfano et al. 2007). As is vulvar SCC, with an age-specific incidence rate of 0.4 per 100,000 women in their 30's increasing to 20 per 100,000 in women older than 70 years old (Gadducci, Tana et al. 2012). Anal SCC has a global annual incidence of about 1-3 cases per 100,000 individuals, with yearly about 48,000 new cases diagnosed worldwide (Bray, Ferlay et al. 2018, Morton, Melnitchouk et al. 2018, Eng, Messick et al. 2019). Finally, cervical SCC represents about 80% of cervical cancers (Li, Liu et al. 2016).

Numerous environmental factors including UV exposure, cigarette smoking, alcohol consumption, and infections favor the development of SCCs (Dotto and Rustgi 2016). Infectious agents such as the human papilloma virus (HPV) infections are implicated in most cervical SCCs and anal SCCs, a significant number of vulvar and penis SCC, and to a lesser extend HNSCC (Osborne, Maykel et al. 2014, Dotto and Rustgi 2016, Brianti, De Flammineis et al. 2017, Brotherton, Budd et al. 2020). In contrast, mycobacterium tuberculosis infection, but not HPV, is associated with lung SCC (Dotto and Rustgi 2016). However, one of the main environmental factor for developing lung SCC, as well as H&N SCC, is cigarette smoking (Dotto and Rustgi 2016).

1.1.2. *Prognosis*

The prognosis of patients with SCC varies largely according to the location. In patients with local or regional H&N SCC disease recurrences occurs in about 30% of patients (Cooper, Pajak et al. 2004). In addition, about 25% will develop distant metastases. The 5-year survival rate is 40%. Concerning lung SCC, it is difficult to diagnose at an early stage, and is less sensitive to radiotherapy and chemotherapy (Wang, Wang et al. 2019). Consequently, the 5-year survival rate remains low despite surgery, radiotherapy, and chemotherapy. In patients with penal SCC, lymph node involvement in the groin is the most important prognostic factor for survival (Novara, Galfano et al. 2007). Thus, the 5-year cancer specific survival for men with SCC of the penis is between 75-93% for those with node-negative disease that diminishes with lymph node involvement. Similarly, lymph node status is the most important prognostic factor for women with vulvar SCC (Gadducci, Tana et al. 2012). The 5-year survival rate is between 70-98% for vulvar SCC patients with negative nodes compared to only 12-41% with metastatic nodes. In Europe, the 5-year survival rate of patients with anal SCC varies between 44-66% depending on the region (Glynne-Jones, Nilsson et al. 2014). A study that assessed survival in 198 cervical SCC patients reported a median OS of 61.3 months and a 5-year OS rate of 56.6% (Li, Liu et al. 2016). The study also showed that the presence of lymph node metastasis is a negative prognostic factor for survival in cervical SCC patients.

1.1.3. *Investigational medicinal products (IMP)*

1.1.3.1. Pembrolizumab

Pembrolizumab is a highly selective humanized monoclonal antibody (mAb) that bind to the cell surface receptor programmed cell death protein 1 (PD-1) (du Rusquec, de Calbiac et al. 2019). This binding antagonizes the interaction between PD-1 and its ligands, PD-L1 and PD-L2: interrupting the down-modulation of T-cell immune response. Pembrolizumab is a human immunoglobulin G4/kappa isotype with a stabilizing sequence alteration in the Fc region.

Pembrolizumab is approved for treating melanoma, non-small cell lung cancer (NSCLC), Hodgkin lymphoma, urothelial carcinoma, H&N SCC, and renal cell carcinoma (du Rusquec, de Calbiac et al. 2019). Pembrolizumab is predominantly used for patients with metastatic disease or in cases were available treatments have failed. For treating renal cell carcinoma, it is approved in combination with other treatments. In NSCLC and H&N SCC is can be used as monotherapy or in combination. In the other cancer mention, pembrolizumab is only approved as monotherapy.

Refer to the current version of the pembrolizumab investigator brochure (IB) for a complete summary of non-clinical and clinical data including safety, efficacy, and pharmacokinetics data.

1.1.3.2. Vorinostat

Vorinostat is a synthetic hydroxamic acid derivative that inhibits histone deacetylase (HDAC). by binding to the catalytic domain of the HDAC (Laengle, Kabiljo et al. 2020).

Small molecule inhibitors of HDAC are a new class of antineoplastic agents. They have antiproliferative effects, such as inducing cell differentiation, cell-cycle growth arrest, and apoptosis (Duvic and Vu 2007). HDAC inhibitors, such as vorinostat, act mainly by preventing deacetylation resulting in the accumulation of both hyperacetylated histones and transcription factors. Vorinostat direct effect the regulation of numerous genes, ultimately resulting in a decrease in the proliferation of malignant cells. Vorinostat is approved for the treatment of cutaneous manifestations in patients with cutaneous T-cell lymphoma who have progressive, persistent, or recurrent disease on or following two systemic therapies.

Refer to the current version of the vorinostat SmPC for a complete summary of non-clinical and clinical data including safety, efficacy, and pharmacokinetics data.

1.2. Trial rationale

Traditionally, cancer treatments have been approved according to their location. However, now with a deeper understanding of the genetic and molecular mechanism of cancer, this paradigm is being questioned. Indeed, the FDA has recently approved pembrolizumab for tumours with high microsatellite instability (MSI) and larotrectinib for neurotrophic receptor tyrosine kinase (NRTK) fusion tumours. These agnostic therapies target underlying biological mechanism common to several tumours. Indeed, SCCs have traditional been treated according to their location, despite sharing genetic, genomic, and epigenetic modifications. Consequently, new agnostic therapies have and are being developed to target these common modifications.

1.2.1. *Immunotherapy for treating SCC*

Immunotherapy has already proven to be an effective treatment for patients with advanced SCC (see Table 1). Indeed, several clinical trials have assessed immunotherapies, pembrolizumab and nivolumab, in several SCCs including H&N, anal, cervical, vulvar & vagina, and lung SCC. These immunotherapies show activity in several SCCs, with ORR ranging from 0 to 32%.

There is no strong evidence yet, that HPV status is correlated to anti-tumor activities of immunotherapies. In the study KEYNOTE-055, ORR observed in H&N patients treated with pembrolizumab is similar in both HPV populations (HPV+:n=37, 16% ; HPV– n=131, 15%). Contradictorily, in the study KEYNOTE-012, ORR observed in H&N patient treated with pembrolizumab is more important in the HPV+ subgroup (HPV+ n=28, 32% ;HPV- n=104, 14%).

Table 1 - Immune checkpoint inhibitors evaluated for treating squamous cell carcinomas (SCC)

Trial name	Type of SCC	Immune checkpoint inhibitor	Lines of prior treatment	Patient selection	N	Phase	ORR (%)	References
Keynote-040	H&N	pembrolizumab	1-3	-	247	III	15	(Cohen, Soulieres et al. 2019)
Checkmate 141	H&N	nivolumab	Any	-	240	III	13	(Ferris, Blumenschein et al. 2016)
Keynote-055	H&N	pembrolizumab	Any	-<br>HPV+<br>HPV-<br>CPS>1<br>CPS<1<br>CPS>50<br>CPS<50	171<br>37<br>131<br>140<br>26<br>48<br>118	II	16<br>16<br>15<br>18<br>12<br>27<br>13	(Bauml, Seiwert et al. 2017)
Keynote-012 (Expansion cohorts)	H&N	pembrolizumab	Any	-<br>CPS>1<br>CPS<1<br>HPV+<br>HPV-<br>TPS<1%<br>TPS>1%	132<br>107<br>25<br>28<br>104<br>43<br>89	I	18<br>22<br>4<br>32<br>14<br>16<br>19	(Chow, Haddad et al. 2016)

Trial name	Type of SCC	Immune checkpoint inhibitor	Lines of prior treatment	Patient selection	N	Phase	ORR (%)	References
Keynote-012	H&N	pembrolizumab	Any	PD-L1>1%<br>HPV+<br>HPV-	45<br>16<br>29	I	18<br>25<br>14	(Seiwert, Burtness et al. 2016)
NCI9673	Anal	nivolumab	>2	-	37	II	24	(Morris, Salem et al. 2017)
Keynote-028	Anal	pembrolizumab	Any	PD-L1>1	25	I	16	(Ott, Bang et al. 2019)
Keynote-028	Cervical	pembrolizumab	Any	PD-L1>1	24	I	17	(Frenel, Le Tourneau et al. 2017)
Checkmate 358	Cervical	nivolumab	1-3	-	19	I/II	26	(Naumann, Hollebecque et al. 2019)
Keynote-028	Vulvar	pembrolizumab	Any	PD-L1>1	18	I	6	(Ott, Bang et al. 2019)
Checkmate 358	Vulvar/Vagina	nivolumab	1-3	-	5	I/II	0	(Naumann, Hollebecque et al. 2019)
Checkmate 017	Lung	nivolumab	Any	-	135	III	20	(Brahmer, Reckamp et al. 2015)

1.2.2. *Immune checkpoint inhibitors combined with HDAC inhibitors for treating SCC*

We have seen that immunotherapies as monotherapy have shown anti-cancer activity in several SCCs. However, several studies have shown that HDAC inhibitors potentiate immunotherapy (Mazzone, Zwergel et al. 2017). Indeed, a recent preclinical study reported enhanced efficacy in combining an immune checkpoint inhibitor with an HDAC inhibitor (belinostat) in murine hepatocellular carcinoma models (Llopiz, Ruiz et al. 2019). Similar results were reported in lung and renal cell carcinomas murine tumour models. Indeed, entinostat a HDAC inhibitor potentiated PD-1 inhibit in these models (Orillion, Hashimoto et al. 2017). Therefore, preclinical studies provide rationale for combining an HDAC inhibitor with an immune checkpoint inhibitor.

This combination is currently being assessed in several ongoing early phase trials. These include pembrolizumab combined with vorinostat in advanced renal or urothelial cell carcinoma patients (NCT02619253), pembrolizumab combined with entinostat in metastatic uveal melanoma patients (NCT02697630) (Jespersen, Olofsson Bagge et al. 2019), nivolumab combined with azacitidine and entinostat in non-small cell lung cancer patients (NCT01928576), and aldesleukin (interleukin-2) combined with entinostat in metastatic kidney cancer patients (NCT01038778).

1.2.3. *Pembrolizumab combined with vorinostat for treating SCC*

In this study, we would like to assess vorinostat combined with pembrolizumab for treating SCCs at various locations. This combination (vorinostat and pembrolizumab) has already been evaluated in SCCs and results have recently been published (Gray, Saltos et al. 2019, Rodriguez, Wu et al. 2020). Firstly, the results of a phase I/Ib study assessing pembrolizumab combined with vorinostat for treating patients with advanced/metastatic NSCLC have recently been published (Gray, Saltos et al. 2019). In the 24 patients evaluable for efficacy: 3 patients with PR, 11 with SD, and 10 with PD. The disease control rate (patients with PR and SD) was 58%. In addition, in the study population (n=33), median OS was 18.2 months and median PFS was 15.3 months.

Secondly, Rodriguez et.al. assessed the pembrolizumab-vorinostat combination in patients with recurrent metastatic head and neck SCC and salivary gland cancers(Rodriguez, Wu et al. 2020). The study enrolled 25 patients with H&N SCC. After a median follow-up of 12.6 months, the median OS was 12.6 months and the median PFS was 4.5 months. Of the 25 patients, 8 patients had a PR, 4 a SD, and 10 a PD. The median duration of response was 6.2 months and the ORR was 32%.

Therefore, we designed the PEVOsq trial to evaluate the efficacy of pembrolizumab combined with vorinostat in patients with recurrent and/or metastatic SCC in various anatomical locations. Furthermore, we planned to collect and analyze biological samples to identify potential biomarkers of efficacy and resistance.

1.3. Justification for the therapeutic regimens and treatment durations

Pembrolizumab efficacy is driven by the reactivation of adaptive immune response by blocking PD-1 expressed on T cells and not through the direct binding to cancer cells. Once the PD-1 on T cells are saturated through binding with pembrolizumab, efficacy is not expected to increase with further exposure. It has been shown that exposure at 2 mg/kg every 3 weeks (Q3W) is similar to that at 200 mg Q3W (Freshwater, Kondic et al. 2017). In addition, PK results in participants with various tumours: melanoma, NSCLC, HNSCC, and MSI-H show no meaningful difference in PK of pembrolizumab. Therefore, a pembrolizumab dosage of 200 mg Q3W is considered appropriate for treating multiple indications.

The HDAC inhibition in PBMC following vorinostat administered ration at 400 mg dose level lasted at least 10 hours. The pharmacokinetics PK of vorinostat after multiple dose administrations is qualitatively similar to that observed after a single dose. When vorinostat was taken at 400 mg once daily with food for 28twenty-eight consecutive days, concentration time profiles were qualitatively similar on days 1 and 28, indicating no obvious changes in drug absorption or elimination.

Vorinostat reaches its maximum serum concentration within 15 min to 6 h after intake. The drug is rapidly eliminated with a half-life of between 60-100 min (Bubna 2015). In addition, vorinostat is not metabolized, nor does it inhibit, cytochrome P-450 isoenzyme. Phase I and II studies have shown that 400 mg of vorinostat, once daily, is effective with acceptable toxicity in patients with various cancers. Refer to the current version of the vorinostat SmPC for additional information.

In the trial assessing the combination for treating H&N SCC patients, pembrolizumab (200 mg) was administered every 21 days, and vorinostat (400mg) taken 5 days on and 2 days off during each 21-day cycle (Rodriguez, Wu et al. 2020). However, the dose-escalation study assessing the combination gave pembrolizumab (200 mg) Q3W and oral vorinostat daily at either 200 mg (dose

level 1) and 400 mg (dose level 2) (Gray, Saltos et al. 2019). No dose-limiting toxicities were observed and the recommended phase II dosage of pembrolizumab was 200 mg Q3W with daily vorinostat (400 mg) during every 21-day cycle. This dosage was reportedly well tolerated. Therefore, the dosage schedule that will be used in this trial is intravenous pembrolizumab, 200 mg Q3W combined with oral vorinostat, 400 mg once daily.

1.4. Potential risks and benefits

1.4.1. *Known potential risks*

1.4.1.1. Pembrolizumab

The safety profile of pembrolizumab is based on the cumulative safety dataset which is a pools safety data summary of adverse events (AE) from several clinical studies.

The most frequently reported (≥10%) AE are fatigue, nausea, diarrhea, decreased appetite, cough, pruritus, constipation, dyspnea, arthralgia, rash, vomiting, pyrexia, anemia, headache, asthenia, back pain, and abdominal pain. The most frequently reported (≥1%) serious adverse events (SAE) are pneumonia, pneumonitis, pleural effusion, dyspnea, pyrexia, pulmonary embolism, anemia, and colitis. The most frequently reported (≥10%) drug-related AE are fatigue, pruritus, rash, diarrhea, and nausea. In addition, the most frequent (≥0.5%) drug-related SAEs reported were pneumonitis, colitis, and diarrhea.

Pneumonitis is the most common event leading to discontinuation of pembrolizumab therapy. The frequency of drug-related deaths remains very low, pneumonitis being most common event leading to death. Immune-mediated adverse events and infusion-related reactions maybe associated with Pembrolizumab use.

Refer to the current version of the pembrolizumab investigator brochure (IB) for a complete summary of non-clinical and clinical safety data.

1.4.1.2. Vorinostat

The maximum tolerated dose for continuous daily dosing without a rest period is 400 mg daily. Most dose limiting toxicities occurred within the first month of treatment with oral vorinostat, and resolved quickly once treatment is interrupted.

The most frequently reported (≥10%) AE for patients treated with vorinostat, either as monotherapy or in combination, are nausea, diarrhea, fatigue, vomiting, thrombocytopenia, anemia, decreased appetite, constipation, neutropenia, dyspnea, pyrexia, cough, weight decreased, asthenia, blood creatinine increased, anorexia, hyperglycemia, leukopenia, headache, dizziness, dysguesia, alopecia, abdominal pain, hypokalemia, and back pain. In addition, the most frequently (≥1%) drug-related SAE are thrombocytopenia, diarrhea, dehydration, vomiting, febrile neutropenia, anemia, nausea, and pneumonia. Fatigue is the most common event leading to treatment discontinuation.

Refer to the current version of the vorinostat SmPC for a complete summary of non-clinical and clinical safety data.

1.4.1.3. Safety profile of the pembrolizumab combined with vorinostat

In the phase I/Ib study assessing the pembrolizumab-vorinostat combination for treating patients with advanced/metastatic NSCLC safety was assessed in 33 patients (Gray, Saltos et al. 2019). No

dose-limiting toxicities nor treatment-related deaths were reported. The most common AEs noted were fatigue (33% of patients), nausea (27%), and vomiting (27%). The only immune-related AE reported in ≥5% of patients was hypothyroidism: reported in 15% of patients. Although, irAEs of increases in liver enzyme levels (ASAT/ALAT), arthralgia, colitis, diarrhea, and myalgia were all reported in about 3% of patients. In addition, in the study assessed the combination in patients with recurrent metastatic H&N SCC and salivary gland cancer 1 of 3 patients with H&N SCC reported fatigue of grade 3: considered as a dose-limiting toxicity during the safety run-in period (Rodriguez, Wu et al. 2020). Furthermore, AEs (all grades) were reported in 14 patients (56%) and those of grade ≥3 in 9 patients (36%). Pembrolizumab combined with vorinostat was reported to be well-tolerated (Gray, Saltos et al. 2019).

1.4.2. *Known potential benefits*

As discussed above, two clinical trials have assessed the efficacy and safety of pembrolizumab combined with vorinostat in patients with H&N SCC and in NSCLC. Gray et al. assessed the combination in NSCLC patients in a phase I/Ib study (Gray, Saltos et al. 2019). The disease control rate (patients with PR and SD) was 58%. In addition, median OS was 18.2 months and median PFS was 15.3 months. The single-institution, phase II study assessed the combination in patients with H&N SCC and salivary gland cancer (Rodriguez, Wu et al. 2020). In H&N SCC patients (n=25), 8 patients had a PR, 4 a SD, and 10 had disease progression: DCR was 48%. In addition, median OS was 12.6 months and median PFS was 4.5 months.

Based on these data, we envisage that the combination of pembrolizumab plus vorinostat will be effective to treat patients with advanced SCCs of different locations.

1.5. Trial population

The trial population will be composed of men and women, aged ≥18 years, with a histologically confirmed progressive late-stage squamous cell carcinoma of the head and neck, cervix, lung, anus, vulva/vagina, or penis.

Squamous cell cancers located in the vulva/vagina, penis, cervix or anus have no standard of care established in the recurrent and/or the metastatic setting. Hence the combination of pembrolizumab and vorinostat can be proposed as a 1st line strategy in this setting.

For head and neck cancer, EMA has recently approved pembrolizumab in 1st line in metastatic setting, but approval is pending in France. For those patients, the study combination provides an anticipated access to immune checkpoint inhibitor.

For lung cancer, pembrolizumab is already the standard of care in 1st line metastatic setting with or without chemotherapy. Proposing a combination could bring an additional benefit, which will be evaluated in the PEVOsq trial.

Patient treatment-naive in recurrent and/or metastatic setting can be therefore included in study and treated by the combination of pembrolizumab and vorinostat.

From a pathological and anatomical point of view, vulvar and vaginal share the same clinical, pathological and biological characteristics. Both cancer types are most of time related to HPV infection. In the clinic, both cancer types are treated the same way, not only in the local or locally advanced setting, but also in the recurrent and/or metastatic setting with the same anticancer agents. Given the rarity of vulvar cancer and the rarity of vaginal cancer, it makes sense to us to reunite vaginal squamous cell carcinoma patients to the vulvar squamous cell carcinoma patient in the same cohort.

2. TRIAL OBJECTIVES

2.1. Primary objective

The primary objective of this trial is to evaluate the antitumor activity of pembrolizumab in combination with vorinostat in patients with recurrent and/or metastatic squamous cell carcinoma of the head and neck, cervix, lung, anus, vulva/vagina, and penis, using the ORR during treatment (investigator assessment).

2.2. Secondary objectives

The secondary objectives of this clinical study are:

- To determine, in each cohort, the anti-tumor activity in term of:
  - Centrally confirmed objective response rate (ORR), as per RECIST v1.1.
  - Centrally confirmed immune objective response rate (iORR), as per iRECIST.
  - Duration of response (DOR).
  - Progression-free survival (PFS), as per RECIST.
  - Immune-progression-free survival (iPFS), as per iRECIST.
  - Overall survival (OS).
- To evaluate the safety and tolerability of pembrolizumab in combination with vorinostat according to NCI CTCAE v5.0:
  - In each cohort.
  - In the overall study population.

2.3. Translational objectives

The translational studies associated with this clinical study aim to assess the following:

- Link between the tumor molecular profile and immune parameters.
- Link between immune-related biomarkers and drug combination efficacy.
- Post-treatment modification of immune-related and molecular epigenetic biomarkers.
- Predictive value of tumor microenvironment and epigenetic parameters.
- Predictive value of circulating biomarkers.

3. TRIAL DESIGN AND ENDPOINTS

3.1. Description of the trial Design

This is an open-label, non-randomized, multi-center, basket phase II trial evaluating the efficacy of pembrolizumab in combination with vorinostat in adult patients with recurrent and/or metastatic squamous cell carcinoma of different locations.

Figure 1 - Study design

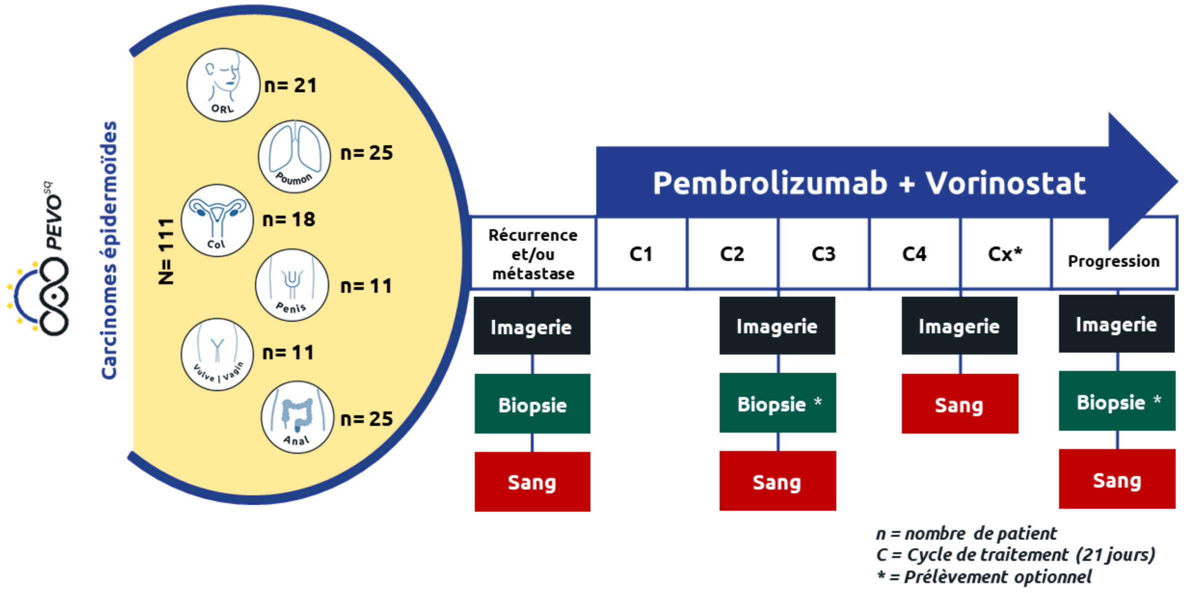

3.2. Trial Endpoints

3.2.1. *Primary endpoint*

Investigators will assess the ORR. The ORR is defined in each cohort as the percentage of evaluable patients for ORR, designate as the proportion of patients with best response of complete response (CR) or a partial response (PR) during treatment according to RECIST v1.1. (Appendix 6).

3.2.2. *Secondary endpoint(s)*

- Anti-tumor activity endpoints will be evaluated in each cohort:
  - ➢ ORR defined as the proportion of patients with best response of CR or PR during treatment, as assessed by a central radiological panel according to RECIST v1.1 (Appendix 6).
  - ➢ iORR defined as the proportion of patients with best response of CR or PR during treatment, as assessed by a central radiological panel according to immune-specific response criteria (iRECIST, Appendix 7).
  - ➢ DOR will be evaluated in patients with either a complete response (CR) or partial response (PR). DOR is defined as the time from the first assessment of a CR or PR until the date of

the first occurrence of progressive disease (PD) or death from any cause (if death occurred within predefined period), whichever occurs first.

- PFS is defined per RECIST1.1 as the time from inclusion until disease progression (per RECIST v1.1) or death from any cause, whichever occurs first. At the time of analysis, a patient alive and without disease progression will be censored at the date of the last tumor assessment. Patients alive without disease progression who started a new anticancer therapy will be censored at the date of the last tumor assessment prior to the start of the new anticancer therapy.
- iPFS is defined per iRECIST as the time from inclusion until confirmed disease progression (per iRECIST), or death from any cause, whichever occurs first. At the time of analysis, a patient alive and without progression will be censored at the date of the last tumor assessment. Patients alive without disease progression who started a new anti-cancer therapy will be censored at the date of the last tumor assessment prior to the start of the new anticancer therapy.
- OS is defined as the time from inclusion until death from any cause. Patients who are alive at last follow-up news will be censored at this date.

- The safety will be evaluated according to the incidence of adverse events (AEs) graded by NCI-CTCAE v5.0 (Appendix 8):
  - In each cohort.
  - In the overall study population.

3.3. Translational endpoints

- To assess the link between the tumor molecular profile including epigenetics features and immune parameters of the tumor microenvironment (TME) and in blood samples.
- To assess the link between immune-related biomarkers in the TME and in blood samples (including but not limited to tumor tissue PD-L1 expression by IHC, RNA gene expression profiling, and DNA mutation analysis), and measures of efficacy.
- To explore the modification of immune-related and molecular epigenetic biomarkers following treatment.
- To analyze the impact of TME and epigenetics parameters on sensitivity/response to treatment.
- To assess the predictive value of circulating biomarkers on sensitivity or resistance to treatment.

3.4. Progression of the trial

Patients participating in the trial will comply with the protocol for approximately 2 years after they are included, including 1 year of follow-up after treatment termination.

The end of the study corresponds to the last visit of the last patient (i.e. last follow-up visit).

The investigation/examination schedule is defined by the trial schedule of activities in Section "Schedule of visits and activities" of the protocol summary.

3.5. Inclusion procedure

After the eligibility criteria have been fulfilled and the patient consent has been obtained, the patient will be registered in the study using the registration form on R&D UNICANCER's online electronic case report form (eCRF) website (Ennov Clinical® software):

https://ecrf.icm.unicancer.fr/CSOnline/

Once the patient has been registered, automatic e-mails confirming registration will be sent to the:

- sponsor
- investigator
- data manager
- pharmacist

The eCRF registration procedure will be explained to the center staff during the study initiation visit. The corresponding documents, with explanation of the procedure, will be given to the principal investigator of each center and filed in the Site Master File (SMF).

3.6. Premature Trial Terminations and Suspension

The trial can be suspended or stopped by the sponsor after meeting with the coordinating investigator or following a request by the respective regulatory authority and/or the responsible Ethics Committee for the following reasons:

- High frequency and/or unexpected severity of toxicity
- Insufficient patient enrolment
- Insufficient quality of data collection

3.7. Patient's trial withdrawal and discontinuation

Patient withdrawal concerns patients who stop treatment and all other protocol-defined procedures. Please note that treatment discontinuation without consent withdrawal from a patient is not considered as a trial withdrawal. For treatment discontinuation only, please refer to Section 5.7.

This can occur under the following circumstances:

- Patient withdraws consent
- The principal investigator may terminate a patient's participants from the trial, if this is in the interest of the patient.

Trial patients may withdraw their consent at any time without justification, irrespective of the reason(s). In the case of trial withdrawal the investigator should attempt to obtain as much information as possible. This information should be noted in the patient's medical file. The patient's withdrawal of consent does not impact the patient's right to receive the standard of care medical treatment.

4. PATIENT SELECTION

4.1. Diagnosis and inclusion criteria

The following criteria must be verified before inclusion. In order to participate in the study all patients must meet all of the following inclusion criteria:

1. Aged ≥18 years old.
2. Patients with Eastern Cooperative Oncology Group (ECOG) performance status ≤1 (Appendix 1).
3. Patients must have histologically confirmed recurrent and/or metastatic squamous cell carcinoma of the head and neck, cervix, lung, anus, vulva/vagina, or penis.
4. Patients must have radiologically confirmed progressive recurrent and/or metastatic disease.
5. Patients naive or previously treated for their recurrent and/or metastatic disease.
6. Disease amenable to biopsy for study purpose.
7. Measurable disease according to RECIST v1.1.
8. Adequate renal function: serum creatinine ≤1.5 x upper limit of normal (ULN) (OR creatinine clearance [Cockcroft and Gault] ≥30 mL/min for participant with creatinine levels >1.5 × ULN) within 14 days prior inclusion.
9. Adequate liver function: aspartate aminotransferase (AST) and alanine aminotransferase (ALT) levels ≤2.5 × ULN (≤5 ULN when documented liver metastases) and total bilirubin level ≤1.5 × ULN, within 14 days prior inclusion.
10. Adequate bone marrow function: absolute neutrophil count (ANC) ≥1,500/mm³, platelet count ≥100,000/mm³, and hemoglobin ≥9 g/dL, within 14 days prior inclusion.
11. Adequate coagulation: prothrombin time (PT)/international normalized ratio (INR) ≤1.5 × ULN within 14 days prior inclusion If participant is receiving anticoagulant therapy then the PT or activated partial thromboplastin time (aPTT) should be within the therapeutic range of intended use of anticoagulant.
12. Female of child-bearing potential must have a negative serum pregnancy test within 72 h before starting study treatment.
13. Female of childbearing potential, must use "highly effective" methods of contraception for the study duration and for 4 months following the last dose of pembrolizumab and 6 months following the last dose of vorinostat.
14. Male participants must agree to use an effective contraceptive for the duration of the trial and for at least 4 months after the last the last dose of pembrolizumab and 6 months following the last dose of vorinostat (to allow for effective elimination of the study drugs). Also, they should refrain from donating sperm during this period.
15. Patients must be willing and able to comply with the protocol for the duration of the study including scheduled visits, treatment plan, and laboratory tests.
16. Patients must be willing and able to comply with other study procedures, including a baseline tumor biopsy and a series of blood samples throughout the study.
17. Patients able to swallow oral medications.

18. Patients must be affiliated to a Social Security System (or equivalent).
19. Patients must have signed a written informed consent prior to any trial-specific procedures. When the patient is physically unable to give their written consent, a trusted person of their choice, independent from the investigator or the sponsor, can confirm in writing the patient's consent.

4.2. Non-inclusion criteria

Patients are not eligible to participate in the trial if they comply with any of the following criteria:

1. Prior treatment with anti-PD-1/PD-L1 agents or histone deacetylases (HDAC) inhibitors.
2. Patients with central nervous system involvement that has not been controlled for >3 months.
3. Patients with no other site for biopsy than bone lesions.
4. Patients with other concurrent severe and/or uncontrolled medical disease which could compromise participation in the study, including uncontrolled diabetes, cardiac disease, uncontrolled hypertension, congestive cardiac failure, ventricular arrhythmias, active ischemic heart disease, myocardial infection within one year, chronic liver or renal disease, active gastrointestinal tract ulceration, severely impaired lung function.
5. Known history of human immunodeficiency virus (HIV), Hepatitis B virus (HBV; defined as Hepatitis B surface antigen [HBsAg] reactive) or known active Hepatitis C virus (HCV; defined as HCV RNA detected) virus infection.
6. History of autoimmune disease with the exception of:
   - (1) Patients with a history of autoimmune hypothyroidism on a stable dose of thyroid replacement hormone,
   - (2) Patients with controlled Type 1 diabetes mellitus on a stable insulin regimen,
   - (3) Patients with eczema, psoriasis, lichen simplex chronicus, or vitiligo with dermatologic manifestations only (e.g., patients with psoriatic arthritis would be excluded) provided that they meet the following conditions: (i) Rash must cover less than 10% of body surface area; (ii) Disease is well controlled at baseline and only requiring low potency topical steroids; (iii) No acute exacerbations of underlying condition within the previous 12 months (not requiring psoralen plus ultraviolet A radiation [PUVA], methotrexate, retinoid, biologic agents, oral calcineurin inhibitors, high-potency or oral steroids).
7. History of allogeneic organ or bone marrow transplantation.
8. History of non-infectious pneumonitis that required steroids or has current pneumonitis.
9. Has an active infection requiring systemic therapy.
10. Has received a live vaccine within 30 days prior to the first dose of study drug. Examples of live vaccines include, but are not limited to, the following: measles, mumps, rubella, varicella/zoster (chicken pox), yellow fever, rabies, Bacillus Calmette–Guérin (BCG), and typhoid vaccine. Seasonal influenza vaccines for injection are generally killed virus vaccines and are allowed; however, intranasal influenza vaccines (e.g, FluMist®) are live attenuated vaccines and are not allowed.

11. Known prior severe hypersensitivity to investigational products or its excipients,
12. Has received prior systemic anti-cancer therapy including investigational agents within 4 weeks [could consider shorter interval for kinase inhibitors or other short half-life drugs] prior to first dose of study treatments.

    Note: Participants must have recovered from all AEs due to previous therapies to ≤Grade 1 or baseline. Participants with ≤Grade 2 neuropathy may be eligible.
13. Has received prior radiotherapy within 2 weeks of start of study treatment. Participants must have recovered from all radiation-related toxicities, not require corticosteroids, and not have had radiation pneumonitis. A 1-week washout is permitted for palliative radiation (≤2 weeks of radiotherapy) to non-CNS disease.
14. Major surgery within 28 days prior to the first dose of study treatments.

    Note: Local surgery of isolated lesions for palliative intent is acceptable.
15. Current or prior use of immunosuppressive medication within 7 days before the first dose of pembrolizumab. The following are exceptions to this criterion:
    - Intranasal, inhaled, topical steroids, or local steroid injections (e.g., intra articular injection),
    - Systemic corticosteroids at physiologic doses ≤10 mg/day of prednisone or its equivalent (see Appendix 5),
    - Steroids as premedication for hypersensitivity reactions (e.g., CT scan premedication).
16. Patients using drugs that could have pharmacokinetics interaction with investigational drugs. This includes, but is not limited to, valproic acid, coumarin-derivative anticoagulants, drugs that disrupt electrolyte levels, drugs that may prolong QT (see Appendix 4).
17. Pregnant women or women who are breast-feeding.
18. Patients enrolled in another therapeutic study within 30 days prior to inclusion and during the treatment period. Patients can participate in an independent approved non-interventional studies.
19. Patients unwilling or unable to comply with the medical follow-up required by the trial because of geographic, familial, social, or psychological reasons.
20. Persons deprived of their liberty or under protective custody or guardianship.

5. TRIAL TREATMENTS/INTERVENTIONS

5.1. Description of trial treatments/interventions

Patients having signed the informed consent form and successfully registered in the study (i.e. included) will receive concomitant pembrolizumab and vorinostat within 7 days of registration.

5.1.1. *Pembrolizumab*

Patients will be administrated pembrolizumab by intravenous injection of 200 mg on day1 of each 21-day cycles. Sites should make every effort to target infusion timing to be as close to 30 min as possible. However, given the variability of infusion pumps from site to site, a window of -5 min and +10 min is permitted (i.e., infusion time is 30 min: -5 min/+10 min).

Refer to Section 5.5.1 for specific instructions for the preparation of the pembrolizumab infusion fluid.

Pembrolizumab will be given up to 35 administrations (approximately 2 years).

Continuation of pembrolizumab beyond 35 cycles may be allowed under certain conditions and must be agreed with the sponsor before continuing the treatment.

5.1.2. *Vorinostat*

From day 1 of the Cycle 1, patients will take vorinostat orally once daily at a dose of 400 mg . Vorinostat should be taken approximately within 30 min of a meal food. Patients should be instructed to drink at least 2 liters/day of fluids for adequate hydration.

Patients are eligible for repeated treatment cycles in the absence of disease progression and undue adverse events.

5.2. Acquisition, reception, and storage

The investigational products pembrolizumab and vorinostat provided by Merck Sharp and Dohme (MSD) will be distributed to the pharmacy at the investigational center via a dedicated courier in accordance with the current Good Distribution Practices guidelines.

The pharmacist of the trial site will receive numbered treatment and will acknowledge receipt of all deliveries by sending the necessary completed documents to the distributor.

The pharmacist is responsible for a safe and proper handling and storage of the investigational medicinal products at the investigational center. The investigational products must be stored in a locked facility with restricted access to the pharmacist and authorized personnel, and under environmental conditions consistent with the drug manufacturer recommendations (IB).

- Pembrolizumab must be stored under refrigerated conditions (2°C to 8°C).
- Vorinostat must be stored at 2-30°C

Up to date temperature logs must be maintained by the pharmacist/investigator to document adequate storage during the trial. These logs must be available at the site during monitoring visits, and in the event of an audit or inspection.

If the storage conditions as indicated above are exceeded (e.g. temperature excursion) the pharmacist/investigator must place the corresponding treatments in quarantine and immediate notify

the sponsor who will indicate the procedure to follow. Under no circumstances should these treatments be delivered to trial patients without prior authorization by the sponsor.

5.3. Trial treatments accountability, return and destruction

The investigator/pharmacist must ensure that the investigational product is administrated only to patients enrolled in this trial. The investigational product must not to be used outside the context of the trial protocol.

The pharmacist or authorized staff must document the receipt, dispensation, and return or destruction of all investigational products received during this trial. Records on investigational products delivery to the center, the inventory at the center, the use by each patient, and the return to the sponsor or destruction by the site must be implemented and maintained by the pharmacist or another appropriately trained individual at the investigational center. The following minimum information must be present: all relevant dates (delivery dates, dispensation, returns, and destruction), quantities, and investigational product batch numbers. Accountability forms will be provided by the sponsor to ensure trial treatment accountability.

The pharmacist will implement an accounting of medicinal products dispensed, used, unused, returned by the patients. The accountability of the products returned by patients will be systematically done by the pharmacist of the site. These records will be made available to the clinical research associate (CRA) mandated by the sponsor.

For vorinostat capsules, treatment observance will be recorded in the eCRF, and the patients will be asked to complete a personal booklet.

5.4. Formulation, appearance, packaging, and labelling

5.4.1. *Pembrolizumab*

Pembrolizumab for intra-venous infusion is supplied as a clear to opalescent liquid solution in type I glass vials intended for single use only. Each vial contains 100 mg of pembrolizumab. It is manufactured using the fully formulated pembrolizumab with L-histidine as a buffering agent, polysorbate 80 as a surfactant, and sucrose as a stabilizer/tonicity modifier. Pembrolizumab vial should be stored under refrigerated conditions (2°C to 8°C).

5.4.2. *Vorinostat*

Vorinostat is supplied for oral administration in white opaque gelatin capsules (size 3) with “568” over “100 mg” printed within the radial bar in black ink on the capsule body. Each capsule contains 100 mg of vorinostat and the following inactive ingredients: microcrystalline cellulose, sodium croscarmellose, and magnesium stearate. The capsules are supplied in HDPE high-density polyethylene (HDPE) bottles. Each bottle contains 90 capsules. Please refer to either the product labeling or the shipping documentation to check the shelf life of vorinostat. Vorinostat capsules should be stored at room temperature (do not store bellow 2°C and above 30°C).

5.5. Preparation

5.5.1. *Pembrolizumab*

Pembrolizumab can be diluted with normal saline or 5% dextrose in the concentration range of 1 to 10 mg/mL in intra-venous containers made of PVC or non-PVC material. Reconstituted vials should be used immediately to prepare the infusion solution in the intra-venous bag, and the infusion solution should be administered immediately. If the diluted pembrolizumab solution is not used immediately, it may be stored for no more than 24 h at 2°C to 8°C. This 24-h hold time from reconstitution may include up to 6 hours at room temperature (at or below 25°C). Any additional hold time must be at 2°C to 8°C. If refrigerated, the vials and/or intravenous bags must be allowed to come to room temperature prior to use.

5.5.2. *Vorinostat*

Vorinostat capsules should not be opened or crushed. Direct contact of the powder in vorinostat capsules with the skin or mucous membranes should be avoided. If such contact occurs, wash thoroughly as outlined in the references. Personnel should avoid exposure to crushed and/or broken capsules.

5.6. Dose adaptation

5.6.1. *Pembrolizumab*

AEs associated with pembrolizumab exposure may represent an immunologic etiology. These immune-related AEs (irAEs) may occur shortly after the first dose or several months after the last dose of pembrolizumab treatment and may affect more than on body system simultaneously. Therefore, early recognition and initiation of treatment is critical to reduce complications. Based on existing clinical study data, most irAEs were reversible and could be managed with interruptions of pembrolizumab, administration of corticosteroids and/or other supportive care. For suspected irAEs, ensure adequate evaluation to confirm etiology or exclude other causes. Additional procedures or tests such as bronchoscopy, endoscopy, skin biopsy may be included as part of the evaluation. Based on the severity of irAEs, withhold or permanently discontinue pembrolizumab and administer corticosteroids. Dose modification and toxicity management guidelines for irAEs associated with pembrolizumab are provided in Appendix 9 and Appendix 10.

5.6.2. *Vorinostat*

Vorinostat dose modification guidelines are outlined Appendix 11 and should be used for the management of treatment-related toxicities. Patients should be carefully instructed when any dose interruption occurs.

If a patient is intolerant to vorinostat, the dose may be reduced to 300 mg orally once daily with food. The dose may be further reduced to 300 mg once daily with food for 5 consecutive days each week, as necessary.

In patients with mild to moderate hepatic impairment (bilirubin 1 to 3 x ULN or AST greater than ULN), the starting dose can be of 300 mg orally once daily with food. As a general rule, if a dose reduction is necessary, the dose should be reduced stepwise as described in Table 2 and the subject should be monitored for 10 to 14 days at each dose level. If toxicity recurs during this monitoring

time, the drug may need to be interrupted and/or the dose further decreased with continued monitoring for an additional 10-14 days at each dose level, and so on.

The dose should not be re-escalated to an earlier dose level on improvement of an AE.

Table 2 - Summary table for vorinostat dose reductions

**Suggested Dose Reduction Steps for 400 mg Starting Dose**	
**Starting Dose**	**400 mg once daily**
Dose Level -1	300 mg once daily
Dose Level -2	300 mg 5 days on/2 days off

**Suggested Dose Reduction Steps for 300 mg Starting Dose**	
**Starting Dose**	**300 mg once daily**
Dose Level -1	300 mg 5 days on/2 days off

The management of toxicities may require a dose interruption until recovery. If recovery is not observed after a 2-week interruption period, resumption should be discussed with the sponsor before reinitiating treatment. Clinical benefit and management options will be discussed with the physician.

5.7. Discontinuation of treatments:

Patients can discontinue the trial treatments for the following reasons:

- The participant or participant’s legally acceptable representative requests to discontinue study treatment but accept to continue with protocol.
- Confirmed radiographic disease progression.
- Any progression or recurrence of any malignancy, or any occurrence of another malignancy that requires active treatment.
- Limiting toxicity.
- The participant has a confirmed positive serum pregnancy test.
- Recurrent grade 2 pneumonitis.
- The participant has a medical condition or personal circumstance which, in the opinion of the investigator and/or sponsor, placed the participant at unnecessary risk from continued administration of study treatment.

After discontinuing all trial treatments, further treatment is left to the physician’s discretion.

Patients who discontinue all trial treatments will continue with the trial and the protocol-defined procedures and follow-up, unless they specifically withdraw their consent and indicate that they do not want to perform any further trial-related visits or assessments (for patient withdrawals see Section 3.7).

5.8. Concomitant medications and therapies

All medications (including herbal preparations) and therapies taken by the patients or administered to the patients from the onset of trial and given in addition to the investigational products during the trial are considered as concomitant medications. Any concomitant medication(s) during the trial should be carefully documented in the hospital patient records and made available to the sponsor CRA during the monitoring visits.

5.8.1. *Authorized concomitant treatments*

The following treatments/therapies are authorized during the trial:

- Systemic corticosteroids may be used to treat immune-mediated adverse reactions concomitant with pembrolizumab. Other indications are limited to those described in Section 5.8.1
- Concomitant medications or treatments (e.g., acetaminophen or dexchlorpheniramine) deemed necessary to provide adequate prophylactic or supportive care, except for those medications identified as “prohibited,” as listed in Section 5.8.2 can be administered as prescribed by the Investigator.
- Best supportive care (including antibiotics, nutritional support, correction of metabolic disorders, optimal symptom control, and pain management [including palliative radiotherapy to non-target lesions, antiemetic, etc.]) should be used, when necessary, for all patients.
- Inactivated viruses, such as those in the influenza vaccine.

  Note: intranasal influenza vaccines (e.g., FluMist®) are live attenuated vaccines and are not allowed.
- Drugs that disrupt electrolyte levels (i.e. loop/thiazide diuretics, laxative, enemas, amphotericin B, high dose corticosteroids) need to be used with caution and closely monitored.

5.8.2. *Prohibited concomitant treatments*

The following treatments/therapies are prohibited during the trial:

- Other antitumor treatments, including chemotherapies, hormonal therapies, biological response modifiers, and targeted therapies.
- Other investigational therapeutic agents.
- Immunosuppressive drugs.
- Systemic glucocorticoids at a dose greater than prednisone 10 mg/day or equivalent (Appendix 5). The use of physiologic doses of corticosteroids may be approved after consultation with the Sponsor, except in case of an urgent indication (see Section 5.8.1) or as antiemetics. Their use will require the highest precaution in patients with diabetes.

- It is advised not to use the association of vitamin K antagonist (VKA) with chemotherapy. It is preferable to use heparin and therapeutic anticoagulation with low-molecular weight heparin. If VKA cannot be avoided, the rate of prothrombin must be checked more frequently and INR monitored.
- Drugs that may prolong QT (i.e., amiodarone, procainamide, sotalol, venlafaxine, amitriptyline, sunitinib, methadone, chloroquine, clarithromycin, haloperidol, fluconazole, moxifloxacin, domperidone, ondansetron, etc.), see Appendix 4.
- Pimozide (Orap®) and cisapride (Prepulsid®) are strictly contraindicated: they are associated with a major risk of ventricular rhythm disorder.
- Radiation therapy. Note: Radiation therapy to a symptomatic solitary lesion or to the brain may be allowed at the investigator's discretion.
- Live vaccines within 30 days prior to the first dose of study treatment and while participating in the study. Examples of live vaccines include, but are not limited to, the following: measles, mumps, rubella, varicella/zoster, yellow fever, rabies, Bacillus Calmette–Guérin (BCG) vaccin, and typhoid vaccine.
- Vorinostat should not be administered concomitantly with other HDAC inhibitors (e.g., valproic acid) as class-specific adverse reactions may be additive (ex: Grade 4 thrombocytopenia with associated gastrointestinal bleeding and anemia).

5.8.3. *Rescue medications and therapies/Treatment at disease progression*

At disease progression the treatment will be at the investigator's discretion.

5.8.4. *Contraception during the trial*

5.8.4.1. Definitions

Females of childbearing potential are those who are not surgically sterile (i.e., bilateral tubal ligation, bilateral oophorectomy, or complete hysterectomy) or post-menopausal (for the definition of post-menopausal see below).

Post-menopausal status is defined as:

- Women will be considered post-menopausal if they have been amenorrhea for 12 months without an alternative medical cause. The following age-specific requirements apply:
    - Women <50 years of age would be considered post-menopausal if they have been amenorrhea for 12 months or more following cessation of exogenous hormonal treatments and if they have luteinizing hormone and follicle-stimulating hormone levels in the post-menopausal range for the institution or underwent surgical sterilization (bilateral oophorectomy or hysterectomy).
    - Women ≥50 years of age would be considered post-menopausal if they have been amenorrhea for 12 months or more following cessation of all exogenous hormonal treatments, had radiation-induced menopause with last menses >1 year ago, had chemotherapy-induced menopause with last menses >1 year ago, or underwent surgical sterilization (bilateral oophorectomy, bilateral salpingectomy or hysterectomy).

5.8.4.2. *Acceptable contraception during the trial*

- **Female patients of child-bearing potential**

Females of childbearing potential who are sexually active with a non-sterilized male partner must use one or more highly effective method of contraception (See Table 3 below) during the trial and for 4 months after the last the last dose of pembrolizumab and 6 months following the last dose of vorinostat. Non-sterilized male partners of a female patient must use male condom plus spermicide throughout this period. Cessation of birth control after this point should be discussed with a responsible physician. Female patients should also refrain from breastfeeding throughout this period.

Table 3 Highly effective methods of contraception (<1% failure rate)

**Barrier/Intrauterine methods**	**Hormonal methods**
Copper T intrauterine device<br><br>Levonorgestrel-releasing intrauterine system (e.g., Mirena®)[a]	Etonogestrel implants: e.g., Implanon or Norplan<br><br>Intravaginal device: e.g., ethinylestradiol and etonogestrel<br><br>Medroxyprogesterone injection: e.g., Depo-Provera<br><br>Normal and low dose combined oral contraceptive pill<br><br>Norelgestromin/ethinylestradiol transdermal system<br><br>Cerazette (desogestrel)

[a] This is also considered as a hormonal method.

- **Male patients with a female partner of childbearing potential**

Male participants with female partners of childbearing potential are eligible to participate if they agree to one of the following during the trial and for 4 months after the last the last dose of pembrolizumab and 6 months following the last dose of vorinostat, defined time frame in section 4:

  - Be abstinent from penile-vaginal intercourse as their usual and preferred lifestyle (abstinent on a long term and persistent basis) and agree to remain abstinent
  - Use a male condom plus partner use of a contraceptive method with a failure rate of <1% per year as described in Table 4 when having penile-vaginal intercourse with a woman of childbearing potential who is not currently pregnant.

Note: Men with a pregnant or breastfeeding partner must agree to remain abstinent from penile-vaginal intercourse or use a male condom during each episode of penile penetration

Table 4 - Highly effective methods of contraception in male (<1% failure rate)

**Highly Effective Methods That Have Low User Dependency**<br>*Failure rate of <1% per year when used consistently and correctly.*
• **Vasectomized partner**

A vasectomized partner is a highly effective contraception method provided that the partner is the sole male sexual partner of the WOCBP and the absence of sperm has been confirmed. If not, an additional highly effective method of contraception should be used.
● **Sexual abstinence**<br>Sexual abstinence is considered a highly effective method only if defined as refraining from heterosexual intercourse during the entire period of risk associated with the study treatment. The reliability of sexual abstinence needs to be evaluated in relation to the duration of the study and the preferred and usual lifestyle of the participant.
Notes:<br>Use should be consistent with local regulations regarding the use of contraceptive methods for participants of clinical studies.<br><br>a) If locally required, in accordance with Clinical Trial Facilitation Group (CTFG) guidelines, acceptable contraceptive implants are limited to those which inhibit ovulation.<br>b) If hormonal contraception efficacy is potentially decreased due to interaction with study treatment, condoms must be used in addition to the hormonal contraception during the treatment period and for at least [X days, corresponding to time needed to eliminate study treatment plus 30 days for study treatments with genotoxic potential] after the last dose of study treatment.

6. EVALUATION OF TREATMENT ACTIVITY AND SAFETY

6.1. Activity evaluation

6.1.1. *Tumor imaging during the study*

As far as possible, radiological assessments have to be repeated in the same technical conditions (same machine, parameters …) and reviewed by the same radiologist.

The first on-study imaging assessment should be performed within 14 days from the date of study registration (i.e., inclusion). Subsequent tumor imaging and clinical assessments should be performed every 6 weeks (within 7 days prior CxD1) from the initiation of treatment for the first 12 months of treatment or until first disease progression (whichever comes first), and then every 12 weeks until disease progression, when applicable.

The scheduled dates for tumour evaluations are not modified by dose delays. The assessment dates are relative to the treatment initiation date.

Imaging should continue to be performed until disease progression is identified by the investigator.

Objective response should be confirmed by a repeat imaging assessment. Tumor imaging to confirm PR or CR should be performed at least 4 weeks after the first indication of a response is observed, as required with RECIST v1.1 (see Appendix 6). Participants will then return to regular scheduled imaging every 6 weeks, starting with the next scheduled imaging time point. Participants who receive additional imaging for confirmation do not need to undergo the next scheduled tumor imaging if it is less than 4 weeks later; tumor imaging may resume at the subsequent scheduled imaging time point.

When the Investigator identifies radiographic progression per RECIST v1.1, efforts should be made to verify radiologic PD. Treatment should continue until PD has been verified. Regardless of whether PD is verified, if the Investigator considers the participant has progressed, but elects to implement RECIST, the Investigator may assess for confirmation of progression by iRECIST (Appendix 7) at subsequent time points.

Figure 2 - Imaging and treatment for clinically stable participants after first radiologic evidence of PD assessed by the investigator

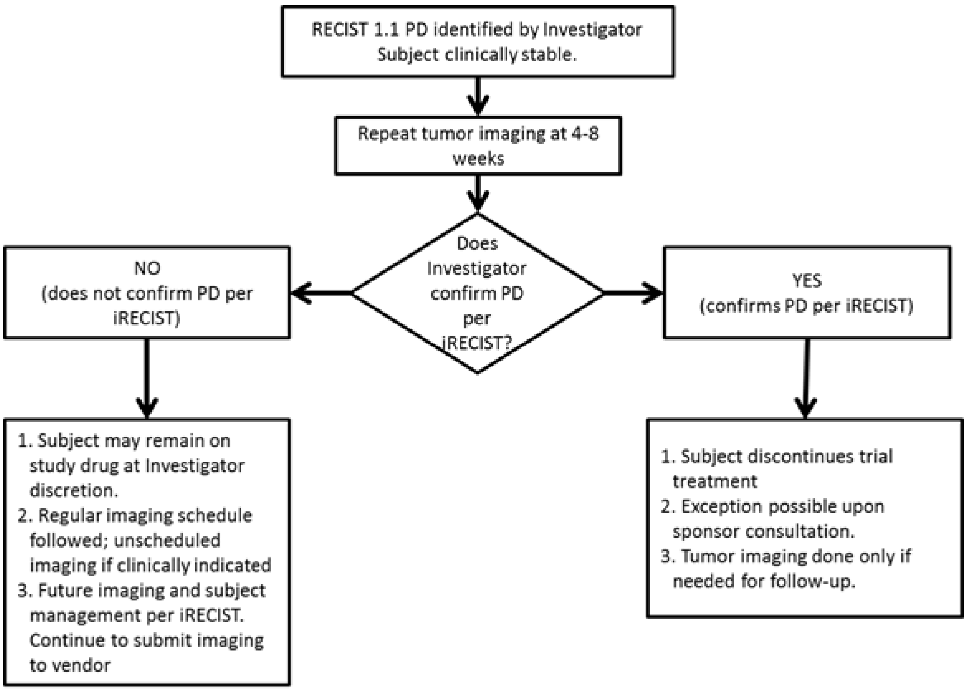

6.1.2. *End of treatment and follow-up tumor imaging*

In participants who discontinue all study treatments, tumor imaging should be performed at the time of treatment discontinuation (within 1 week). If previous imaging was obtained within 4 weeks prior to the date of discontinuation, then imaging at treatment discontinuation is not mandatory. In participants who discontinue study treatment due to documented disease progression and the Investigator elects not to implement iRECIST, this is the final required tumor imaging.

In participants who discontinue study treatment without documented disease progression, every effort should be made to continue monitoring their disease status by tumor imaging using the same imaging schedule used while on treatment (every 6 weeks in the first year and then every 12 weeks thereafter) to monitor disease status until the start of a new anticancer treatment, disease progression, pregnancy, death, withdrawal of consent, or the end of the study, whichever occurs first.

If pembrolizumab is permanently discontinued and vorinostat is continued, tumor imaging should be performed with the same imaging schedule. However, iRECIST assessment of the disease will not be necessary in this situation.

6.1.3. *RECIST v1.1 assessment of disease*

The primary objective is to evaluate the efficacy of the combined therapy (pembrolizumab and vorinostat) according to the ORR. The ORR is defined as the proportion of patients with best response of either a CR or PR during treatment. Treatment efficacy will be evaluated by the investigator by measuring changes in tumor size using contrast-enhanced CT-scan or MRI.

RECIST v1.1 (Appendix 6) will be used to assess tumor response, date of disease progression, and as a basis for all protocol guidelines related to disease status (e.g., discontinuation of study treatment). .

6.1.4. *iRECIST assessment of disease*

iRECIST is based on RECIST v1.1, but adapted to account for the unique tumor response seen with immunotherapeutic drugs. When clinically stable, participants should not be discontinued until disease progression is confirmed by the investigator, working with local radiology, according to the rules below. This means that treatment can be continued despite an initial radiological PD. This takes into account that certain participants will have a transient tumor flare in the first few months after the start of immunotherapy that does not reflect true tumor progression. Indeed, subsequent disease response have been reported after this initial pseudo-progression.

A description of the adaptations and iRECIST process is provided in Appendix 7, with additional detail in the iRECIST publication [Seymour et al, 2017].

iRECIST will be used by the investigator to assess tumor response and progression, and make treatment decisions.

6.1.5. *Blinded independent central review committee (BICR):*

An independent radiological review is planned at the end of the study. The BICR committee will review all the radiological tumor assessments according to modified RECIST v1.1 and iRECIST.

As a consequence, investigators must systematic upload a DICOM format record of the tumor assessment to the web platform subcontracted by Unicancer for each baseline and subsequent radiological tumor assessment (CT scan or MRI or other method) for all enrolled patients.

6.2. Safety evaluation

Safety will be assessed based on adverse event (AE) occurrence, the use of concomitant treatments, and changes occurring during treatment including those observed: during physical examinations, in vital signs (blood pressure, pulse, body temperature), and with biological and clinical examinations (biochemistry, hematology). Safety will be evaluated using the National Cancer Institute - common terminology criteria for adverse events (NCI-CTCAE) v5.0 (Appendix 8).

In case of emergency, the patient, a patient's relative, or the patient's general practitioner will be required to contact the investigator about AE that have occurred. The possible treatment interruption or dose adaptation (decrease) for the investigational product will be considered, as well as, adequate concomitant treatment if necessary.

It is anticipated that around 10% of patients treated with immune checkpoint inhibitors will develop severe, sometimes life-threatening dysimmune toxicities.

It is therefore necessary that the investigators rely on a previously identified local network of organ specialists and on detailed practical guidelines to anticipate, manage, and monitor appropriately immune related –Adverse Event (irAEs).

6.2.1. *Physical examination*

Physical examinations will be performed according to the assessment schedule. Full physical examinations will include assessments of the head, eyes, ears, nose, and throat, as well as the respiratory, cardiovascular, gastrointestinal, urogenital, musculoskeletal, neurological, dermatological, hematologic/lymphatic, and endocrine systems. Height will be measured at baseline only. Targeted physical examinations are to be utilised by the investigator on the basis of clinical observations and symptomatology.

Situations in which physical examination results should be reported as AEs are described in Section 8.

6.2.2. *Vital signs*

Vital signs (blood pressure [BP], pulse and temperature) and body weight will be evaluated according to the assessment schedules at each visit.

6.2.3. *Clinical laboratory tests*

Blood samples for clinical chemistry, haematology will be collected at the times indicated in the assessment schedules and as clinically indicated.

Clinical laboratory safety tests, including serum pregnancy tests, will be performed in a local licensed clinical laboratory. Abnormal clinically significant laboratory results should be repeated as soon as possible (preferably within 24 to 48 h).

Additional safety samples may be collected if clinically indicated at the discretion of the Investigator. The date, time of collection, and results (values, units, and reference ranges) will be recorded in the eCRF.

The laboratory variables to be measured are presented in Table 5.

Table 5 - Laboratory variables

Test Category	Test Name
Haematology	Hemoglobin, red blood cell count (RBC), white blood count (WBC), absolute neutrophil count (ANC), lymphocyte count, and platelet count.
Coagulation profile	Activated partial thromboplastin time (aPTT), international normalised ratio (INR), PT/INR
Clinical biochemistry	- Blood ionogram (Na, K, Ca, Mg, P, Cl, CO_2, total protein, albumin),<br>- Renal function (blood urea, creatinine),<br>- Hepatic function (direct bilirubin, alkaline phosphatase, ALT, AST, GGT),<br>- Thyroid hormone (TSH, free T3, free T4),<br>- Fasting plasma glucose, creatine phosphokinase,<br>- Squamous cell carcinoma antigen (SCCa).

7. DESCRIPTION OF VISITS AND INVESTIGATIONS

Patients will be monitored from the date of their screening visit until 1 year after the end of treatment or the date of death, withdrawal of consent, loss to follow-up, or end of study, whichever occurs first. A table summarizing the follow-up examination/visit schedule is provided in the section "Schedule of visits and activities" of the protocol summary.

7.1. Baseline visit

An informed consent form (ICF) must be signed before the evaluations required for inclusion and any study specific procedure are performed.

After signing the study ICF, baseline assessments will be done within 1 to 28 days prior to inclusion depending on assessments.

A procedure or evaluation already performed within the standard of care and which is in accordance with the protocol requirements does not need to be repeated unless clinically justified.

For laboratory evaluations used to determine eligibility, a repeated evaluation within the screening window is permitted for baseline results out of the defined range.

- **Patient history:** within 28 days of inclusion
  - Demographic data
  - Medical history and characteristics/prior therapy, (including HPV status, if available).
- **Physical examination:** within 14 days of inclusion
  - ✓ Patient's weight.
  - ✓ Complete clinical examination.
  - ✓ ECOG performance status (Appendix 1).
  - ✓ Vital signs (pulse rate, systolic and diastolic blood pressure, and body temperature).
  - ✓ Ongoing adverse events using the NCI-CTCAE v5.0 (Appendix 8).
- **Paraclinical examination:** within 14 days of inclusion
  - ✓ Radiologic assessment (RECIST [Appendix 6]): CT scan.
  - ✓ Electrocardiogram (12-lead ECG) : one measure
- **Biological tests:** within 14 days of inclusion (except pregnancy test)
  - ✓ Hematology: hemoglobin, red blood cell count, white blood cell count (WBC), absolute neutrophil count (ANC), lymphocyte count, and platelet count.
  - ✓ Blood ionogram: Na, K, Ca, Mg, P, Cl, CO_2, total protein, albumin.
  - ✓ Renal function: blood urea, creatinine.
  - ✓ Hepatic function: direct bilirubin, alkaline phosphatase, ALT, AST, GGT.
  - ✓ Thyroid hormone: TSH, free T3, free T4.
  - ✓ Coagulation profile: PT/INR, aPTT.

- ✓ Squamous cell carcinoma antigen (SCCa).
- ✓ Fasting plasma glucose, creatine phosphokinase.
- ✓ Serum pregnancy test (βHCG) **within 3 days (72 h) of inclusion**

- **Translational research:** within 14 days of inclusion (refer to section 9 for detailed sampling and approach):
  - ✓ Blood sample :
    - 2 x 10 mL PACgene CCF tubes for plasma + buffy coat.
    - 2 x 8 mL CPT tubes for phenotyping of PBMC.
  - ✓ Tumor biopsy: 2 frozen, 2 FFPE

  If the tumor sample volume is not sufficient to obtain four biopsy cores, priority will be given according to the algorithm detailed in Table 6

Table 6 - Priority order for biopsy cores

Priority order for biopsy cores collection depending on the tumor location/volume	Number of cores collected	Cores' preparation
1 (favorable situation)	4	2 Frozen, 2 FFPE
2	3	2 Frozen, 1 FFPE
3	2	1 Frozen, 1 FFPE
4 (unfavorable situation)	1	1 Frozen

7.2. Visits and assessment during treatment period

During the treatment period, patients will have a trial visit on the first day of each treatment cycle, every 21 days. A general +/-3 day-window is allowed for assessments or visits performed, unless otherwise indicated. In addition, patients will have a scheduled visit on prior to D1 cycle 3 for an optional tumor biopsy. However, depending on treatment tolerance, additional visits and examinations may be scheduled. All examinations revealing a toxicity related to one of the trial treatment must be periodically repeated until toxicity disappearance (or until it is deemed irreversible).

- **Physical examination:** every cycle +/- 3 days
  - ✓ Complete clinical examination (every 2 cycles).
  - ✓ Patient's weight.
  - ✓ ECOG performance status (Appendix 1).
  - ✓ Vital signs (pulse rate, systolic and diastolic blood pressure, and temperature).
  - ✓ Concomitant medications.
  - ✓ Ongoing toxicities or adverse event using the NCI-CTCAE v5.0 (Appendix 8).
- **Paraclinical examination:** every 2 cycles +/- 7 days

- ✓ Radiologic assessment (RECIST v1.1 [Appendix 6] and iRECIST [Appendix 5]) on D1 cycle 3 then on D1 of every 2 cycles (cycle 5, 7, 9…) during the first 12 months of treatment phase, and every 12 weeks thereafter: CT scan.

- **Biological tests:** every cycle +/- 3 days
  - ✓ Hematology: hemoglobin, red blood cell count, WBC, ANC, lymphocyte count, and platelet count.
  - ✓ Blood ionogram: Na, K, Ca, Mg, P, Cl, CO_2, total protein, albumin.
  - ✓ Renal function: blood urea, creatinine
  - ✓ Hepatic function: direct bilirubin, alkaline phosphatase, ALT, AST, GGT.
  - ✓ Fasting plasma glucose, creatine phosphokinase.
  - ✓ Squamous cell carcinoma antigen (SCCa) (every 2 cycles, starting from C3D1).
  - ✓ Pregnancy test (if applicable): serum pregnancy test (starting from C2D1).
- **Additional biological tests:** C1D8, C1D15, C2D8, C2D15,C3D8, C3D15 CxD8*, CxD15*

  **From Cycle 4, additional biological tests at CxD8 CxD15 will be performed in case of grade ≥ 2 abnormalities observed on creatinine level or platelets count during the first 3 cycles.*

- ✓ Platelet count creatinine level, glucose, electrolytes, magnesium and calcium.

- **Other biological tests:** every two cycles, starting from C1D1 +/- 3 days
  - ✓ Thyroid hormone: TSH, free T3, free T4.
- **Translational research**
  - ✓ Mandatory blood sample (on D1 of C3 and D1 of C5).
    - 2 x 10 mL PACgene CCF tubes for plasma + buffy coat.
    - 2 x 8 mL CPT tubes for phenotyping of PBMC.
  - ✓ Optional tumor biopsy (on D1 of C3): when possible, 2 frozen and 2 FFPE.

    If the tumor sample volume is not sufficient to obtain four biopsy cores, priority will be given according to the algorithm detailed in Table 6

7.3. End-of-treatment visit

Patients will perform an end-of-treatment visit 1 week after the discontinuation of all investigational products (pembrolizumab AND vorinostat), either due to disease progression or to other reasons indicated in Section 5.7 .This visit will include the following:

- **Physical examination:**
  - ✓ Patient's weight.
  - ✓ ECOG performance status (Appendix 1).
  - ✓ Vital signs (pulse rate, systolic and diastolic blood pressure, and temperature).
  - ✓ Concomitant medications.

- ✓ Ongoing toxicities or adverse event using the NCI-CTCAE v5.0 (Appendix 8).

- **Paraclinical examination:**
  - ✓ Radiologic assessment (RECIST v1.1 [Appendix 6] and iRECIST [Appendix 7]): CT scan. Tumor imaging should not be repeated if it was obtained less than 4 weeks from withdrawal of therapy.
- **Biological tests:**
  - ✓ Hematology: Hemoglobin, red blood cell count, WBC, ANC, lymphocyte count, and platelet count.
  - ✓ Blood ionogram: Na, K, Ca, Mg, P, Cl, CO_2, total protein, albumin.
  - ✓ Renal function: blood urea, creatinine.
  - ✓ Hepatic function: direct bilirubin, alkaline phosphatase, ALT, AST, GGT.
  - ✓ Thyroid hormone: TSH, free T3, free T4.
  - ✓ Fasting plasma glucose, creatine phosphokinase.
  - ✓ Squamous cell carcinoma antigen (SCCa).
  - ✓ Pregnancy test (if applicable): serum pregnancy test.
- **Translational research:**
  - ✓ Blood sample at progression
    - 2 x 10 mL PACgene CCF tubes for plasma +buffy coat
    - 2 x 8. mL CPT tubes for phenotyping of PBMC
  - ✓ Optional tumor biopsy at progression: when possible, 2 frozen and 2 FFPE.

    If the tumor sample volume is not sufficient to obtain four biopsy cores, priority will be given according to the algorithm detailed in Table 6

7.4. Follow-up

Patients will perform follow-up visits every 3 months after the end-of-treatment visit for 1 year. This visit will include the following:

- ✓ Disease and survival status: information may be collected during visits or telephone calls.
- ✓ Collection of persistent or long term occurring toxicities.

  Ongoing toxicities or AEs must be monitored until resolution or returned to baseline level.
  - All AEs from the time of treatment inclusion through 30 days following cessation of study treatment must be reported by the investigator.
  - All AEs meeting serious criteria, from the time of treatment allocation/randomization through 90 days following cessation of study treatment, or 30 days following cessation of study treatment if the participant initiates new anticancer therapy, whichever is earlier must be reported by the investigator.

- ✓ First new antineoplastic therapy after study treatments termination.
- ✓ Tumor assessment (if applicable) :

  In participants who discontinue study treatment without documented disease progression, every effort should be made to continue monitoring their disease status by tumor imaging using the same imaging schedule used while on treatment (every 6 weeks in year 1 or every 12 weeks after year 1) to monitor disease status until the start of a new anticancer treatment, disease progression, pregnancy, death, withdrawal of consent, or the end of the study, whichever occurs first.

7.5. Provisions in case of treatment or trial interruption

In case of treatment or study discontinuation, the patient will have to be followed as indicated in Sections 3.6, 3.7, and 5.7. However, further treatment will be at the investigator's discretion as per standard of care.

8. REPORTING OF ADVERSE EVENTS

8.1. Adverse event: general definition

An adverse event (AE) is defined as any untoward medical occurrence, in a patient or clinical trial subject treated by a medicinal product and which does not necessarily have a causal relationship with this treatment.

8.2. Serious adverse event: general definition

A serious adverse event (SAE) is defined as any untoward medical occurrence that at any dose:

- Results in death.
- Is life-threatening.
- Requires hospitalization or prolongation of existing hospitalization.
- Results in persistent or significant disability/incapacity.
- Is a congenital anomaly/birth defect.
- Is medically relevant in the context of the pathology and the clinical trial.

These characteristics/consequences are to be considered at the time of the event. For example, regarding a life-threatening event, this refers to an event in which the subject was at risk of death at the time of the event; it does not refer to an event which hypothetically might have caused death if it was more severe.

The terms disability and incapacity correspond to any clinically relevant physical or psychological handicap, transient or permanent, which impact the patient's physical condition/activity and/or the quality of life.

Medical and scientific judgment should be exercised in deciding whether other situations should be considered serious, such as important medical events that might not be immediately life-threatening or result in death or hospitalization, but might jeopardize the patient or might require intervention to prevent one of the other outcomes listed in the definition above (for example: overdose [see section 8.4], second cancer, etc.).

The investigator will assess whether a reasonable causal relationship exists between the event and the treatment/therapy. If the sponsor disagrees with the investigator's causality assessment, the opinion of both the investigator and the sponsor will be reported.

The following events are considered as SAEs but should not be managed according to the section 8.2.7. These events do not require immediate reporting and should be reported only in the case report form:

- Hospitalization occurring in the context of tumor progression of disease under trial,
- Progression of disease under trial,
- Events related to progression of disease under trial.

The following events leading to a hospitalization or a prolongation of hospitalization are not considered as Serious Adverse Events:

- Hospitalization already scheduled before the start of the trial,
- Hospitalization required as part of the protocol (biopsy, chemotherapy, etc.

A Suspected Unexpected Serious Adverse Reaction (SUSAR) is defined as any serious adverse reaction, the nature, severity or outcome is not consistent with the applicable drug information (e.g. IB for an unapproved investigational product or package insert for an approved product).

The safety reference documents for this study to assess whether an AE is expected or unexpected are:

- Vorinostat: most recent Summary of Product Characteristics (SmPC)
- Pembrolizumab: Investigator Brochure (IB)

The assessment of expected/unexpected character of the event is the responsibility of the sponsor.

- **New event:** is defined as any new event relating to the conduct of the trial or the development of the investigational medicinal product where that new event is likely to affect the safety of the subjects.
- **Severity criterion:** the severity criterion must not be confused with the seriousness criterion which is the guide for defining the reporting requirements.

The intensity (severity) of events will be estimated using the extract of NCI-CTCAE v5.0 classification (Appendix 8). The intensity of adverse events not listed in this classification will be assessed according to the following qualifiers:

- **Grade 1 (mild):** asymptomatic or mild symptoms; clinical or diagnostic observations only; intervention not indicated.
- **Grade 2 (moderate):** minimal local or noninvasive intervention indicated; limiting age-appropriate instrumental ADL.
- **Grade 3:** severe or medically significant but not immediately life-threatening; hospitalization or prolongation of hospitalization indicated; disabling; limiting self-care ADL.
- **Grade 4:** Life-threatening consequences; urgent intervention indicated.
- **Grade 5:** Death related to the event.

8.3. Measures to be taken in case of a serious adverse event

The investigator ensures that adequate medical care is provided to the patient.

The investigator must immediately following knowledge of the event, notifies the R&D UNICANCER pharmacovigilance unit of any SAE or any new event defined here above, whether or not related to the research, which occurs during the 'trial reporting period'. This reporting period:

- Starts at the date of the signature of the informed consent form*.
- Covers the entire period during which the patient is receiving the investigational treatment or is subject to specific procedures related to the trial.
- Covers a period of 90 days after the last administration of the investigational product**.

*Between the signature of the informed consent form and the start of the study treatment, only SAE related to the study procedures (biopsy and or blood samples) must be reported.

**Any later SAE, i.e. occurring after a period of 90 days, which is considered to be related to the experimental treatment(s) or to the research (other treatment used, diagnostic procedures and examinations carried out during the research) must be reported without any limitation in terms of deadline.

Notification must be carried out immediately by fax to the R&D UNICANCER pharmacovigilance unit by sending the form "notification of a SAE", located in the Investigator Master File, completed as precisely as possible, dated and signed by the physician-investigator:

R&D UNICANCER

Pharmacovigilance unit, France

Phone: +33 (0)1 44 23 04 16 – Fax: +33 (0)1 44 23 55 70

Email: fax-pv@unicancer.fr

Abnormal laboratory results should be reported as SAE if they possibly put at risk the patient or they require medical intervention to prevent an outcome corresponding to one of severity criteria.

Second cancer, whether or not related to the research, must be reported to the R&D UNICANCER pharmacovigilance unit without any limitation in terms of deadline.

The investigator **shall send additional information to the R&D UNICANCER pharmacovigilance unit** using a SAE declaration form (by ticking the Follow-up X box to specify that it is a follow-up and not an initial report) as soon as he is aware of the event. The investigator must also submit the last follow-up at the resolution or stabilization of the SAE.

The investigator is responsible for appropriate medical follow-up of patients until the resolution or stabilization of the event or until the death of the patient. This can sometimes mean that the follow-up continues after the patient has left the trial.

The investigator must keep the documents concerning the suspected SAE in order to supplement the information previously submitted if necessary.

Requests for clarification and additional information may be sent to the investigator by the R&D UNICANCER pharmacovigilance unit or CRA sponsor of the trial to document and treat the case.

The physician-investigator should also attach to the form «notification of a SAE», whenever possible:

- a copy of the hospital report or extended hospitalization report,
- a copy of all results of additional investigations carried out, including also relevant negative results, and enclosing the normal laboratory values,
- a copy of the autopsy report if necessary,
- any other document deemed to be useful and pertinent.

All these documents must be anonymized.

In the event of pregnancies:

Pregnancy is not considered as an SAE but must be reported with the same modalities as an SAE, using a Pregnancy Notification Form. Pregnancy will be subject to a specific follow-up until the end of pregnancy. While pregnancy is not considered as a SAE, any anomaly detected in the fetus or child, any elective termination of a pregnancy for medical reasons, or spontaneous abortion will be reported as an SAE, using the same procedure as an SAE.

Any pregnancy in a female partner of a male subject occurring during the treatment period or within f4 months following the last dose of pembrolizumab or 6 months following the last dose of vorinostat, must be reported using the same procedure as an SAE.

Overdose

Definition: Administration of a quantity of a medicinal product given per administration or cumulatively which is above the maximum recommended dose according to the authorised product information.

Vorinostat: All overdose of Vorinostat must be reported to the sponsor. The notification will be done by using the SAE notification form according to the section 8.3 of this protocol.

Pembrolizumab: see section 8.4

8.4. Events of Clinical Interest (ECIs)

ECIs must be reported to the sponsor immediately as per the SAE notification instructions described in Section 8.3, even if not fulfilling a seriousness criterion.

Events of clinical interest for this study include:

- An overdose of pembrolizumab that is not associated with clinical symptoms or abnormal laboratory results. For purposes of this study, an overdose of pembrolizumab will be defined as any dose of 1,000 mg or greater (≥5 times the indicated dose). No specific information is available on the treatment of overdose of pembrolizumab. In the event of overdose, the participant should be observed closely for signs of toxicity. Appropriate supportive treatment should be provided if clinically indicated. If an adverse event(s) is associated with ("results from") the overdose, the adverse event(s) must be reported as a serious adverse event, even if no other seriousness criteria are met.
- An elevated AST or ALT lab value that is greater than or equal to 3X the upper limit of normal and an elevated total bilirubin lab value that is greater than or equal to 2X the upper limit of normal and, at the same time, an alkaline phosphatase lab value that is less than 2X the upper limit of normal, as determined by way of protocol-specified laboratory testing or unscheduled laboratory testing.*

  Note: These criteria are based upon available regulatory guidance documents. The purpose of the criteria is to specify a threshold of abnormal hepatic tests that may require an additional evaluation for an underlying etiology.

9. TRANSLATIONAL STUDIES

All patients enrolled in this study will participate in the translational study. Collection of all blood and baseline tumor samples are mandatory. Collection of on-treatment or at progression tumor samples are optional and patient must signed a separate ICF. The collection and use of the patient's biological samples will not modify or influence the diagnosis, medical care, and treatment administered to the patient.

The biological samples expected in this research are described in section 13.7.2.

All the samples will be transported and centralized at Integrated BioBank of Luxembourg (IBBL), under the responsibility of Dr Kristin Kornerup, located at the following address:

IBBL, INTEGRATED BIOBANK OF LUXEMBOURG

1, rue Louis Rech, L-3555 Dudelange

Luxembourg

The tissue and blood samples collected will be used to identify biomarkers that may be predictive of response or toxicity to the proposed treatments and/or prognostic for squamous cell carcinoma of the head and neck, cervix, lung, anal, vulva/vagina, and penile. The collected tumor tissue and blood samples may also be used to develop and validate diagnostic assays and allow the generation of statistically meaningful biomarker data. A detailed description of the translational studies is provided in Appendix 12.

10. DESCRIPTION OF STATISTICAL METHODS

10.1. Statistical hypothesis and sample size determination

The primary aim of the study is to assess the antitumor activity of the combination of pembrolizumab and vorinostat in patients with selected recurrent and/or metastatic solid tumors.

ORR reported in the literature with pembrolizumab or nivolumab in phase III clinical trials ranged from 6% to 24% depending on the tumor type.

The primary endpoint is the rate of patient presenting an objective response (defined as the proportion of patients with best response of a complete response (CR) or a partial response (PR) according to RECIST v1.1.) while on study protocol.

The required number of evaluable patients for each cohort was determined using an A'Hern design (A'Hern, 2001) based on different hypotheses. Design parameters and decision rules for each cohort are summarized in the following table:

Cohort	p0	p1	Alpha	Power	Nb of evaluable / included patients	Sufficiently active
Head and Neck	10%	35%	5%	85%	19 / 21	≥5 CR/PR
Cervix	5%	30%	5%	90%	16 / 18	≥3 CR/PR
Lung	15%	40%	5%	85%	23 / 25	≥7 CR/PR
Anus	15%	40%	5%	85%	23 / 25	≥7 CR/PR
Penis	5%	30%	10%	85%	10 /11	≥2 CR/PR
Vulva/vagina	5%	30%	10%	85%	10 / 11	≥2 CR/PR

- p0: maximal unacceptable rate of patient presenting an objective response for whom the experimental treatment will be considered as insufficiently active.
- p1: minimal acceptable rate of patient presenting an objective response for whom the experimental treatment will be considered as sufficiently active.

To compensate for drop out, we plan to include an additional 10% of patients in each cohort; therefore a total of 111 patients are required this study.

10.2. Trial populations to be analyzed

The following populations will be considered for analyses:

- Per-protocol: all eligible patients with at least one valid post-baseline disease assessment (or with disease progression) and have received at least one dose of the study treatment (pembrolizumab or vorinostat).
- Safety population: all patients who have started their allocated treatment (at least one dose of the study treatments).

10.3. Planned statistical analysis

Demographic and clinical characteristics (including HPV status) will be presented in the overall population and per cohort using usual statistics. Quantitative data will be summarized as median, min, max and number of missing data. Qualitative variables will be described as number, percentage and number of missing data.

Primary endpoint

The primary endpoint will be assessed in the per protocol population and will be reported per cohort. The primary endpoint is the rate of patients presenting an objective response according to RECIST v1.1 It will be presented as number, percentage, and 95% confidence interval (CI): by the binomial exact distribution.

Secondary efficacy endpoints

The secondary endpoint will be assessed in the per protocol population and will be reported per cohort:

- ORR, iORR, and DOR will be presented using frequency, percentage frequencies, percentages, and the 95% CI (Binomial exact distribution).
- A radiological panel will centrally assess the ORR, iORR, and iPFS.
- Survival rates (PFS, iPFS OS) will be estimated at different time points using the Kaplan-Meier method. Median survival times will be estimated and reported with the corresponding 95% CI.
- Number of pembrolizumab injections and dose intensity of vorinostat will also be recorded.
- Incidence rates of adverse events and serious adverse events will be presented using frequencies and percentages by system organ class and MedDRA preferred term. Pharmacovigilance will be handled by UNICANCER.
- Safety Analysis will be performed on the safety population, by cohort and on the overall population. Each of the following will be assessed:
    - AEs by toxicity grade
    - SAEs
    - Investigated drug-related AE
    - AE leading to interruption or dose reduction
    - Occurrence of AE with toxicity grade >2

  Frequency and percentage will be computed for each event

  Pharmacovigilance will be handled by UNICANCER.

10.3.1. *Modifications of SAP and initial statistical analysis strategy*

Any modification/change made to the initial statistical analysis plan (SAP) will be described in details, well-argued and commented in an updated version of the SAP. These modifications may be complementary/exploratory analyses not envisaged initially.

11. OVERSIGHT COMMITTEES

11.1. Independent data monitoring committee

An IDMC, with expertise and experience in the pathologies, and without direct involvement in the conduct of the trial, will be set up specifically to guarantee:

- Effective protection of patients.
- Insure the ethical conduct of the trial.
- Benefit/risk ratio of the trial.
- Ensure the independent review of the scientific results during the trial and at the end of the trial.

The IDMC will meet every year and will be composed of at least:

- 2 oncologists
- A statistician

Data presented to IDMC are strictly confidential. Interim analyses, if applicable, will be presented to the IDMC.

The IDMC may recommend the early termination of the trial if one of the following conditions is met:

- An unacceptable toxicity.
- Data available from the trial or any other source of information are sufficiently convincing to influence the therapeutic practice of the majority of clinicians.

The IDMC has only a consultative role; it will inform the sponsor who will decide whether the IDMC recommendation will be followed.

11.2. Steering Executive Committee

A Steering executive committee has been implemented for this trial. The Steering executive committee will meet physically (or at least through a teleconference) in a regular basis (at least twice a year) to discuss key questions related to the protocol (see examples listed below). Additional meetings may be organized, as required.

The Steering executive committee is composed of (non-exhaustive list):

- The principal investigator
- UNICANCER project manager
- The ancillary/translational program coordinator
- The study statistician
- The referent pathologist
- Some investigators involved in the study

The steering committee will be responsible for:

- Approving the protocol and any amendments thereof.

- Maintaining the scientific integrity of the study, according to the protocol.
    - Ensuring the trial is conducted in an ethical manner; reviewing recommendations of the IDMC and determining actions to be taken as applicable in order to ensure the safety of the participants.
    - Reviewing the recruitment rate and any kind of issues raised during the conduct of the study to adjust the protocol conditions via a protocol amendment, if needed.
    - Reviewing and deciding on research projects requiring access to and use of study data and/or biological samples.
    - Publications and presentations of the study data.

The steering executive committee will assist UNICANCER in resolving issues and/or questions encountered during the trial and will consider with UNICANCER changes to the protocol as necessary.

12. QUALITY ASSURANCE

12.1. Data collection

All data necessary for the research must be entered into the trial eCRFs in a timely manner. eCRFs will be completed by the principal investigator and other staff members duly designated. The data entered must be accurate and complete.

The trial database will be hosted by:

Institut du Cancer Montpellier (ICM) – Val d'Aurelle

Unité de Biométrie – CTD INCa

208 rue des Apothicaires - Parc Euromédecine

34298 Montpellier Cedex 5 – France

Mrs. Sophie Gourgou will be responsible for the trial database.

Database management will be provided by an eCRF developed using the CSOnline module of Ennov Clinical® software. In case of technical problem with the eCRF, the investigator may refer to the specific operating procedure of the eCRF or directly contact:

ICM – Unité de Biométrie – CTD INCa

Data centre UNICANCER

from Monday to Friday 9 am-5 pm

Email: support.ecrf@icm.unicancer.fr

Fax: +33 (0)4 67 61 37 18

Tel: +33 (0)4 67 61 45 48/24 52

The access code (login) and passwords to access the eCRF will be sent directly to each users personal email account. The logins and personal passwords to connect to the eCRF, via the website - https://ecrf.icm.unicancer.fr/CSOnline - will automatically be generated by CSOnline.

A password non-disclosure certificate will be signed by the principal investigator of each center engaging his/her responsibility regarding the confidentiality of the access codes for all users of the eCRF at their center.

Trial data will be entered directly by the principal investigator or by designated staff members of each center, via the eCRF, and will be controlled and validated according to the standard procedures (included those in the software and the sponsor's quality assurance procedures). When using the eCRF, traceability of access and changes made to the eCRF are traced by the software (audit trail). At the end of the trial and once all the eCRF data are validated, the investigator will login to the eCRF to sign all the pages to validate the data entered for each patient.

The sponsor will create and send an electronic copy (PDF file) of each patient's CRF to the corresponding investigator. This pdf file must be printed and signed by the investigator, and then archived at the investigator's site.

12.2. Access to data

The sponsor has direct access to all investigator sites, original records, source data/document and reports to allow quality control and auditing by the sponsor or on behalf of the sponsor.

Investigators will make available to the authorized persons the documents and the patients' individual data that are essential to monitor the trial on an ongoing basis, to perform quality control and audit of this research in accordance with national regulatory requirements.

12.3. Trial monitoring

To ensure the patient integrity and safety, and authenticity and credibility of data in accordance with the "Décision portant sur les Bonnes Pratiques Cliniques, 24 November 2006", the sponsor had established a quality assurance system that consists of:

- The management and the monitoring of the trial according to UNICANCER procedures. The monitoring strategy is built according to a systematic, prioritized, risk-based approach, and is documented in the monitoring plan.
- The quality control of data at the investigational centers by the monitor(s), which involves:
  - Verifying that the protocol, as well as the current guidelines ICH-GCP, the national regulatory requirements, are adhered to.
  - Verifying the informed consent and the eligibility of each patient participating in the trial.
  - Verifying that the CRF data is consistent and in agreement with the source documents.
  - Verifying the notification of each SAE.
  - Verifying the drug traceability (dispatching, storage, and accountability).
  - Verifying that patients are not already participating in another clinical study making them ineligible for this protocol. The monitor will also verify that patients have not participated in another study within the delay indicated in the non-inclusion criterion N°18.
- The quality control of data by a centralized monitoring process. Centralized monitoring is a remote evaluation of data, performed in a timely manner, supported by appropriately qualified and trained persons (e.g., data managers, biostatisticians). Review, that may include statistical analyses, of accumulating data from centralized monitoring can be used to:
  - Identify missing data, inconsistent data, data outliers, unexpected lack of variability, and protocol deviations.
  - Examine data trends such as the range, consistency, and variability of data within and across sites.
  - Evaluate for systematic or significant errors in data collection and reporting at a site or across sites; or potential data manipulation or data integrity problems.
  - Analyze site characteristics and performance metrics.

- Select sites and/or processes for targeted on-site monitoring.

- The audit of participating investigational centers when deemed necessary

The monitors/CRAs in charge of trial monitoring will be mandated by the sponsor. They must have direct access to all patient data required to perform their duty in accordance with the national regulatory requirements. The monitors/CRAs are bound by professional secrecy under the national regulatory requirements. Written reports must be issued to ensure the traceability of monitoring visits.

To ensure optimal research quality control the investigator will ensure that the monitor/CRA has direct access to all trial patient files.

12.4. Audits and inspections

As part of UNICANCER's audit program, the sponsor may audit some investigational centers. The center and the investigator agree that audits be carried out by Sponsor or any person duly authorized during the trial and for at least 15 years after the trial.

The investigational center and the investigator agree to devote the time necessary for the audit procedures, allow the control of the trial documentation, and provide additional information requested by the sponsor.

A Competent Authority may also request a trial inspection (during the trial or after its completion). If a Competent Authority requests an inspection, the investigator must inform the sponsor immediately of this request. The investigator must allow the inspectors direct access to the trial documents and source documents.

The investigational center and the investigator agrees to devote the time necessary for inspections procedures, allow the control of the trial documentation, and provide additional information requested by the inspectors of the concerned Competent Authority.

13. ETHICAL AND REGULATORY CONSIDERATIONS

13.1. General requirements

The trial must be conducted in accordance with the French national regulatory requirements:

- The principles of ethics as stated in the last version of the Declaration of Helsinki.
- Loi n°2012-300 du 5 mars 2012 relative aux recherches impliquant la personne humaine, as modified in 2016.
- Regulation (EU) 2016/679 on the protection of natural persons with regard to the processing of personal data and on the free movement of such data (General Data Protection Regulation).
- Amended *Loi Informatique et Libertés n° 78-17 du 6 janvier 1978, relative à la protection des personnes physiques à l'égard des traitements de données à caractère personnel*.
- Amended *Loi n° 2004-800 du 6 août 2004, relative à la bioéthique*.
- *Décision du 24 novembre 2006 fixant les règles de bonnes pratiques cliniques pour les recherches biomédicales portant sur des médicaments à usage humain.*
- Good Manufacturing Practices, in particular, Annex 13 on investigational medicinal products.

13.2. Patient identification

All patients will receive a unique patient identification number when signing the informed consent form by the patient and before any trial procedure is performed. This number will be used to identify the patient throughout the trial and must be used on all trial documentation related to this patient. The patient identification number must remain constant throughout the trial.

13.3. Patient information and consent

Patient information and informed consent from the patient must be handled in accordance with the “French regulation, especially article L.1122-1 and subsequent articles.

Prior to the participation of a patient in the trial, this patient will be informed both verbally and in writing about the objectives of the trial, its methods, anticipated benefits and potential risks and the discomfort to which they may be exposed. All items must be explained by the investigator in a language and in terms that are easy to understand by the patient. The patients must be given enough time to consider their participation and decide whether they wish to participate or not in the trial. Patients will also be informed that their participation is voluntary and that they have the right to withdraw from the trial at any time without giving the reasons and without this impacting their subsequent medical care.

The patient information sheet and the informed consent form must be associated within the same document to ensure that all information regarding the trial is provided to the patient. Patients will confirm their consent in writing prior to starting the trial and before undergoing any trial-related procedure. Two original informed consent forms must be personally dated and signed by the patient and investigator. An original copy will be filed in the Trial Master File (TMF). The other original patient information sheet and the signed informed consent form will be given to the patient.

If the patient decides to withdraw from the trial, the patient is not obliged to give reason(s) for withdrawing. However, the investigator should make a reasonable effort to obtain the reason(s) while fully respecting the patient's rights.

In conformance with the data protection regulation, the patient may use their right to access to, rectify or oppose the use of their personal data in the research. In these situations, the investigator shall inform the sponsor without delay in order to take the appropriate steps.

If any changes in the written patient information or informed consent form occur during the trial, the investigator will ensures that all patients impacted by the changes and still participating in the trial receive the updated patient information in a timely manner and are asked for written consent for the changes made.

13.4. Insurance compensation

UNICANCER, the sponsor of the trial certifies that it has taken out a civil liability insurance policy covering its civil liability for this clinical trial under its sponsorship. This insurance policy is in accordance with local laws and requirements. The insurance of the sponsor does not exempt the investigator and its team from maintaining their own liability insurance policy.

13.5. Investigator responsibilities

The principal investigator of each investigator site participating in the trial commits to conduct the trial as specified in this protocol and in accordance with the "Décision portant sur les Bonnes Pratiques Cliniques, 24 November 2006".

- It is the responsibility of the principal investigator to:
  - Provide to the sponsor with their curriculum vitae (CV) and those of their collaborators, and evidence that the center will be able to conduct the trial. The CV must be current (no older than 1 year), dated and signed.
  - Identify the members of their team who participate in the trial and define each team member's role and responsibilities.
  - Start recruiting patients only after receiving approval from the sponsor.
  - Be available for monitoring visits, audits, and investigator meetings.
- It is the responsibility of each principal investigator and each investigator team member to:
  - Ensure the confidentiality of all data recorded during the trial.
  - Collect the informed consent, written, dated, and signed personally by each individual research participant before any specific selection procedure for the trial.
  - Regularly complete the eCRF for each patient included in the trial and allow CRA(s), mandated by the sponsor, direct access to the source documents in order to validate the data collected in the CRF.
  - Declare to the sponsor as soon as being aware of, any serious adverse event occurring during the trial according to provisions of this protocol;

- Accept regular visits by the CRA(s) and possibly those of auditors mandated by the sponsor or the inspectors of the respective regulatory authorities;
- Date, correct, and sign the corrections made in the CRF and the requests of the data correction forms (DCF) for each patient included in the trial.

13.6. Federation of the Patient Committees for Clinical Research in Cancerology

This committee reviews trial documents provided to patients in oncology clinical studies, and makes suggestions for improving these, in terms of the quality of information given to patients.

The "Ligue Nationale Contre le Cancer" and the French NCI (INCa) coordinate the French patient committees' federation.

13.7. Human biological samples collection

Biological studies are necessary to increase the knowledge of diseases, which may allow the development of new and more effective treatments. These studies use human biological samples (blood and tumor samples) than are collected from patients either while they receive medical care (examination, surgery) or specifically for the research purpose.

These biological samples will be prepared, stored, shipped, and used for the purpose of research.

These biological samples are subject to written consent from the patient. This consent is revocable at any time during the trial. Similarly, at any time during the research, the patient has the possibility to request the destruction of their samples.

Furthermore, it must be noted that the results of biological studies may be published only if all data relative to the patients are made anonymous.

Concerning **genetic research**, patients must consent to participation in these studies after being informed of the proposed research, irrespective of the type of sample collected (already existing or specifically collected).

Furthermore, it must be noted that the results of biological studies may be published only if all data relative to the patients are made anonymous.

Refer to the Appendix 12 for more detailed information.

13.7.1. *Storage and use of disease assessment samples (blood, biopsy, tumor specimen, etc.)*

During the medical cares that are going to be realized, biological samples of tissues and/or cells (blood, tumor tissue) will be collected for medical purposes. A part of these samples may be stored and used for scientific researches.

These samples will be used to investigate potential biomarkers predictive of response to pembrolizumab, vorinostat, or their combination in the PEVOsq study.

The patient will be informed via a patient information sheet and, in the absence of opposition of his part, biological samples for research will be prepared, stored and used for this research.

The preparation, storage and use of the biological samples does not modify or imply any change with respect to the diagnosis, cares and treatments that will be administrated to the patient.

Refer to the Appendix 12 for more detailed information.

13.7.2. *Collecting additional biological samples for research purpose*

Tumor and blood sampling will be performed at several time points during the course of the treatment in order to collect molecular and immune parameters for integrative analyses with the aim to better understand the mechanisms of response or resistance to this combination. This research also aim to determine how integrate these data to determine the best treatment option appropriate to specific patients.

To perform this research, additional blood and tumor samples will be collected:

- Mandatory tumor samples to be obtained for all the patients
  - ➢ Tumor biopsy at baseline (2 frozen + 2 FFPE)
- Optional tumor samples to be obtained for willing patients
  - ➢ Tumor biopsy at D1 of cycle 3 (2 frozen + 2 FFPE)
  - ➢ Tumor biopsy at disease progression (2 frozen + 2 FFPE)
- Mandatory blood samples
  - ➢ Blood for ctDNA analysis at baseline:
    - o 2 x 10 mL PACgene CCF tubes for plasma +buffy coat.
    - o 2 x 8. mL CPT tubes for phenotyping of PBMC.

  Then at D1 cycle 3, at D1 cycle 5 and if applicable, at progression:
    - o 2 x 10 mL PACgene CCF tubes for plasma +buffy coat.
    - o 2 x 8. mL CPT tubes for phenotyping of PBMC.

These biological samples should be prepared, stored and used for the purpose of the research, as described in the Manual Lab provided by the Sponsor.

These additional samples are subject to a specific additional written consent from the patient. This specific consent for the ancillary/translational research is revocable at any time. In addition, the patient has the right to request the destruction of their samples at any moment.

14. DATA PROCESSING AND CONSERVATION OF DOCUMENTS AND DATA OF THE RESEARCH

14.1. Data processing

14.1.1. *Under the responsibility of the sponsor*

The statistical data will be transferred to the trial statistician for analysis. The trial data remain the property of UNICANCER, the research sponsor.

The software Clinsight® will be used for data entry, management, and archiving of data. The statistical analysis will be performed using the SAS and/or stata softwares software.

14.1.2. *In the investigational center, when computerized medical records are used*

If computerized patient records are used in a participating center to process or store trial data, the center must:

- Verify and document that the computer system used to process the data conforms to the requirements concerning data completeness, accuracy, and reliability with respect to expected performances (quality validation).
- Define and follow the standardized procedures related to these systems.
- Ensure that these systems allow modifications of collected data, that each modification is automatically authenticated, and that the data cannot be removed (i.e. any change or modification of the data must be traceable).
- Set up and maintain a security control to prevent unauthorized access to the data.
- Establish and regularly update the list of persons authorized to have access and modify the data.
- Carry out appropriate backups of the data.
- Ensure confidentiality, whenever it is applicable (e.g. during data input).
- Ensure that the individual computerized patient data are processed in accordance with the "Loi Informatique et Libertés n° 78-17, 6 January 1978 modified".

If data are transformed while being processed, it should always be possible to compare them with the original observations/records.

The computerized system used to identify trial patients must not be ambiguous must allow the identification of all data collected for each patient while maintaining confidentiality in accordance with the "Loi Informatique et Libertés n° 78-17, 6 January 1978 modified".

14.2. Retention of documents by investigator sites

The investigator must maintain source documents for each trial patient.

All information in case report forms must be traceable and consistent with source documents, which are generally maintained in the patient's file. The source documents should contain all demographic and medical information, laboratory data, radiology, electrocardiograms, etc., including the original copy of the signed patient information sheet and informed consent form.

The investigator must retain essential documents as described below. The investigator agrees to adhere to the document retention procedures by signing the protocol. Essential documents include:

- Approvals from the CPP for the trial protocol and all relevant amendments.
- Authorizations from the ANSM for the trial protocol and all relevant amendments.
- All source documents and laboratory records.
- CRF copies.
- Patients' informed consent forms.
- Investigator master file (IMF) and Investigator master file-pharmacy (IMF-P).
- Any other pertinent trial document.

All trial documents must be kept in a locked and secured place and be considered as confidential.

Data will be archived under the responsibility of the principal investigator of each participating center according to the "Décision portant sur les Bonnes Pratiques Cliniques, 24 November 2006". The trial documents, including a list of patient's identifications for a minimum period of 25 years after the end of the trial. UNICANCER will inform the investigational centers when the trial-related records are no longer required.

The investigational center may destroy the data only after written authorization from the sponsor.

15. DATA OWNERSHIP AND CONFIDENTIALITY

By signing the protocol, the investigator agrees to keep all information provided by UNICANCER strictly confidential and to ensure similar confidentiality from their staff. This obligation does not cover information provided to the patients and information already publically available.

Trial documents provided by UNICANCER (protocols, investigators' brochures, CRFs, and other material) will be stored appropriately to ensure their confidentiality. The information provided by UNICANCER to the physician-investigator may not be disclosed to others without direct written authorization from UNICANCER.

The physician-investigator commits to not publish, spread or use in any manner, directly or indirectly, the scientific and technical information and results related to the trial.

16. PUBLICATION RULES

All information resulting from this trial is considered to be confidential, at least until appropriate analysis and checking has been completed by the sponsor, the principal investigator and the statistician of the trial.

Any publication, abstract or oral presentations including results of the trial must be submitted to the sponsor (UNICANCER) for approval.

Additionally, all communications, manuscripts or oral presentations must include a section mentioning UNICANCER as well as any institution, physician-investigators, collaborative research group, scientific society that has contributed to the trial, including organizations that have provided financial support.

The first author and writer of the main publication will be the principal investigator. The principal investigator may however designate another person to (co-) write the publication.

As for the main publication, authors are listed in the following order:

- The trial coordinator (first or last author).
- The other investigators will appear in the list of co-authors in decreasing order, according to the number of recruited patients regardless of their affiliation to a cooperative group.
- A person representing each cooperating group, if a representative is not listed in the sites with the highest recruitment rates.
- The statistician (the statistician's position is among the first three authors or the last author of the publication).
- The ancillary/translational coordinator
- A R&D UNICANCER representative.

Similarly, publication of the sub-studies (e.g. biological/ancillary studies) will include persons who have carried out the sub-studies as well as the names of all individuals who have contributed to these sub-studies and a sponsor representative.

It is desirable to include the contributors from weakly recruiting centers who have not been mentioned in the first article in the later publications.

Any conflict regarding publication authorship will initially be submitted to the trial IDMC and then to the CSR (Comité Stratégique Recherche [Strategic Research Committee]) for resolution in case of major disagreement.

UNICANCER will arbitrate and rule any dispute that may arise.

17. REFERENCES

A'Hern, R. P. (2001). "Sample size tables for exact single-stage phase II designs." Stat Med **20**(6): 859-866.

Bauml, J., T. Y. Seiwert, D. G. Pfister, F. Worden, S. V. Liu, J. Gilbert, N. F. Saba, J. Weiss, L. Wirth, A. Sukari, H. Kang, M. K. Gibson, E. Massarelli, S. Powell, A. Meister, X. Shu, J. D. Cheng and R. Haddad (2017). "Pembrolizumab for Platinum- and Cetuximab-Refractory Head and Neck Cancer: Results From a Single-Arm, Phase II Study." J Clin Oncol **35**(14): 1542-1549.

Brahmer, J., K. L. Reckamp, P. Baas, L. Crino, W. E. Eberhardt, E. Poddubskaya, S. Antonia, A. Pluzanski, E. E. Vokes, E. Holgado, D. Waterhouse, N. Ready, J. Gainor, O. Aren Frontera, L. Havel, M. Steins, M. C. Garassino, J. G. Aerts, M. Domine, L. Paz-Ares, M. Reck, C. Baudelet, C. T. Harbison, B. Lestini and D. R. Spigel (2015). "Nivolumab versus Docetaxel in Advanced Squamous-Cell Non-Small-Cell Lung Cancer." N Engl J Med **373**(2): 123-135.

Bray, F., J. Ferlay, I. Soerjomataram, R. L. Siegel, L. A. Torre and A. Jemal (2018). "Global cancer statistics 2018: GLOBOCAN estimates of incidence and mortality worldwide for 36 cancers in 185 countries." CA Cancer J Clin **68**(6): 394-424.

Brianti, P., E. De Flammineis and S. R. Mercuri (2017). "Review of HPV-related diseases and cancers." New Microbiol **40**(2): 80-85.

Brotherton, J. M., A. C. Budd and M. Saville (2020). "Understanding the proportion of cervical cancers attributable to HPV." Med J Aust.

Bubna, A. K. (2015). "Vorinostat-An Overview." Indian J Dermatol **60**(4): 419.

Chow, L. Q. M., R. Haddad, S. Gupta, A. Mahipal, R. Mehra, M. Tahara, R. Berger, J. P. Eder, B. Burtness, S. H. Lee, B. Keam, H. Kang, K. Muro, J. Weiss, R. Geva, C. C. Lin, H. C. Chung, A. Meister, M. Dolled-Filhart, K. Pathiraja, J. D. Cheng and T. Y. Seiwert (2016). "Antitumor Activity of Pembrolizumab in Biomarker-Unselected Patients With Recurrent and/or Metastatic Head and Neck Squamous Cell Carcinoma: Results From the Phase Ib KEYNOTE-012 Expansion Cohort." J Clin Oncol **34**(32): 3838-3845.

Cohen, E. E. W., D. Soulieres, C. Le Tourneau, J. Dinis, L. Licitra, M. J. Ahn, A. Soria, J. P. Machiels, N. Mach, R. Mehra, B. Burtness, P. Zhang, J. Cheng, R. F. Swaby, K. J. Harrington and K.-. investigators (2019). "Pembrolizumab versus methotrexate, docetaxel, or cetuximab for recurrent or metastatic head-and-neck squamous cell carcinoma (KEYNOTE-040): a randomised, open-label, phase 3 study." Lancet **393**(10167): 156-167.

Cooper, J. S., T. F. Pajak, A. A. Forastiere, J. Jacobs, B. H. Campbell, S. B. Saxman, J. A. Kish, H. E. Kim, A. J. Cmelak, M. Rotman, M. Machtay, J. F. Ensley, K. S. Chao, C. J. Schultz, N. Lee, K. K. Fu and I. Radiation Therapy Oncology Group (2004). "Postoperative concurrent radiotherapy and chemotherapy for high-risk squamous-cell carcinoma of the head and neck." N Engl J Med **350**(19): 1937-1944.

Derman, B. A., K. F. Mileham, P. D. Bonomi, M. Batus and M. J. Fidler (2015). "Treatment of advanced squamous cell carcinoma of the lung: a review." Transl Lung Cancer Res **4**(5): 524-532.

Dotto, G. P. and A. K. Rustgi (2016). "Squamous Cell Cancers: A Unified Perspective on Biology and Genetics." Cancer Cell **29**(5): 622-637.

du Rusquec, P., O. de Calbiac, M. Robert, M. Campone and J. S. Frenel (2019). "Clinical utility of pembrolizumab in the management of advanced solid tumors: an evidence-based review on the emerging new data." Cancer Manag Res **11**: 4297-4312.

Duvic, M. and J. Vu (2007). "Vorinostat: a new oral histone deacetylase inhibitor approved for cutaneous T-cell lymphoma." Expert Opin Investig Drugs **16**(7): 1111-1120.

Eng, C., C. Messick and R. Glynne-Jones (2019). "The Management and Prevention of Anal Squamous Cell Carcinoma." Am Soc Clin Oncol Educ Book **39**: 216-225.

Ferris, R. L., G. Blumenschein, Jr., J. Fayette, J. Guigay, A. D. Colevas, L. Licitra, K. Harrington, S. Kasper, E. E. Vokes, C. Even, F. Worden, N. F. Saba, L. C. Iglesias Docampo, R. Haddad, T. Rordorf, N. Kiyota, M. Tahara, M. Monga, M. Lynch, W. J. Geese, J. Kopit, J. W. Shaw and M. L. Gillison (2016). "Nivolumab for Recurrent Squamous-Cell Carcinoma of the Head and Neck." N Engl J Med **375**(19): 1856-1867.

Frenel, J. S., C. Le Tourneau, B. O'Neil, P. A. Ott, S. A. Piha-Paul, C. Gomez-Roca, E. M. J. van Brummelen, H. S. Rugo, S. Thomas, S. Saraf, R. Rangwala and A. Varga (2017). "Safety and Efficacy of Pembrolizumab in Advanced, Programmed Death Ligand 1-Positive Cervical Cancer: Results From the Phase Ib KEYNOTE-028 Trial." J Clin Oncol **35**(36): 4035-4041.

Freshwater, T., A. Kondic, M. Ahamadi, C. H. Li, R. de Greef, D. de Alwis and J. A. Stone (2017). "Evaluation of dosing strategy for pembrolizumab for oncology indications." J Immunother Cancer **5**: 43.

Gadducci, A., R. Tana, C. Barsotti, M. E. Guerrieri and A. R. Genazzani (2012). "Clinico-pathological and biological prognostic variables in squamous cell carcinoma of the vulva." Crit Rev Oncol Hematol **83**(1): 71-83.

Glynne-Jones, R., P. J. Nilsson, C. Aschele, V. Goh, D. Peiffert, A. Cervantes, D. Arnold, O. European Society for Medical, O. European Society of Surgical, R. European Society of and Oncology (2014). "Anal cancer: ESMO-ESSO-ESTRO clinical practice guidelines for diagnosis, treatment and follow-up." Eur J Surg Oncol **40**(10): 1165-1176.

Gray, J. E., A. Saltos, T. Tanvetyanon, E. B. Haura, B. Creelan, S. J. Antonia, M. Shafique, H. Zheng, W. Dai, J. J. Saller, Z. Chen, N. Tchekmedyian, K. Goas, R. Thapa, T. A. Boyle, D. T. Chen and A. A. Beg (2019). "Phase I/Ib Study of Pembrolizumab Plus Vorinostat in Advanced/Metastatic Non-Small Cell Lung Cancer." Clin Cancer Res **25**(22): 6623-6632.

Heist, R. S., M. Mino-Kenudson, L. V. Sequist, S. Tammireddy, L. Morrissey, D. C. Christiani, J. A. Engelman and A. J. Iafrate (2012). "FGFR1 amplification in squamous cell carcinoma of the lung." J Thorac Oncol **7**(12): 1775-1780.

Jespersen, H., R. Olofsson Bagge, G. Ullenhag, A. Carneiro, H. Helgadottir, I. Ljuslinder, M. Levin, C. All-Eriksson, B. Andersson, U. Stierner, L. M. Nilsson, J. A. Nilsson and L. Ny (2019). "Concomitant use of pembrolizumab and entinostat in adult patients with metastatic uveal melanoma (PEMDAC study): protocol for a multicenter phase II open label study." BMC Cancer **19**(1): 415.

Laengle, J., J. Kabiljo, L. Hunter, J. Homola, S. Prodinger, G. Egger and M. Bergmann (2020). "Histone deacetylase inhibitors valproic acid and vorinostat enhance trastuzumab-mediated antibody-dependent cell-mediated phagocytosis." J Immunother Cancer **8**(1).

Li, C., W. Liu and Y. Cheng (2016). "Prognostic significance of metastatic lymph node ratio in squamous cell carcinoma of the cervix." Onco Targets Ther **9**: 3791-3797.

Llopiz, D., M. Ruiz, L. Villanueva, T. Iglesias, L. Silva, J. Egea, J. J. Lasarte, P. Pivette, V. Trochon-Joseph, B. Vasseur, G. Dixon, B. Sangro and P. Sarobe (2019). "Enhanced anti-tumor efficacy of checkpoint inhibitors in combination with the histone deacetylase inhibitor Belinostat in a murine hepatocellular carcinoma model." Cancer Immunol Immunother **68**(3): 379-393.

Mangano, A., A. Mangano, G. D. Lianos, D. H. Roukos, L. Boni and G. Dionigi (2015). "Head and neck squamous cell carcinoma and human papillomavirus: epidemiology, treatment and future trends." Future Oncol **11**(6): 889-891.

Marur, S. and A. A. Forastiere (2016). "Head and Neck Squamous Cell Carcinoma: Update on Epidemiology, Diagnosis, and Treatment." Mayo Clin Proc **91**(3): 386-396.

Mazzone, R., C. Zwergel, A. Mai and S. Valente (2017). "Epi-drugs in combination with immunotherapy: a new avenue to improve anticancer efficacy." Clin Epigenetics **9**: 59.

Morris, V. K., M. E. Salem, H. Nimeiri, S. Iqbal, P. Singh, K. Ciombor, B. Polite, D. Deming, E. Chan, J. L. Wade, L. Xiao, T. Bekaii-Saab, L. Vence, J. Blando, A. Mahvash, W. C. Foo, C. Ohaji, M. Pasia, G. Bland, A. Ohinata, J. Rogers, A. Mehdizadeh, K. Banks, R. Lanman, R. A. Wolff, H. Streicher, J. Allison, P. Sharma and C. Eng (2017). "Nivolumab for previously treated unresectable metastatic anal cancer (NCI9673): a multicentre, single-arm, phase 2 study." Lancet Oncol **18**(4): 446-453.

Morton, M., N. Melnitchouk and R. Bleday (2018). "Squamous cell carcinoma of the anal canal." Curr Probl Cancer **42**(5): 486-492.

Naumann, R. W., A. Hollebecque, T. Meyer, M. J. Devlin, A. Oaknin, J. Kerger, J. M. Lopez-Picazo, J. P. Machiels, J. P. Delord, T. R. J. Evans, V. Boni, E. Calvo, S. L. Topalian, T. Chen, I. Soumaoro, B. Li, J. Gu, R. Zwirtes and K. N. Moore (2019). "Safety and Efficacy of Nivolumab Monotherapy in Recurrent or Metastatic Cervical, Vaginal, or Vulvar Carcinoma: Results From the Phase I/II CheckMate 358 Trial." J Clin Oncol **37**(31): 2825-2834.

Novara, G., A. Galfano, V. De Marco, W. Artibani and V. Ficarra (2007). "Prognostic factors in squamous cell carcinoma of the penis." Nat Clin Pract Urol **4**(3): 140-146.

Orillion, A., A. Hashimoto, N. Damayanti, L. Shen, R. Adelaiye-Ogala, S. Arisa, S. Chintala, P. Ordentlich, C. Kao, B. Elzey, D. Gabrilovich and R. Pili (2017). "Entinostat Neutralizes Myeloid-Derived Suppressor Cells and Enhances the Antitumor Effect of PD-1 Inhibition in Murine Models of Lung and Renal Cell Carcinoma." Clin Cancer Res **23**(17): 5187-5201.

Osborne, M. C., J. Maykel, E. K. Johnson and S. R. Steele (2014). "Anal squamous cell carcinoma: an evolution in disease and management." World J Gastroenterol **20**(36): 13052-13059.

Ott, P. A., Y. J. Bang, S. A. Piha-Paul, A. R. A. Razak, J. Bennouna, J. C. Soria, H. S. Rugo, R. B. Cohen, B. H. O'Neil, J. M. Mehnert, J. Lopez, T. Doi, E. M. J. van Brummelen, R. Cristescu, P. Yang, K. Emancipator, K. Stein, M. Ayers, A. K. Joe and J. K. Lunceford (2019). "T-Cell-Inflamed Gene-Expression Profile, Programmed Death Ligand 1 Expression, and Tumor Mutational Burden Predict Efficacy in Patients Treated With Pembrolizumab Across 20 Cancers: KEYNOTE-028." J Clin Oncol **37**(4): 318-327.

Rodriguez, C. P., Q. V. Wu, J. Voutsinas, J. R. Fromm, X. Jiang, V. G. Pillarisetty, S. M. Lee, R. Santana-Davila, B. Goulart, C. S. Baik, L. Q. M. Chow, K. Eaton and R. Martins (2020). "A Phase II Trial of Pembrolizumab and Vorinostat in Recurrent Metastatic Head and Neck Squamous Cell Carcinomas and Salivary Gland Cancer." Clin Cancer Res **26**(4): 837-845.

Seiwert, T. Y., B. Burtness, R. Mehra, J. Weiss, R. Berger, J. P. Eder, K. Heath, T. McClanahan, J. Lunceford, C. Gause, J. D. Cheng and L. Q. Chow (2016). "Safety and clinical activity of pembrolizumab for treatment of recurrent or metastatic squamous cell carcinoma of the head and neck (KEYNOTE-012): an open-label, multicentre, phase 1b trial." Lancet Oncol **17**(7): 956-965.

Wang, W., S. Wang, X. Chu, H. Liu and M. Xiang (2019). "Predicting the Lung Squamous Cell Carcinoma Diagnosis and Prognosis Markers by Unique DNA Methylation and Gene Expression Profiles." J Comput Biol.

18. APPENDICES

- Appendix 1 - Performance status evaluation – WHO scale
- Appendix 2 - World Medical Association - Declaration of Helsinki
- Appendix 3ICH Harmonised Tripartite Guideline for Good Clinical Practice (ICH-GCP)
- Appendix 4 - **List of medications with (a) defined risk, (b) possible, and (c) conditional risk of Torsade de Pointes**
- Appendix 5 - Corticosteroid Dose Equivalents
- Appendix 6 - A brief summary of tumor classification RECIST v1.1
- Appendix 7 - Description of the iRECIST Process for Assessment of Disease Progression
- Appendix 8 -Toxicity criteria (NCI-CTCAE)
- Appendix 9 - Dose modification and toxicity management guidelines for immune-related AEs associated with pembrolizumab
- Appendix 10 - Dose modification and toxicity management of infusion-reactions related to pembrolizumab
- Appendix 11. Dose modification and toxicity management of drug reactions related to vorinostat
- Appendix 12 - Translational studies

Appendix 1. Performance status evaluation – WHO scale

**Performance status**<br>**ECOG-ZUBROD/ WHO**	**Grade**
Fully active, able to carry on all pre-disease performance without Restriction.	0
Restricted in physically strenuous activity but ambulatory and able to carry out work of a light or sedentary nature, e.g. light house work, office work.	1
Ambulatory and capable of all self-care but unable to carry out any work activities. Up and about more than 50% of waking hours.	2
Capable of only limited self-care, confined to bed or chair more than 50% of waking hours.	3
Completely disabled. Cannot carry on any self-care. Totally confined to bed or chair.	4
Dead	5

Developed by the Eastern Cooperative Oncology Group and published by Oken M, Creech R, Tormey D, et al. Toxicity and response criteria of the Eastern Cooperative Oncology Group. Am J Clin Oncol. 1982; 5:649-655.

Appendix 2. World Medical Association - Declaration of Helsinki

The current Declaration of Helsinki can be found on the World Medical Association web page via the link provided below:

https://www.wma.net/

Appendix 3. ICH Harmonised Tripartite Guideline for Good Clinical Practice (ICH-GCP)

The current ICH-GCP can be found on the European Medicine Agency web page via the link provided below:

https://www.ema.europa.eu/

Appendix 4. List of medications with (a) defined risk, (b) possible, and (c) conditional risk of Torsade de Pointes

https://crediblemeds.org/index.php/login/dlcheck

Woosley, RL, Heise, CW and Romero, KA, www.Crediblemeds.org, QTdrugs List, Accession Date, AZCERT, Inc. 1822 Innovation Park Dr., Oro Valley, AZ 85755

Appendix 5. Corticosteroid Dose Equivalents

Equivalent Dose	Steroid
1.2 mg	Betamethasone (long-acting)
1.5 mg	Dexamethasone (long-acting)
8 mg	Methylprednisolone (intermediate-acting)
8 mg	Triamcinolone (intermediate-acting)
10 mg	Prednisone (intermediate-acting)
10 mg	Prednisolone (intermediate-acting)
40 mg	Hydrocortisone (short-acting)
50 mg	Cortisone (short-acting)

Mager DE, Lin SX, Blum RA, Lates CD, Jusko WJ. Dose equivalency evaluation of major corticosteroids: pharmacokinetics and cell trafficking and cortisol dynamics. *J Clin Pharmacol*. 2003 Nov. 43(11):1216-27..
Webb R, Singer M. Oxford Handbook of Critical Care. Oxford University Press. 2005.

Appendix 6. A brief summary of tumor classification RECIST v1.1

(Eur. J. Cancer, 45(2009), 228-247 [47])

Full article available at: **https://ctep.cancer.gov/**

"New response evaluation criteria in solid tumours: Revised RECIST guideline (version 1.1)" E.A. Eisenhauer, P. Therasse, J. Bogaerts, L.H. Schwartz, D. Sargent, R. Ford, J. Dancey, S. Arbuck, S. Gwyther, M. Mooney, L. Rubinstein, L. Shankar, L. Dodd, R. Kaplan, D. Lacombe, J. Verweij.

Summary:

Measurability of tumor at baseline:

At baseline, tumor lesions/lymph nodes will be categorized measurable or non-measurable as follows:

Measurable

Tumor lesions: Must be accurately measured in a least one dimension (longest diameter in the plane of measurement is to be recorded) with a minimum size of:

- ≥10 mm by CT scan (CT scan slice thickness no greater than 5 mm.
- ≥10 mm caliper measurement by clinical exam.
- 20 mm by chest (=X-ray).

Malignant lymph nodes: To be considered pathologically enlarged and measurable, a lymph node must be ≥15 mm in short axis when assessed by CT scan (CT scan slice thickness recommended to be no greater than 5 mm). At baseline and in follow-up, only the short axis will be measured and followed.

Non-measurable

All other lesions, including small lesions (longest diameter <10 mm or pathological lymph nodes with ≥10 to <15 mm short axis) as well as truly non-measurable lesions. Lesions considered truly non-measurable include: leptomeningeal disease, ascites, pleural or pericardial effusion, inflammatory breast disease, lymphangitic involvement of skin or lung, abdominal masses/abdominal organomegaly identified by physical exam that is not measurable by reproducible imaging techniques.

Remark:

Bone lesions, cystic lesions, and lesions previously treated with local therapy require special considerations regarding lesion measurability (see below):

Bone lesions:

- Bone scan, PET scan or plain films are not considered adequate imaging techniques to measure bone lesions. However, these techniques can be used to confirm the presence or disappearance of bone lesions.
- Lytic bone lesions or mixed lytic-blastic lesions, with identifiable soft tissue components, that can be evaluated by cross sectional imaging techniques such as CT or MRI can be considered as measurable lesions if the soft tissue component meets the definition of measurability described above.
- Blastic bone lesions are non-measurable.

Target lesions

When more than one measurable lesion is present at baseline all lesions up to a **maximum of five lesions total (and a maximum of two lesions per organ)** representative of all involved organs should be identified as target lesions and will be recorded and measured at baseline. Target lesions should be selected on the basis of their size (lesions with the longest diameter), be representative of all involved organs, but in addition should be those that lend themselves to reproducible repeated measurements. Pathological nodes which are defined as measurable and may be identified as target lesions must meet the criterion of a short axis of ≥ 15 mm by CT scan.

The baseline sum diameters will be used as reference to further characterise any objective tumour regression in the measurable dimension of the disease.

Non-target lesions

All other lesions (or sites of disease) including pathological lymph nodes should be identified as non-target lesions and should also be recorded at baseline. Measurements are not required and these lesions should be followed as 'present', 'absent', or in rare cases 'unequivocal progression' during the trial.

Response criteria

Target lesions

Complete Response (CR): Disappearance of all target lesions. Any pathological lymph nodes (whether target or non-target) must have reduction in short axis to <10 mm.

Warning: lymph nodes identified as target lesions should always have the actual short axis measurement recorded (measured in the same anatomical plane as the baseline examination), even if the nodes regress to below 10 mm on trial. This means that when lymph nodes are included as target lesions, the 'sum' of lesions may not be zero even if complete response criteria are met, since a normal lymph node is defined as having a short axis of <10 mm. In order to qualify for CR, each node must achieve a short axis <10 mm.

Partial Response (PR): At least a 30% decrease in the sum of diameters of target lesions, taking as reference the baseline sum diameters.

Progressive Disease (PD): At least a 20% increase in the sum of diameters of target lesions, taking as reference the smallest sum on study (this includes the baseline sum if that is the smallest on study). In addition to the relative increase of 20%, the sum must also demonstrate an absolute increase of at least 5 mm. (Note: the appearance of one or more new lesions is also considered progression).

Warning: when a progression is recorded with respect to the Nadir but there is a response with respect to baseline, progression must be considered.

Stable Disease (SD): Neither sufficient shrinkage to qualify for PR nor sufficient increase to qualify for PD, taking as reference the smallest sum diameters while on trial.

Non-target lesions

Complete Response (CR): Disappearance of all non-target lesions and normalisation of tumour marker level. All lymph nodes must be non-pathological in size (<10 mm short axis).

Non-CR/Non-PD: Persistence of one or more non-target lesion(s) and/or maintenance of tumour marker level above the normal limits.

Progressive Disease (PD): Unequivocal progression (see comments below) of existing non-target lesions. (Note: the appearance of one or more new lesions is also considered progression).

Overall response:

Target lesions	Non-target lesions	New lesions		Overall response
CR	CR	No	=	**CR**
CR	Non CR/Non PD	No	=	**PR**
CR	Not evaluated	No	=	**PR**
PR	Non PD or not all evaluated	No	=	**PR**
SD	Non PD or not all evaluated	No	=	**SD**
Not all evaluated	Non PD	No	=	**Not-evaluable**
PD	No change	Yes or No	=	**PD**
No change	PD	Yes or No	=	**PD**
No change	No change	Yes	=	**PD**

Special considerations regarding baseline lesion measurability

Bone lesions:

- Bone scan, PET scan or plain films are not considered adequate imaging techniques to measure bone lesions. However, these techniques can be used to confirm the presence or disappearance of bone lesions.
- Lytic bone lesions or mixed lytic-blastic lesions, with identifiable soft tissue components, that can be evaluated by cross sectional imaging techniques such as CT or MRI can be considered as measurable lesions if the soft tissue component meets the definition of measurability described above.

Cystic lesions:

- Lesions that meet the criteria for radiographically defined simple cysts should not be considered as malignant lesions (neither measurable nor non-measurable) since they are, by definition, simple cysts.
- 'Cystic lesions' thought to represent cystic metastases can be considered as measurable lesions, if they meet the definition of measurability described above. However, if non-cystic lesions are present in the same patient, these are preferred for selection as target lesions.

Lesions with prior local treatment:

- Tumour lesions situated in a previously irradiated area, or in an area subjected to other loco-regional therapy, are usually not considered measurable unless there has been demonstrated progression in the lesion. Trial protocols should detail the conditions under which such lesions would be considered measurable.

Appendix 7. Description of the iRECIST Process for Assessment of Disease Progression

1. Assessment at Screening and Prior to RECIST v1.1 Progression

Until radiographic progression based on RECIST v1.1, there is no distinct iRECIST assessment.

2. Assessment and Decision at RECIST v1.1 Progression

In participants who show evidence of radiological PD by RECIST v1.1 the Investigator will decide whether to continue a participant on study treatment until repeat imaging is obtained (using iRECIST for participant management. This decision by the Investigator should be based on the participant's overall clinical condition.

Clinical stability is defined as the following:

- Absence of symptoms and signs indicating clinically significant progression of disease
- No decline in ECOG performance status
- No requirements for intensified management, including increased analgesia, radiation, or other palliative care

Any participant deemed clinically unstable should be discontinued from study treatment at site-assessed first radiologic evidence of PD, and is not required to have repeat tumor imaging for confirmation of PD by iRECIST.

If the Investigator decides to continue treatment, the participant may continue to receive study treatment and the tumor assessment should be repeated 4 to 8 weeks later to confirm PD by iRECIST, per Investigator assessment. I

Tumor flare may manifest as any factor causing radiographic progression per RECIST v1.1, including:

- Increase in the sum of diameters of target lesion(s) identified at baseline to ≥ 20% and ≥ 5 mm from nadir
    - Please note: the iRECIST publication uses the terminology "sum of measurements", but "sum of diameters" will be used in this protocol, consistent with the original RECIST v1.1 terminology.
- Unequivocal progression of non-target lesion(s) identified at baseline
- Development of new lesion(s)

iRECIST defines new response categories, including iUPD (unconfirmed progressive disease) and iCPD (confirmed progressive disease). For purposes of iRECIST assessment, the first visit showing progression according to RECIST v1.1 will be assigned a visit (overall) response of iUPD, regardless of which factors caused the progression.

At this visit, target and non-target lesions identified at baseline by RECIST v1.1 will be assessed as usual.

New lesions will be classified as measurable or non-measurable, using the same size thresholds and rules as for baseline lesion assessment in RECIST v1.1. From measurable new lesions, up to 5 lesions total (up to 2 per organ), may be selected as New Lesions – Target. The sum of diameters of these lesions will be calculated, and kept distinct from the sum of diameters for target

lesions at baseline. All other new lesions will be followed qualitatively as New Lesions – Non-target.

Assessment at the Confirmatory Imaging

On the confirmatory imaging, the participant will be classified as progression confirmed (with an overall response of iCPD), or as showing persistent unconfirmed progression (with an overall response of iUPD), or as showing disease stability or response (iSD/iPR/iCR).

Confirmation of Progression

Progression is considered confirmed, and the overall response will be iCPD, if ANY of the following occurs:

- Any of the factors that were the basis for the initial iUPD show worsening
  - For target lesions, worsening is a further increase in the sum of diameters of ≥ 5 mm, compared to any prior iUPD time point
  - For non-target lesions, worsening is any significant growth in lesions overall, compared to a prior iUPD time point; this does not have to meet the “unequivocal” standard of RECIST 1.1
  - For new lesions, worsening is any of these:
    - An increase in the new lesion sum of diameters by ≥ 5 mm from a prior iUPD time point
    - Visible growth of new non-target lesions
    - The appearance of additional new lesions
- Any new factor appears that would have triggered PD by RECIST 1.1

Persistent iUPD

Progression is considered not confirmed, and the overall response remains iUPD, if:

- None of the progression-confirming factors identified above occurs AND
- The target lesion sum of diameters (initial target lesions) remains above the initial PD threshold (by RECIST v1.1)

Additional imaging for confirmation should be scheduled 4 to 8 weeks from the scan on which iUPD is seen. This may correspond to the next visit in the original visit schedule. The assessment of the subsequent confirmation scan proceeds in an identical manner, with possible outcomes of iCPD, iUPD, and iSD/iPR/iCR.

Resolution of iUPD

Progression is considered not confirmed, and the overall response becomes iSD/iPR/iCR, if:

- None of the progression-confirming factors identified above occurs, AND
- The target lesion sum of diameters (initial target lesions) is not above the initial PD threshold.

The response is classified as iSD or iPR (depending on the sum of diameters of the target lesions), or iCR if all lesions resolve.

In this case, the initial iUPD is considered to be pseudo-progression, and the level of suspicion for progression is “reset”. This means that the next visit that shows radiographic progression, whenever it occurs, is again classified as iUPD by iRECIST, and the confirmation process is repeated before a response of iCPD can be assigned.

3. Management Following the Confirmatory Imaging

If repeat imaging does not confirm PD per iRECIST, as assessed by the Investigator, and the participant continues to be clinically stable, study treatment may continue and follow the regular imaging schedule. If PD is confirmed, participants will be discontinued from study treatment.

NOTE: If a participant has confirmed radiographic progression (iCPD) as defined above, but the participant is achieving a clinically meaningful benefit, an exception to continue study treatment may be considered. In this case, if study treatment is continued, tumor imaging should continue to be performed following the intervals as outlined in Section 6.

4. Detection of Progression at Visits After Pseudo-progression Resolves

After resolution of pseudo-progression (i.e., achievement of iSD/iPR/iCR), iUPD is indicated by any of the following events:

- Target lesions
    - Sum of diameters reaches the PD threshold (≥ 20% and ≥ 5 mm increase from nadir) either for the first time, or after resolution of previous pseudo-progression. The nadir is always the smallest sum of diameters seen during the entire trial, either before or after an instance of pseudo-progression.
- Non-target lesions
    - If non-target lesions have never shown unequivocal progression, their doing so for the first time results in iUPD.
    - If non-target lesions had shown previous unequivocal progression, and this progression has not resolved, iUPD results from any significant further growth of non-target lesions, taken as a whole.
- New lesions
    - New lesions appear for the first time
    - Additional new lesions appear
    - Previously identified new target lesions show an increase of ≥ 5 mm in the new lesion sum of diameters, from the nadir value of that sum
    - Previously identified non-target lesions show any significant growth

If any of the events above occur, the overall response for that visit is iUPD, and the iUPD evaluation process (see Assessment at the Confirmatory Imaging above) is repeated. Progression must be confirmed before iCPD can occur.

The decision process is identical to the iUPD confirmation process for the initial PD, except in one respect. If new lesions occurred at a prior instance of iUPD, and at the confirmatory scan the burden of new lesions has increased from its smallest value (for new target lesions, their sum of diameters is ≥5 mm increased from its nadir), then iUPD cannot resolve to iSD or iPR. It will remain iUPD until either a decrease in the new lesion burden allows resolution to iSD or iPR, or until a confirmatory factor causes iCPD.

Additional details about iRECIST are provided in the iRECIST publication [Seymour et al, 2017].

iRECIST information: http://www.eortc.org/recist/irecist/

	Clinically Stable		Clinically Unstable	
	**Imaging**	**Treatment**	**Imaging**	**Treatment**
First radiologic evidence of PD by RECIST v1.1	Repeat imaging at 4 to 8 weeks to confirm PD.	May continue study treatment at the Investigator's discretion while awaiting confirmatory tumor imaging by site by iRECIST.	Repeat imaging at 4 to 8 weeks to confirm PD per Investigator's discretion only.	Discontinue treatment
Repeat tumor imaging confirms PD (iCPD) by iRECIST per Investigator assessment	No additional imaging required.	Discontinue treatment (exception is possible upon consultation with Sponsor).	No additional imaging required.	Not applicable
Repeat tumor imaging shows iUPD by iRECIST per Investigator assessment	Repeat imaging at 4 to 8 weeks to confirm PD. May occur at next regularly scheduled imaging visit.	Continue study treatment at the Investigator's discretion.	Repeat imaging at 4 to 8 weeks to confirm PD per Investigator's discretion only.	Discontinue treatment
Repeat tumor imaging shows iSD, iPR, or iCR by iRECIST per Investigator assessment.	Continue regularly scheduled imaging assessments.	Continue study treatment at the Investigator's discretion.	Continue regularly scheduled imaging assessments.	May restart study treatment if condition has improved and/or clinically stable per Investigator's discretion. Next tumor image should occur according to the regular imaging schedule.

iCPD = iRECIST confirmed progressive disease; iCR = iRECIST complete response; iRECIST = modified Response Evaluation Criteria in Solid Tumors 1.1 for immune-based therapeutics; iSD = iRECIST stable disease; iUPD = iRECIST unconfirmed progressive disease; PD = progressive disease; RECIST v1.1 = Response Evaluation Criteria in Solid Tumors 1.1..

Appendix 8. Toxicity criteria (NCI-CTCAE)

In the present trial, adverse events will be recorded according to the

Common Terminology Criteria for Adverse Events (CTCAE) Version 5.0, *(Published: November 27, 2017)*

Toxicity evaluation scale provided separately in attached documents or download it from the NCI website

Cancer Therapy Evaluation Program

https://ctep.cancer.gov/

Appendix 9. Dose modification and toxicity management guidelines for immune-related AEs associated with pembrolizumab

General instructions:

1. Corticosteroid taper should be initiated upon AE improving to Grade 1 or less and continue to taper over at least 4 weeks.
2. For situations where pembrolizumab has been withheld, pembrolizumab can be resumed after AE has been reduced to Grade 1 or 0 and corticosteroid has been tapered. Pembrolizumab should be permanently discontinued if AE does not resolve within 12 weeks of last dose or corticosteroids cannot be reduced to ≤10 mg prednisone or equivalent per day within 12 weeks.
3. For severe and life-threatening immune-related adverse events (irAEs), IV corticosteroid should be initiated first followed by oral steroid. Other immunosuppressive treatment should be initiated if irAEs cannot be controlled by corticosteroids.

Immune-related AEs	Toxicity grade or conditions (CTCAEv5.0)	Action taken to pembrolizumab	irAE management with corticosteroid and/or other therapies	Monitor and follow-up
Pneumonitis	Grade 2	Withhold	• Administer corticosteroids (initial dose of 1-2 mg/kg prednisone or equivalent) followed by taper	• Monitor participants for signs and symptoms of pneumonitis<br>• Evaluate participants with suspected pneumonitis with radiographic imaging and initiate corticosteroid treatment<br>• Add prophylactic antibiotics for opportunistic infections
	Grade 3 or 4, or recurrent Grade 2	Permanently discontinue		
Diarrhea / Colitis	Grade 2 or 3	Withhold	• Administer corticosteroids (initial dose of 1-2 mg/kg prednisone or equivalent) followed by taper	• Monitor participants for signs and symptoms of enterocolitis (ie, diarrhea, abdominal pain, blood or mucus in stool with or without fever) and of bowel perforation (ie, peritoneal signs and ileus).<br>• Participants with ≥ Grade 2 diarrhea suspecting colitis should consider GI consultation and performing endoscopy to rule out colitis.<br>• Participants with diarrhea/colitis should be advised to drink liberal quantities of clear fluids. If sufficient oral fluid intake
	Recurrent Grade 3<br>Grade 4	Permanently discontinue		

Immune-related AEs	Toxicity grade or conditions (CTCAEv5.0)	Action taken to pembrolizumab	irAE management with corticosteroid and/or other therapies	Monitor and follow-up
				is not feasible, fluid and electrolytes should be substituted via IV infusion.
AST/ALT elevation or Increased bilirubin	Grade 2	Withhold	• Administer corticosteroids (initial dose of 0.5- 1 mg/kg prednisone or equivalent) followed by taper	• Monitor with liver function tests (consider weekly or more frequently until liver enzyme value returned to baseline or is stable
	Grade 3 or 4	Permanently discontinue	• Administer corticosteroids (initial dose of 1-2 mg/kg prednisone or equivalent) followed by taper	
Type 1 diabetes mellitus (T1DM) or Hyperglycemia [1]	Newly onset T1DM or Grade 3 or 4 hyperglycemia associated with evidence of β-cell failure	Withhold	• Initiate insulin replacement therapy for participants with T1DM<br>• Administer anti-hyperglycemic in participants with hyperglycemia	• Monitor participants for hyperglycemia or other signs and symptoms of diabetes.
Hypophysitis [1]	Grade 2	Withhold	• Administer corticosteroids and initiate hormonal replacements as clinically indicated.	• Monitor for signs and symptoms of hypophysitis (including hypopituitarism and adrenal insufficiency).
	Grade 3 or 4	Withhold or permanently discontinue [2]		
Hyperthyroidism [1]	Grade 2	Continue	• Treat with non-selective beta-blockers (eg, propranolol) or thionamides as appropriate.	• Monitor for signs and symptoms of thyroid disorders.
	Grade 3 or 4	Withhold or permanently discontinue [2]		
Hypothyroidism [1]	Grade 2-4	Continue	• Initiate thyroid replacement hormones (eg, levothyroxine or liothyroinine) per standard of care	• Monitor for signs and symptoms of thyroid disorders.

Immune-related AEs	Toxicity grade or conditions (CTCAEv5.0)	Action taken to pembrolizumab	irAE management with corticosteroid and/or other therapies	Monitor and follow-up
Nephritis and Renal dysfunction	Grade 2	Withhold	• Administer corticosteroids (prednisone 1-2 mg/kg or equivalent) followed by taper.	• Monitor changes of renal function
	Grade 3 or 4	Permanently discontinue		
Myocarditis	Grade 1 or 2	Withhold	• Based on severity of AE administer corticosteroids	• Ensure adequate evaluation to confirm etiology and/or exclude other causes
	Grade 3 or 4	Permanently discontinue		
All other immune-related AEs	Intolerable/persistent Grade 2	Withhold	• Based on type and severity of AE administer corticosteroids	• Ensure adequate evaluation to confirm etiology and/or exclude other causes
	Grade 3	Withhold or discontinue based on the type of event. Events that require discontinuation include and not limited to: Gullain-Barre Syndrome, encephalitis		
	Grade 4 or recurrent Grade 3	Permanently discontinue		

1. For participants with Grade 3 or 4 immune-related endocrinopathy where withhold of pembrolizumab is required, pembrolizumab may be resumed when AE resolves to ≤ Grade 2 and is controlled with hormonal replacement therapy or achieved metabolic control (in case of T1DM).
2. Withhold or permanently discontinue pembrolizumab is at the discretion of the investigator or treating physician.

Appendix 10. Dose modification and toxicity management of infusion-reactions related to pembrolizumab

Pembrolizumab may cause severe or life threatening infusion-reactions including severe hypersensitivity or anaphylaxis. Signs and symptoms usually develop during or shortly after drug infusion and generally resolve completely within 24 hours of completion of infusion. Dose modification and toxicity management guidelines on pembrolizumab associated infusion reaction the table below.

NCI CTCAE Grade	Treatment	Premedication at Subsequent Dosing
**Grade 1**<br>Mild reaction; infusion interruption not indicated; intervention not indicated	Increase monitoring of vital signs as medically indicated until the participant is deemed medically stable in the opinion of the investigator.	None
**Grade 2**<br>Requires therapy or infusion interruption but responds promptly to symptomatic treatment (e.g., antihistamines, NSAIDs, narcotics, IV fluids); prophylactic medications indicated for ≤24 hours	**Stop Infusion.**<br>Additional appropriate medical therapy may include but is not limited to:<br>• IV fluids<br>• Antihistamines<br>• NSAIDs<br>• Acetaminophen<br>• Narcotics<br>Increase monitoring of vital signs as medically indicated until the participant is deemed medically stable in the opinion of the investigator.<br>If symptoms resolve within 1 hour of stopping drug infusion, the infusion may be restarted at 50% of the original infusion rate (e.g. from 100 mL/hr to 50 mL/hr). Otherwise dosing will be held until symptoms resolve and the participant should be premedicated for the next scheduled dose.<br>**Participants who develop Grade 2 toxicity despite adequate premedication should be permanently discontinued from further study drug treatment.**	Participant may be premedicated 1.5 hours (± 30 minutes) prior to infusion of pembrolizumab with:<br>• Diphenhydramine 50 mg orally (or equivalent dose of antihistamine).<br>• Acetaminophen 500-1000 mg orally (or equivalent dose of analgesic).

NCI CTCAE Grade	Treatment	Premedication at Subsequent Dosing
**Grades 3 or 4**<br>Grade 3:<br>Prolonged (i.e., not rapidly responsive to symptomatic medication and/or brief interruption of infusion); recurrence of symptoms following initial improvement; hospitalization indicated for other clinical sequelae (e.g., renal impairment, pulmonary infiltrates)<br>Grade 4:<br>Life-threatening; pressor or ventilatory support indicated	**Stop Infusion.**<br>Additional appropriate medical therapy may include but is not limited to:<br>• Epinephrine**<br>• IV fluids<br>• Antihistamines<br>• NSAIDs<br>• Acetaminophen<br>• Narcotics<br>• Oxygen<br>• Pressors<br>• Corticosteroids<br>Increase monitoring of vital signs as medically indicated until the participant is deemed medically stable in the opinion of the investigator.<br>Hospitalization may be indicated.<br>**Participant is permanently discontinued from further study drug treatment.**	No subsequent dosing

Appropriate resuscitation equipment should be available at the bedside and a physician readily available during the period of drug administration.
For further information, please refer to the Common Terminology Criteria for Adverse Events v5.0 (Appendix 8).
** In cases of anaphylaxis, epinephrine should be used immediately.

Appendix 11. Dose modification and toxicity management of drug reactions related to vorinostat

ADVERSE REACTION	MANAGEMENT GUIDELINES AND DOSE MODIFICATION ALGORITHMS
**Non hematologic toxicity**	
Grade 1	Continue at the same dose level
Grade 2	Continue at the same dose level. Consider introducing supportive therapies or concomitant medications if appropriate
Grade 3 or 4<br>(except for alopecia, nausea, vomiting, or diarrhea adequately controlled with systemic antiemetic/antidiarrheal medication)	• 1st occurrence : withhold current dose for up to 14 days until toxicity improves to grade ≤ 2 and then resume to the same dose or lower dose<br>• 2nd occurrence at the same dose: withhold current dose for up to 14 days until toxicity improves to grade ≤ 2 and then resume treatment at a lower dose<br>• If the patient continues to experience toxicity, additional dose interruption and reduction steps should be considered in accordance with Table 2 - Summary table for vorinostat dose reductions.<br>• If a patient continues to experience toxicity at the lower dose or if dosing with vorinostat is interrupted for >14 consecutive days due to toxicity, under optimal supportive therapy, then treatment should be permanently discontinued and patient observed until resolution
**Hematologic toxicity**	
Grade 1	Continue at the same dose level
Grade 2	
Platelet count decreased	• Withhold current dose for up to 14 days until toxicity improves to grade ≤ 1 and then resume treatment at a lower dose.<br>• 2nd occurrence: withhold current dose for up to 14 days until toxicity improves to grade ≤ 1 and then resume treatment at a lower dose..<br>• If a patient continues to experience toxicity at the lower dose or if dosing with vorinostat is interrupted for >14 consecutive days due to toxicity, under optimal supportive therapy, then treatment should be permanently discontinued and patient observed until resolution
Other hematological toxicities	Continue at the same dose level.
Grade 3 or 4	
Platelet count decreased	• Withhold current dose for up to 14 days until toxicity improves to grade ≤ 1 and then resume treatment at a lower dose..<br>• 2nd occurrence: withhold current dose for up to 14 days until toxicity improves to grade ≤ 1 and then resume treatment at a lower dose .<br>If a patient continues to experience toxicity at the lower dose or if dosing with vorinostat is interrupted for >14 consecutive days due to toxicity, under optimal supportive therapy, then treatment should be permanently discontinued and pa patient observed until resolution

Other hematological toxicities	• 1st occurrence : withhold current dose for up to 14 days until toxicity improves to grade ≤ 2 and then resume to the same dose or lower dose<br>• 2nd occurrence at the same dose: withhold current dose for up to 14 days until toxicity improves to grade ≤ 2 and then resume treatment at a lower dose .<br>• If the patient continues to experience toxicity, additional dose interruption and reduction steps should be considered in accordance with Table 2 - Summary table for vorinostat dose reductions.<br>• If a patient continues to experience toxicity at the lower dose or if dosing with vorinostat is interrupted for >14 consecutive days due to toxicity, under optimal supportive therapy, then treatment should be permanently discontinued and patient observed until resolution
**Recommended criteria for starting treatment cycles**	
	• ANC ≥1.0 x 10^9/L<br>• Platelet count ≥75 x 10^9/L<br>• Non-hematologic toxicities have returned to baseline or ≤CTCAE Grade 1 severity or ≤CTCAE Grade 2 severity if not considered a safety risk for the patient.

Appendix 12. Translational studies

The translational studies will allow the generation of a comprehensive dataset with mutational, epigenomic and expression profiles of the primary tumor and quantification of the viral load for each patient in order to:

- Assess the link between the tumor molecular profile including epigenetics features and immune parameters of the tumor microenvironment (TME) and the blood.
- Assess the link between immune-related biomarkers in the TME and blood (including but not limited to tumor tissue PD-L1 expression by IHC, RNA gene expression profiling and DNA mutation analysis), and measures of efficacy.
- Explore the modification of immune-related and molecular epigenetic biomarkers following treatment.
- Analyze the impact of TME and epigenetics parameters on sensitivity/response to treatment.
- Assess the predictive value of circulating biomarkers on sensitivity or resistance to treatment.

All bioinformatics analyses will be centralized at Leipzig University in collaborations with Institut Curie. Data integration with a focus on confounding elements for the success of treatment with the pembrolizumab and vorinostat combination will be done. Confounding factors influencing the treatment's success will be translated onto biomarkers that can be easily analyzed prior to the start of a treatment. Even if there are no apparent differentially expressed genes or DMRs common to all cancer types, there are very likely common pathways that are affected.

A/ WHOLE EXOME SEQUENCING
STUDY COORDINATOR: Istituto Europeo di Oncologia (IEO)
CONTACT DETAILS: Luca Mazzarella , Department of Oncology and Hemato-Oncology - Division of Early Drug Development for Innovative Therapies, Via Ripamonti, 435, 20141, Milan , Italy. +39 0257489439, luca.mazzarella@ieo.it
OBJECTIVES: TO ASSESS :<br>1. tumor mutational load and mutational signatures,<br>2. most frequently altered genes (SNV and CNV), and<br>3. other molecular signatures (microsatellite instability [MSI], Homologous recombination deficiency [HRD], etc). Mutational signatures can point to malfunctioning mechanisms like DNA strand break repair, DNA mismatch repair or AID/APOBEC driven cytosin deamination in certain cancers. This can have an impact on treatment success. Likewise, SNVs, CNVs, MSI or HRD may also influence the applicability of certain drugs and may therefore be used as biomarkers. Molecular alterations detected at baselines will be followed in the ctDNA at different time points.
SAMPLES REQUIRED: Frozen tumors and blood (PBMC) at baseline as well as ctDNA
SAMPLE STORAGE AND COLLECTION: Collected on study site and stored at IBBL
PROCESSING OF SAMPLES: DNA will be extracted at IBBL and sent to EIO

B/ RNA SEQUENCING
STUDY COORDINATOR: Istituto Europeo di Oncologia (IEO)
CONTACT DETAILS: Luca Mazzarella , Department of Oncology and Hemato-Oncology - Division of Early Drug Development for Innovative Therapies, Via Ripamonti, 435, 20141, Milan , Italy. +39 0257489439, luca.mazzarella@ieo.it
OBJECTIVES: To assess changes in expression profile :<br>1. Study gene expression,<br>2. Identify splice variants, differential isoform usage, intron retention and fusion transcripts on both tumor and stromal components, and 3) analyse RNA editing and quantify allelic expression of SNVs and expression impact of CNVs. The existence or abundance of certain isoforms and the expression levels of certain genes are good candidates for biomarkers that can be used to choose a treatment.
SAMPLES REQUIRED: RNA from all patient' frozen tumors at baseline, during treatment and at progression
SAMPLE STORAGE AND COLLECTION: Collected on study site and stored at IBBL
PROCESSING OF SAMPLES: RNA will be extracted at IBBL and sent to EIO

C/ IHC ANALYSIS
STUDY COORDINATOR: ISTITUTO EUROPEO DI ONCOLOGIA (IEO)
CONTACT DETAILS: Luca Mazzarella, Department of Oncology and Hemato-Oncology - Division of Early Drug Development for Innovative Therapies, Via Ripamonti, 435, 20141, Milan, Italy. +39 0257489439, luca.mazzarella@ieo.it
OBJECTIVES:<br>Expression profiles at different time points will be correlated to IHC analyses on FFPE samples. Several markers, but not limited to, CD45, CD8, CD68, PD-L1, PD-L2, CD3CD8FoxP3 and CD14CD33CD163 will be performed in addition to other panels specific to a cellular compartments (dendritic cell, T cell, myeloid cell, tumor…).
SAMPLES REQUIRED: FFPE samples from all patient at baseline, during treatment and at progression
SAMPLE STORAGE AND COLLECTION: Collected on study site and stored at IBBL
PROCESSING OF SAMPLES: Slide from FFPE tumour will be prepared by IBBL and send to IEO

D/ GLOBAL AND GENOME-WIDE EPIGENETIC ANALYSIS
STUDY COORDINATOR: Istituto Europeo di Oncologia (IEO), Insitut Curie (IC)
CONTACT DETAILS:<br>Luca Mazzarella, Department of Oncology and Hemato-Oncology - Division of Early Drug Development for Innovative Therapies, Via Ripamonti, 435, 20141, Milan, Italy. +39 0257489439, luca.mazzarella@ieo.it<br><br>Christophe LE TOURNEAU, Department of Drug Development and Innovation, 26 rue d'Ulm, 75005, Paris France, +33144324675, christophe.letourneau@curie.fr

OBJECTIVES: To assess changes in expression profile :<br>Global and genome-wide epigenetic analysis (techniques to be defined later) will be performed to evaluate the possible role of key epigenetic modifications as predictors of response to investigational drugs. Methylation data will be analyzed with respect to :<br>1. Differentially methylated regions (DMRs) distinguishing cancer types.<br>2. DMRs that do not correspond to the cancer types, but partition the data into groups that consist of examples from several cancer types. Methylation data from these DMRs are candidates for a correlation to treatment success. A global methylation pattern of samples will be extracted from the data, as well as the methylation status of promoters and gene bodies.
SAMPLES REQUIRED: DNA from all patients' tumors at different time points
SAMPLE STORAGE AND COLLECTION: Collected on study site and stored at IBBL
PROCESSING OF SAMPLES: DNA will be extracted at IBBL and sent to EIO

E/ CIRCULATING HPV
STUDY COORDINATOR: Institut Curie (IC)
CONTACT DETAILS: Christophe LE TOURNEAU, Department of Drug Development and Innovation, 26 rue d'Ulm, 75005, Paris France, +33144324675, christophe.letourneau@curie.fr
OBJECTIVES: Circulating HPV DNA (HPV ctDNA from plasma samples) levels will be assessed by droplet digital dPCR (ddPCR) on HPV positive patients at different time points. The HPV ctDNA concentration will be expressed in copies/mL of plasma.
SAMPLES REQUIRED: DNA from all patients' tumors at different time points
SAMPLE STORAGE AND COLLECTION: Collected on study site and stored at IBBL
PROCESSING OF SAMPLES: DNA will be extracted at IBBL and sent to IC

F/ HLA SEQUENCING
STUDY COORDINATOR: Integrated BioBank of Luxembourg (IBBL)
CONTACT DETAILS: Kristin Kornerup, 1, Rue Louis Rech, L-3555, Dudelange, Luxembourg, Kristin.kornerup@ibbl.lu, +352 26 970 – 532
OBJECTIVES: Ultrahigh resolution sequencing of 11 HLA Loci (Class I HLA-A, -B, -C; Class II HLA-DRB1/3/4/5, -DQA1, -DQB1, -DPA1, -DPB1) will be performed at IBBL to provide important information on the patient's HLA type and an indication of the likely response to immunotherapy.
SAMPLES REQUIRED: DNA from all patients' at baseline
SAMPLE STORAGE AND COLLECTION: Collected on study site and stored at IBBL
PROCESSING OF SAMPLES: DNA will be extracted at IBBL and proceed at IBBL

CONSORT 2010 checklist of information to include when reporting a randomised trial*

Section/Topic	Item No	Checklist item	Reported on page No
**Title and abstract**			
	1a	Identification as a randomised trial in the title	
	1b	Structured summary of trial design, methods, results, and conclusions (for specific guidance see CONSORT for abstracts)	4
**Introduction**			
Background and objectives	2a	Scientific background and explanation of rationale	5-7
	2b	Specific objectives or hypotheses	6-7
**Methods**			
Trial design	3a	Description of trial design (such as parallel, factorial) including allocation ratio	19
	3b	Important changes to methods after trial commencement (such as eligibility criteria), with reasons	
Participants	4a	Eligibility criteria for participants	20-21
	4b	Settings and locations where the data were collected	
Interventions	5	The interventions for each group with sufficient details to allow replication, including how and when they were actually administered	20
Outcomes	6a	Completely defined pre-specified primary and secondary outcome measures, including how and when they were assessed	21-22
	6b	Any changes to trial outcomes after the trial commenced, with reasons	
Sample size	7a	How sample size was determined	23-24
	7b	When applicable, explanation of any interim analyses and stopping guidelines	
Randomisation:			
Sequence generation	8a	Method used to generate the random allocation sequence	
	8b	Type of randomisation; details of any restriction (such as blocking and block size)	
Allocation concealment mechanism	9	Mechanism used to implement the random allocation sequence (such as sequentially numbered containers), describing any steps taken to conceal the sequence until interventions were assigned	
Implementation	10	Who generated the random allocation sequence, who enrolled participants, and who assigned participants to interventions	
Blinding	11a	If done, who was blinded after assignment to interventions (for example, participants, care providers, those	

		assessing outcomes) and how	
	11b	If relevant, description of the similarity of interventions	
Statistical methods	12a	Statistical methods used to compare groups for primary and secondary outcomes	23-24
	12b	Methods for additional analyses, such as subgroup analyses and adjusted analyses	23-24
**Results**			
Participant flow (a diagram is strongly recommended)	13a	For each group, the numbers of participants who were randomly assigned, received intended treatment, and were analysed for the primary outcome	7
	13b	For each group, losses and exclusions after randomisation, together with reasons	7
Recruitment	14a	Dates defining the periods of recruitment and follow-up	7
	14b	Why the trial ended or was stopped	7-8 + Table 1
Baseline data	15	A table showing baseline demographic and clinical characteristics for each group	Table 1
Numbers analysed	16	For each group, number of participants (denominator) included in each analysis and whether the analysis was by original assigned groups	7
Outcomes and estimation	17a	For each primary and secondary outcome, results for each group, and the estimated effect size and its precision (such as 95% confidence interval)	8-12
	17b	For binary outcomes, presentation of both absolute and relative effect sizes is recommended	
Ancillary analyses	18	Results of any other analyses performed, including subgroup analyses and adjusted analyses, distinguishing pre-specified from exploratory	8-12
Harms	19	All important harms or unintended effects in each group (for specific guidance see CONSORT for harms)	
**Discussion**			
Limitations	20	Trial limitations, addressing sources of potential bias, imprecision, and, if relevant, multiplicity of analyses	12-19
Generalisability	21	Generalisability (external validity, applicability) of the trial findings	12-19
Interpretation	22	Interpretation consistent with results, balancing benefits and harms, and considering other relevant evidence	12-19
**Other information**			
Registration	23	Registration number and name of trial registry	20 + abstract
Protocol	24	Where the full trial protocol can be accessed, if available	Protocol attached
Funding	25	Sources of funding and other support (such as supply of drugs), role of funders	28

*We strongly recommend reading this statement in conjunction with the CONSORT 2010 Explanation and Elaboration for important clarifications on all the items. If relevant, we also recommend reading CONSORT extensions for cluster randomised trials, non-inferiority and equivalence trials, non-pharmacological treatments, herbal interventions, and pragmatic trials. Additional extensions are forthcoming: for those and for up to date references relevant to this checklist, see www.consort-statement.org.
