# Supplementary material for: Efficacy of pembrolizumab and vorinostat combination in patients with recurrent and/or metastatic squamous cell carcinomas: a phase 2 basket trial
Source: Nat Cancer. 2025 Jun 30;6(8):1370–83. doi: 10.1038/s43018-025-01004-2 (PMC12380617; doi:10.1038/s43018-025-01004-2)
Supplement: Supplementary file 2 — Reporting Summary [file 43018_2025_1004_MOESM2_ESM.pdf]

Corresponding author(s): Pr Christophe Le Tourneau

Last updated by author(s): Apr 16, 2025

Reporting Summary

Nature Portfolio wishes to improve the reproducibility of the work that we publish. This form provides structure for consistency and transparency in reporting. For further information on Nature Portfolio policies, see our Editorial Policies and the Editorial Policy Checklist.

Statistics

For all statistical analyses, confirm that the following items are present in the figure legend, table legend, main text, or Methods section.

n/a	Confirmed	
☐	☒	The exact sample size (*n*) for each experimental group/condition, given as a discrete number and unit of measurement
☐	☒	A statement on whether measurements were taken from distinct samples or whether the same sample was measured repeatedly
☐	☒	The statistical test(s) used AND whether they are one- or two-sided<br>*Only common tests should be described solely by name; describe more complex techniques in the Methods section.*
☐	☒	A description of all covariates tested
☒	☐	A description of any assumptions or corrections, such as tests of normality and adjustment for multiple comparisons
☐	☒	A full description of the statistical parameters including central tendency (e.g. means) or other basic estimates (e.g. regression coefficient) AND variation (e.g. standard deviation) or associated estimates of uncertainty (e.g. confidence intervals)
☐	☒	For null hypothesis testing, the test statistic (e.g. *F*, *t*, *r*) with confidence intervals, effect sizes, degrees of freedom and *P* value noted<br>*Give P values as exact values whenever suitable.*
☒	☐	For Bayesian analysis, information on the choice of priors and Markov chain Monte Carlo settings
☒	☐	For hierarchical and complex designs, identification of the appropriate level for tests and full reporting of outcomes
☐	☒	Estimates of effect sizes (e.g. Cohen's *d*, Pearson's *r*), indicating how they were calculated

Our web collection on statistics for biologists contains articles on many of the points above.

Software and code

Policy information about availability of computer code

Data collection	Electronic case report Forms (eCRF) were used by the clinical trials staff to collect data from patients according to the protocol. In accordance with ICH E6GCP, the sponsor monitoring team verified the eCRF entries against source data.<br>The eCRF was developed on ENNOV CLINICAL software v8.2 solution by Institut du Cancer de Montpellier data management team, subcontractor of the sponsor. Database is hosted by AZ network.<br>The collection and analyses rely on standard bioinformatics software publicly available and widely used by the community. All details about the tools version, or the different parameters applied are discribed in the material and methods
Data analysis	Statistical analyses were carried out using STATA v16 (StataCorp, College Station, TX, USA) software.<br>The codes used for molecular analyses are available at https://github.com/bioinfo-pf-curie/PEVO. Package versions used: snpeff: v 5.1; snpsift: v 5.1; FACETS: v 0.6.1; SigProfiler: Extractor: v 1.1.21, Plotting: v 1.3.14, MatrixGenerator: v 1.2.17, Assignment: v 0.031.

For manuscripts utilizing custom algorithms or software that are central to the research but not yet described in published literature, software must be made available to editors and reviewers. We strongly encourage code deposition in a community repository (e.g. GitHub). See the Nature Portfolio guidelines for submitting code & software for further information.

Data

Policy information about availability of data

All manuscripts must include a data availability statement. This statement should provide the following information, where applicable:

- Accession codes, unique identifiers, or web links for publicly available datasets
- A description of any restrictions on data availability
- For clinical datasets or third party data, please ensure that the statement adheres to our policy

Data availability
The original files and raw NGS data generated in this study have been deposited in the EGA database under accession code EGAD50000001013 (https://ega-archive.org/studies/ EGAD50000001013). Data on EGA is under controlled access. Sequencing data will be made available upon request through EGA, and additional clinical information can be made available upon institutional approval.
Requests should be addressed to Dr Nicolas Servant (Nicolas.servant@curie.fr). The estimated timeframe for access to be granted is 2 months, and the duration will be determined according to the request needs.
The source data generated in this study are provided in the different Source Data files. All relevant clinical trial data used in this study are accessible in the Supplementary Data files and de-identified."

Research involving human participants, their data, or biological material

Policy information about studies with human participants or human data. See also policy information about sex, gender (identity/presentation), and sexual orientation and race, ethnicity and racism.

Reporting on sex and gender	Male and females with a with recurrent and/or metastatic SCC of the head and neck, cervix, lung, anus, vulva/vagina, and penis were included. Gender was reported in the patient baseline characteristics, no other sex/gender analysis was carried out.
Reporting on race, ethnicity, or other socially relevant groupings	No reports on race, ethnicity, or other socially relevant groups. The study was conducted in Europe. This aligns with common practices in France, where such distinctions are generally not made. French law tends to protect against making distinctions based on these categories except in cases where it is explicitly justified by the research objectives, which was not applicable in our case.
Population characteristics	The baseline characteristics of the whole patient population are summarized in Table 1. The majority (63%) of the patients were female, with a median age of 61 (range: 18-85) and 55% had an Eastern Cooperative Oncology Group (ECOG) performance status score of 1. The median number of prior systemic therapy lines was 1 (range: 0-4). For 53% of the vulvar/vaginal SCC patients, the investigated regimen was the first line of treatment, whereas only 17% of the patients included in the anal cohort did not receive any treatment in the recurrent/metastatic setting. Eighty-six (77.5%) patients had a metastatic disease while 25 (22.5%) patients had a loco-regional recurrence. Sixty-three (57%) patients had a HPV-positive tumor. The majority of them presented with an HPV16 type (54 [49%] of 111 patients), while only two (2%) patients had a HPV18-related disease. The Combined Positive Score (CPS) for PD-L1 status was assessed in 102 (92%) patients. Most patients (84 patients, 82%) had a CPS ≥1, and 29 (28%) had a CPS ≥20. Fifty-nine patients (58%) showed a tumor with a Tumor Proportion Score (TPS) ≥1. The Tumor Mutational Burden (TMB) was evaluated in 80 (72%) patients, 12 (15%) of them presented with a high TMB. Three (4%) of the 80 patients evaluated had a MSI-H tumor.
Recruitment	Between October 30th, 2020, and May 10th, 2022, 112 consecutive patients with recurrent and/or metastatic SCC from various locations were included and involved 29 anal, 27 HNSCC, 26 cervical, 17 vulvar/vaginal, 11 penile and 2 lung cancer patients (Figure 1). As of November 14th, 2022 (cut-off date), 111 patients received at least one dose of the treatment and 107 treated patients had at least one valid disease assessment post-baseline or progressed before a RECIST disease assessment. The anti-tumoral activity was evaluated in 107 treated patients (four patients did not have a valid disease assessment post-baseline or presented with a progressive disease).
Ethics oversight	All patients were included after written informed consent. The study was approved by the Ethics Committee of the National Institute of Pharmacy and Nutrition, and carried out in accordance with the Declaration of Helsinki, the Good Clinical Practice guidelines of the International Conference on Harmonization, and relevant French and European laws and directives.

Note that full information on the approval of the study protocol must also be provided in the manuscript.

Field-specific reporting

Please select the one below that is the best fit for your research. If you are not sure, read the appropriate sections before making your selection.

☒ Life sciences ☐ Behavioural & social sciences ☐ Ecological, evolutionary & environmental sciences

For a reference copy of the document with all sections, see nature.com/documents/nr-reporting-summary-flat.pdf

Life sciences study design

All studies must disclose on these points even when the disclosure is negative.

Sample size	ORR reported in the literature with pembrolizumab or nivolumab in SCC patients ranged from 6% to 24% depending on the primary tumor

Sample size	location.<br>The required number of evaluable patients for each cohort was determined using an A'Hern design based on different hypotheses (53). To compensate for potential drop out, an additional 10% of patients in each cohort was added; therefore, a total of 112 patients was required for this study. Number of required patients, design parameters and decision rules for each cohort are summarized in the Extended Data Table 1.
Data exclusions	Between October 30th, 2020, and May 10th, 2022, 112 patients with recurrent and/or metastatic SCC from various locations were included and involved 29 anal, 27 HNSCC, 26 cervical, 17 vulvar/vaginal, 11 penile and 2 lung cancer patients (Figure 1). As of November 14th, 2022 (cut-off date), 111 patients received at least one dose of the treatment and 107 treated patients had at least one valid disease assessment post-baseline or progressed before a RECIST disease assessment. The anti-tumoral activity was evaluated in 107 treated patients (four patients did not have a valid disease assessment post-baseline or presented with a progressive disease).<br>The primary and secondary efficacy endpoints were assessed in the per-protocol population (n=107), corresponding to all eligible patients with at least one valid post-baseline disease assessment (or with disease progression) and who had received at least one dose of the study treatments.<br>Regarding molecular analyses, 80 patients had tumor WES data, but 3 samples were removed due to low quality or not being evaluable according to the main criterion, resulting in n=77 paired tumor and constitutional WES samples analyzed.
Replication	Reproducibility in this Phase II non-randomized basket trial is ensured through a standardized protocol across all tumor cohorts, predefined eligibility criteria, consistent dosing and treatment schedules, centralized data collection, and uniform assessment criteria (e.g., RECIST v1.1). Additionally, prospective registration and detailed statistical analysis plans support transparent and replicable findings.
Randomization	This is not a randomized study. Only one treatment is evaluated and therefore no random or non-random allocation of treatment.
Blinding	no blinding was performed. Blinding was not used in this non-randomized trial due to practical and ethical considerations. The combination therapy of pembrolizumab and vorinostat requires careful monitoring and dose adjustments, making blinding impractical. Additionally, patient safety is a priority, as clinicians must manage known adverse effects effectively. The primary endpoint, objective response rate (ORR), is assessed through imaging, reducing the risk of bias compared to subjective measures. Since the study relies on investigator assessment and is not randomized, blinding would not significantly impact the validity of the results. Therefore, an open-label design was the most appropriate choice

Reporting for specific materials, systems and methods

We require information from authors about some types of materials, experimental systems and methods used in many studies. Here, indicate whether each material, system or method listed is relevant to your study. If you are not sure if a list item applies to your research, read the appropriate section before selecting a response.

Materials & experimental systems

n/a	Involved in the study
☒	☐ Antibodies
☒	☐ Eukaryotic cell lines
☒	☐ Palaeontology and archaeology
☒	☐ Animals and other organisms
☐	☒ Clinical data
☒	☐ Dual use research of concern
☒	☐ Plants

Methods

n/a	Involved in the study
☒	☐ ChIP-seq
☒	☐ Flow cytometry
☒	☐ MRI-based neuroimaging

Clinical data

Policy information about clinical studies

All manuscripts should comply with the ICMJE guidelines for publication of clinical research and a completed CONSORT checklist must be included with all submissions.

Clinical trial registration	EudraCT (N° 2019-003839-33), and ClinicalTrials.org (NCT04357873).
Study protocol	The study protocol is attached to the submission
Data collection	Between October 30th, 2020, and May 10th, 2022, 112 patients with recurrent and/or metastatic SCC from various locations were included in France and involved 29 anal, 27 HNSCC, 26 cervical, 17 vulvar/vaginal, 11 penile and 2 lung cancer patients (Figure 1). As of November 14th, 2022 (cut-off date), 111 patients received at least one dose of the treatment and 107 treated patients had at least one valid disease assessment post-baseline or progressed before a RECIST disease assessment. The anti-tumoral activity was evaluated in 107 treated patients (four patients did not have a valid disease assessment post-baseline or presented with a progressive disease).
Outcomes	The primary objective was to evaluate the antitumor activity of pembrolizumab in combination with vorinostat in patients with recurrent and/or metastatic SCC of the head and neck, cervix, lung, anus, vulva/vagina, and penis, using the ORR by investigator assessment. The ORR was defined in each cohort as the percentage of evaluable patients for ORR, designated as the proportion of patients with a CR or a PR as best response according to RECIST1.1 (52). Key secondary endpoints included DOR, PFS, OS, and the incidence of adverse events.<br>DOR was evaluated in patients with either a CR or PR and defined as the time from the first CR or PR assessment to the date of the

first occurrence of a progressive disease (PD) or death from any cause (if death occurred within predefined period), whichever came first.
PFS was defined according to RECIST1.1 as the time from inclusion to the date of disease progression or death from any cause, whichever came first. At the time of analysis, a patient alive and without disease progression was censored at the date of the last tumor assessment. Patients alive without disease progression who started a new anticancer therapy were censored at the date of the last tumor assessment prior to the start of the new anticancer therapy.
OS was defined as the time from inclusion to the time of death from any cause. Patients who were alive at last follow-up news were censored at this date.
To assess the safety, adverse events (AEs) were evaluated and reported according to the National Cancer Institute Common Terminology Criteria for Adverse Events (CTCAE) version 5.0 in each cohort and in the overall study population.
Translational endpoints aimed to assess the link between selected biomarkers and their impact on response to treatment. These biomarkers included, but were not limited to, tumor tissue PD-L1 expression (evaluated by immunohistochemistry) p16 and HPV status, the tumor mutational load assessed by whole exome sequencing, and molecular signatures (such as homologous recombination deficiency and microsatellite instability).

Plants

Seed stocks

Report on the source of all seed stocks or other plant material used. If applicable, state the seed stock centre and catalogue number. If plant specimens were collected from the field, describe the collection location, date and sampling procedures.

Novel plant genotypes

Describe the methods by which all novel plant genotypes were produced. This includes those generated by transgenic approaches, gene editing, chemical/radiation-based mutagenesis and hybridization. For transgenic lines, describe the transformation method, the number of independent lines analyzed and the generation upon which experiments were performed. For gene-edited lines, describe the editor used, the endogenous sequence targeted for editing, the targeting guide RNA sequence (if applicable) and how the editor was applied.

Authentication

Describe any authentication procedures for each seed stock used or novel genotype generated. Describe any experiments used to assess the effect of a mutation and, where applicable, how potential secondary effects (e.g. second site T-DNA insertions, mosiacism, off-target gene editing) were examined.
